# Potential inhibitors of VEGFR1, VEGFR2, and VEGFR3 developed through Deep Learning for the treatment of Cervical Cancer

**DOI:** 10.1038/s41598-024-63762-w

**Published:** 2024-06-10

**Authors:** Anuraj Nayarisseri, Mohnad Abdalla, Isha Joshi, Manasi Yadav, Anushka Bhrdwaj, Ishita Chopra, Arshiya Khan, Arshiya Saxena, Khushboo Sharma, Aravind Panicker, Umesh Panwar, Francisco Jaime Bezerra Mendonça Junior, Sanjeev Kumar Singh

**Affiliations:** 1 In silico Research Laboratory, Eminent Biosciences, 91, Sector-A, Mahalakshmi Nagar, Indore, Madhya Pradesh 452010 India; 2 Bioinformatics Research Laboratory, LeGene Biosciences Pvt Ltd, 91, Sector-A, Mahalakshmi Nagar, Indore, Madhya Pradesh 452010 India; 3 Key Laboratory of Chemical Biology (Ministry of Education), Department of Pharmaceutics, School of Pharmaceutical Sciences, Cheeloo College of Medicine, Shandong University, 44 Cultural West Road, Jinan, 250012 Shandong Province People’s Republic of China; 4 School of Medicine and Health Sciences, The George Washington University, Ross Hall, 2300 Eye Street, Washington, D.C., NW 20037 USA; 5 Computer Aided Drug Designing and Molecular Modeling Lab, Department of Bioinformatics, Alagappa University, Karaikudi, Tamil Nadu 630003 India; 6 Laboratory of Synthesis and Drug Delivery, Department of Biological Sciences, State University of Paraiba, João Pessoa, 58429-500 Brazil

**Keywords:** VEGFR inhibitors, Machine-learning, Deep learning, Molecular docking, Molecular dynamics simulation, R programming, Python, ADMET studies, Biotechnology, Cancer, Cell biology, Computational biology and bioinformatics, Drug discovery, Structural biology, Systems biology, Mathematics and computing

## Abstract

Cervical cancer stands as a prevalent gynaecologic malignancy affecting women globally, often linked to persistent human papillomavirus infection. Biomarkers associated with cervical cancer, including VEGF-A, VEGF-B, VEGF-C, VEGF-D, and VEGF-E, show upregulation and are linked to angiogenesis and lymphangiogenesis. This research aims to employ in-silico methods to target tyrosine kinase receptor proteins—VEGFR-1, VEGFR-2, and VEGFR-3, and identify novel inhibitors for Vascular Endothelial Growth Factors receptors (VEGFRs). A comprehensive literary study was conducted which identified 26 established inhibitors for VEGFR-1, VEGFR-2, and VEGFR-3 receptor proteins. Compounds with high-affinity scores, including PubChem ID—25102847, 369976, and 208908 were chosen from pre-existing compounds for creating Deep Learning-based models. RD-Kit, a Deep learning algorithm, was used to generate 43 million compounds for VEGFR-1, VEGFR-2, and VEGFR-3 targets. Molecular docking studies were conducted on the top 10 molecules for each target to validate the receptor-ligand binding affinity. The results of Molecular Docking indicated that PubChem IDs—71465,645 and 11152946 exhibited strong affinity, designating them as the most efficient molecules. To further investigate their potential, a Molecular Dynamics Simulation was performed to assess conformational stability, and a pharmacophore analysis was also conducted for indoctrinating interactions.

## Introduction

Cervical cancer, a widespread gynaecologic malignancy affecting women globally, comprises predominantly squamous cell carcinoma (80%) and adenocarcinoma (15%). Human papillomavirus (HPV) infection is the primary cause. Population-based studies highlight the vulnerability of the 35–44 age group. Early detection significantly enhances survival rates, with metastasis to lung, bone, liver, and brain tissues if untreated for an extended period^1–4^. Traditional treatments involve surgical excision, radiation, and chemotherapy, but complexities pose challenges^5^. Developing accessible medicines and screening measures for all stages of cervical cancer is crucial. The initiation of cervical cancer involves migratory-linked vascular permeability, proliferation, and survival of surrounding endothelial cells in angiogenesis and lymphangiogenesis.

**Figure 1:**
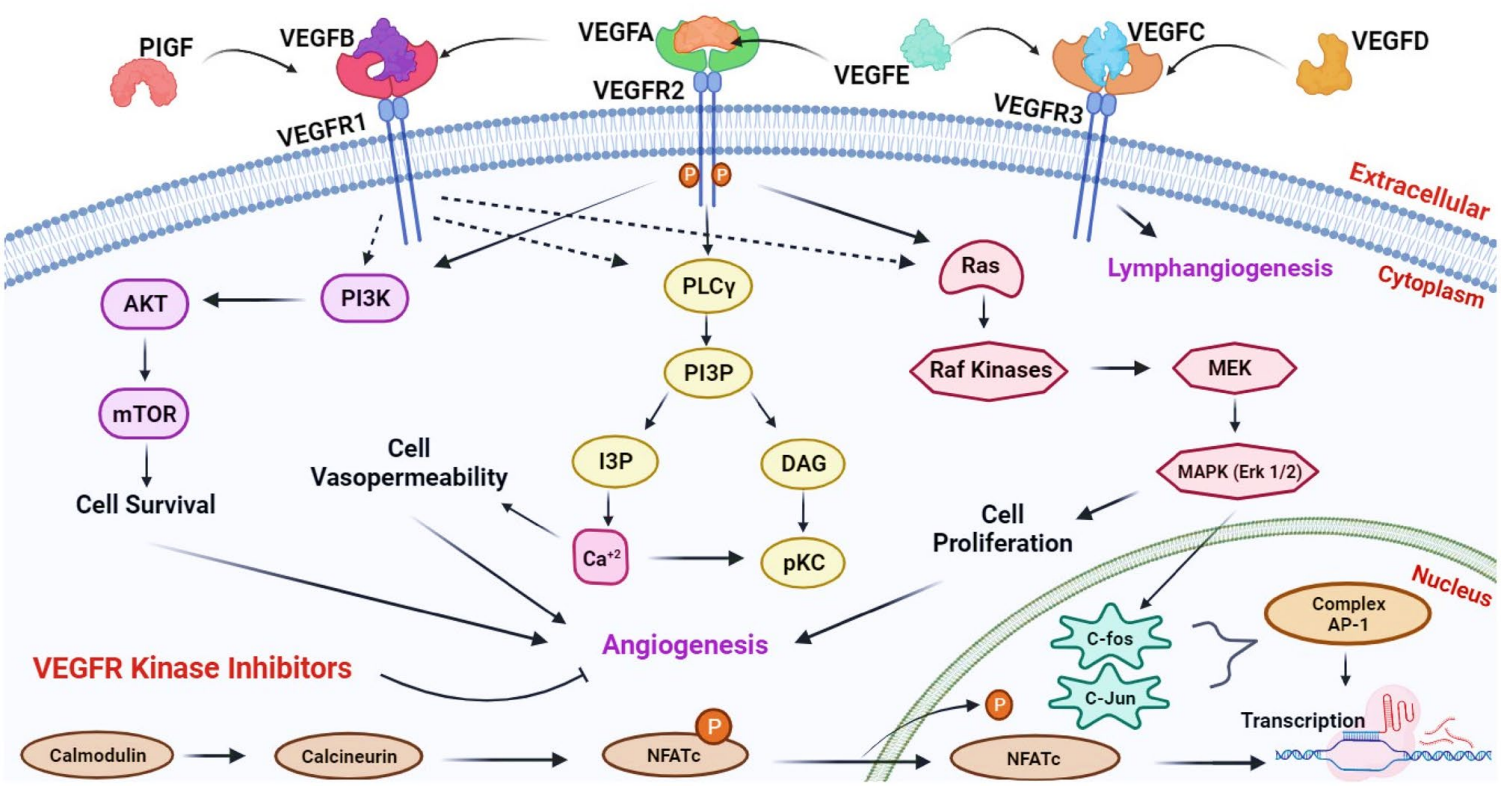
Comprehensive Illustration of the VEGFR-VEGF Signaling Pathway Mechanism inCancer Initiation and Progression.

In cervical cancer, biomarkers of the vascular endothelial growth factor family exhibit upregulation. The VEGFR proteins—VEGFR-1, VEGFR-2, and VEGFR-3—interact with VEGF proteins (VEGF-A, VEGF-B, VEGF-C, VEGF-D, and VEGF-E). These VEGFR proteins, classified as tyrosine kinase receptors (RTK), initiate autophosphorylation and dimerization upon ligand binding, activating the RTK’s cytoplasmic tyrosine kinase domain. This activation leads to transcriptional regulation of various genes, triggering a signalling cascade that induces vascular permeability (increased plasma protein leakage) and supports angiogenesis and lymphangiogenesis^6–8^ (Fig. 1).

VEGFR-1 and VEGFR-2, situated on vascular endothelial cells, drive angiogenesis, while VEGFR-3, located on lymphatic endothelial cells, participates in lymph angiogenesis. Elevated expression levels of VEGFR-1/2 are linked to cancer development and poorer overall survival. VEGFR-1 mediates distant metastasis by binding to VEGF-A, VEGF-B, and PIGF^9,10^. VEGFR-2 binds to multiple VEGF variants-A/B/C/D/E, activating signalling pathways that enhance tumor growth through increased cellular proliferation. Soluble forms of VEGFR-2 and VEGF-A are elevated in the blood of cervical cancer patients^11^.VEGFR-3 interacts with partially processed VEGF-C, VEGF-D, and VEGF-E, whose overexpression is associated with ascites development and tumor cell spread to nearby lymph nodes, facilitating cancer progression^12^. Higher circulating levels of VEGF-D in pre-invasive cervical carcinomas indicate a predilection for the lymphangiogenic pathway^13^. Chemotherapy targets tumor cells directly, causing significant damage. However, antiangiogenic drugs, which also address stromal components, exhibit a more favourable response than chemotherapy^14^. Immunotherapy using receptor antibodies, short peptides, and small molecule inhibitors has emerged as a promising therapeutic and anti-angiogenic strategy. Bevacizumab, a multi-target antibody blocking VEGF receptors, is frequently used alone or in combination with other therapies for treating cervical cancer^14^.

The significance of small molecular compounds has grown due to high manufacturing and maintenance costs, coupled with limited optimization potential for antibody-based therapy. Small molecular compounds, functioning as tyrosine kinase inhibitors, offer a targeted approach in cancer, competing for ligand binding sites with proangiogenic factor ligands. This technique presents a focused strategy for blocking angiogenesis in both early and late-stage cervical malignancies, minimizing off-target effects in clinical oncology.Higher VEGF expression levels significantly impact the prognosis of cervical cancer patients in stages IB and IIA. Moreover, the oncoproteins E5 and E6 of HPV stimulate substantial VEGF expression, underscoring their role in cervical cancer development^15–17^.

In the present investigation, a comprehensive review of the literature was carried out in which 26 potent established inhibitors (Brivanib, Sunitinib, Pazopanib, etc.) were gleaned against all the VEGFR targets towards cervical cancer. Several experimental studies utilizing conventional medicinal chemistry techniques have been carried out by researchers on established compounds such as Pazopanib, Brivanib, and Sunitinib for cervical cancer. According to their research, these medications have a high prevalence of side effects, which has led to a decrease in the use of these compounds^18–23^. The process of finding and creating novel pharmaceuticals can be speed up and improved with the help of Artificial Intelligence (AI), especially Machine Learning and Deep Learning, which are subcategories of AI. Though it is still in its early stages, artificial intelligence is already having a profound impact on the process of finding and developing novel targeted anti-cancer therapeutics. By utilizing Deep learning and Machine learning, scientists have discovered novel compounds that can treat various human diseases like cancer^24–30^. Machine learning-based drug discoveries that target all of the VEGFRs has not been extensively studied so far. Therefore the current investigation aims to leverage Machine Learning, Deep Learning, Molecular Docking, Molecular Dynamics Simulation and ADMET studies to identify a novel compound having high affinity, and less toxicity targeting VEGFR1, VEGFR2, and VEGFR3 for the treatment of cervical cancer.

## Methodology

### Established inhibitors of VEGFR

**Figure 2:**
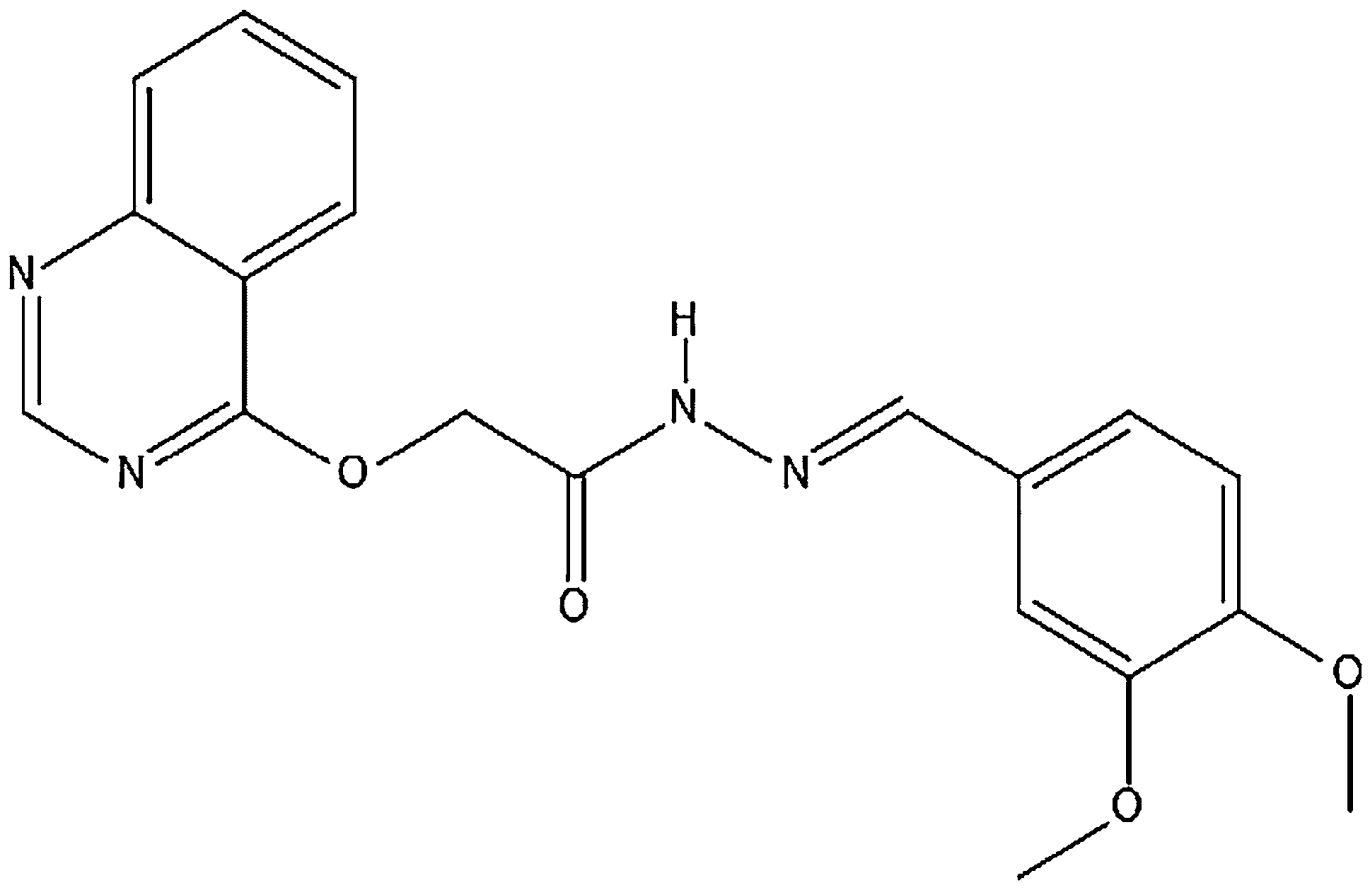
(E)-N′-(3,4-dimethoxybenzylidene)-2-(quinazolin-4-yloxy) acetohydrazide.

Supplementary Table 1 has provided a comprehensive overview of the chemical characteristics of validated inhibitors targeting VEGFR proteins, along with their selectivity towards the respective VEGFR isoform^31–55^. This data was meticulously curated through literature mining and extensive surveys of published research publications. In Fig. 2, a structural representation of a benzyl quinone derivative has been illustrated, which was generated using Marvin sketch software^56^.

### Ligand and Protein preparation

The co-crystallized structures of the target proteins VEGFR1, VEGFR2, and VEGFR3 were retrieved from the PDB database^57,58^ with the corresponding PDB IDs: 3HNG^59^, 1Y6A^60^, and 4BSJ^61^. These structures were stored in the PDB file format and have been summarized in Table 1. The construction of the target binding sites commenced with the identification of suitable cavities, guided by criteria outlined in Table 1, including chain type, volume, and radius^62–65^. The molecular structures of all 26 established inhibitors were sourced from NCBI’s PubChem chemical database (refer to Supplementary Table 1)^18–23,31–55^. Additionally, using Marvin sketch software, the compound ‘(E)-N’-(3,4-dimethoxybenzylidene)-2-(quinazolin-4-yloxy) acetohydrazide,’ which was not available in the PubChem database, was meticulously depicted and saved in 3D SDF file format. Subsequently, all inhibitors in .sdf format underwent further refinement in both 2D and 3D using the Marvin-sketch program before being introduced into the docking assay^66–75^.

**Table 1:** VEGFR isoforms and their binding cavity details utilized in the present study.

Isoform	GENE-ID	RefnSeq	UniProt ID	PDB-ID	Chain Type	Radius (Å)	Binding Site Volume (Å^3^)
VEGFR-1	2321	NP_002010.2	P17948	3HNG	A	25	87.55
VEGFR-2	3791	NP_002244.1	P35968	1Y6A	A	25	22.01
VEGFR-3	2324	NP_891555.2	P35916	4BSJ	A	25	183.8

### Molecular docking

The Molegro Virtual Docker software, serving as the docking platform, utilized high-scoring functions including Piece-wise Linear Potential (PLP), heuristic search functions, and the MolDock scoring function for energy reduction and compound scoring^76–82^. In the docking process, key parameters included a maximum population size of 50, a maximum iteration of 1,500, and a grid resolution of 0.3^83–86^. All known ligands were systematically docked with the prepared target cavities^87–89^. Results were obtained based on parameters like ligand evaluation, internal electrostatic score (ES), internal hydrogen bond, internal angles, sp2-sp2 torsion, and energy minimization. The post-docking process involved hydrogen bond optimization and energy minimization using MolDock Simplex Evolution, with a maximum of 300 steps and a neighbour distance factor of 1.00. The final step included energy minimization of the ligand-receptor complex via Nelder Mead Simplex Minimization^90–93^. The resulting output file was saved in the designated directory as an .xls file. The re-rank score emerged as the primary parameter for prioritizing compounds, guiding the selection of the top 10 hits for each VEGFR receptor. Machine learning-based compound models were subsequently generated based on the compound with the lowest re-rank score^29,30^.

### Deep learning-based de-novo designing of novel compounds

**Figure 3:**
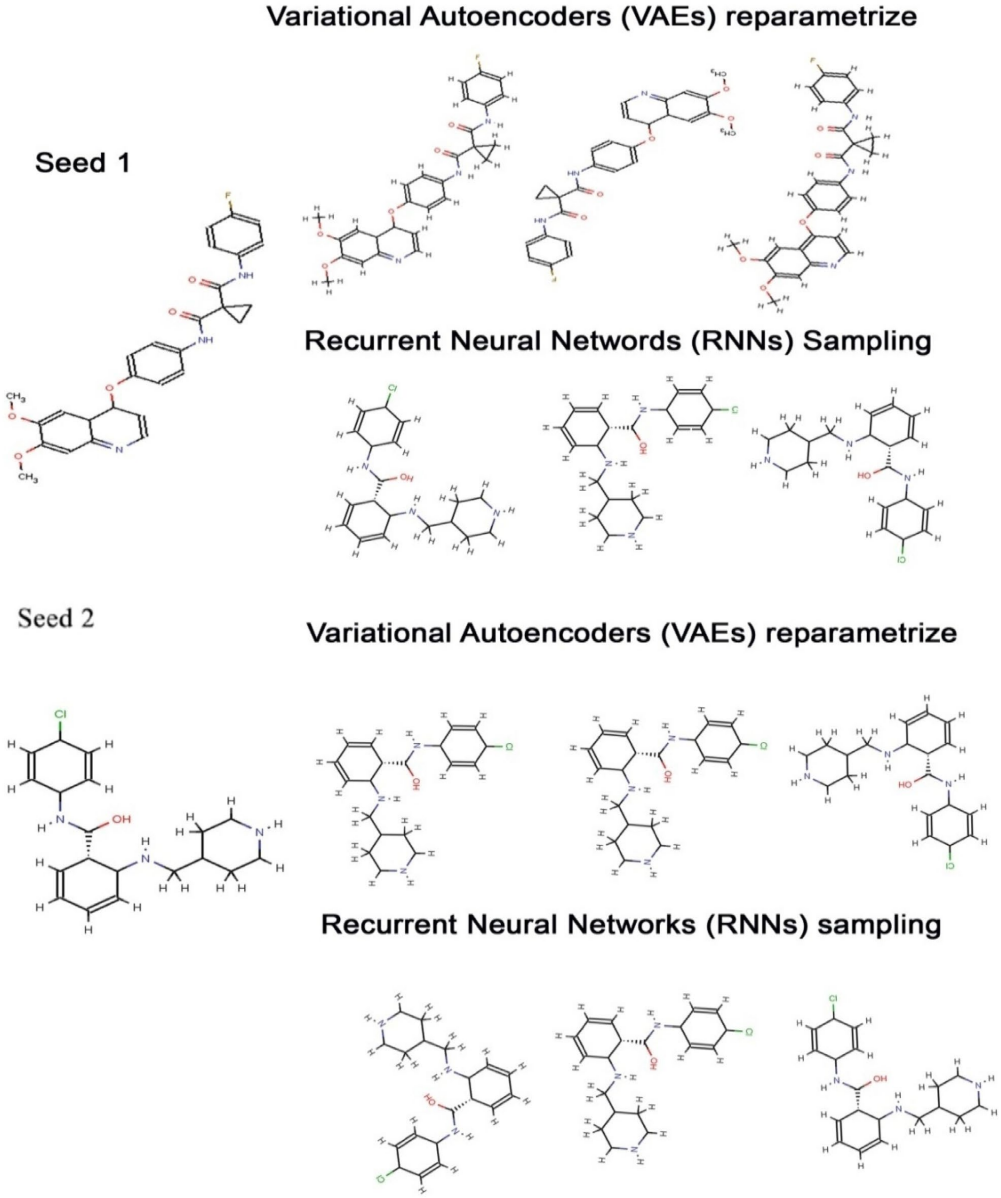
The De-novo shape-based compounds generated for VEGFR1 by VAE and RNN sampling using seed 1 and seed 2 as reference molecules.

Shape-based compounds targeting VEGFR1, VEGFR2, and VEGFR3 were constructed using deep learning algorithms^29,30^. The chemical structures were developed using SMILES strings and corresponding chemical spatial data, and Convolutional Neural Networks (CNN) were employed for encoding structural and shape variations^94–96^. Compounds were trained based on H-bond donor, H-bond Acceptor, Molecular weight, and LogP values, adhering to Lipinski’s Rule of Five^97^. The PubChem database facilitated the extraction of compounds for constructing training and test datasets. The ligand extracted from the complex structure of each targets (VEGFR1, VEGFR2 and VEGFR3) and the best compound obtained from molecular docking of each targets (VEGFR1, VEGFR2 and VEGFR3) were considered as seed molecules for Deep Learning. Seed compounds, N-(4-Chlorophenyl)-2-((pyridin-4-ylmethyl)amino)benzamide (from the 3D structure of VEGFR1—PDB ID: 3HNG) and PubChem ID: 25102847 served as seed 1 and seed 2, respectively, for generating shape-based models. Similarly, for VEGFR2 (PDB ID: 1Y6A), the compound 2-anilino-5-aryl-oxazole inhibitor (from the co-crystallized structure) and PubChem ID: 369976 were chosen as seed 1 and seed 2. For VEGFR3 (PDB ID: 4BSJ), AXITINIB (AG-013736 or (N-Methyl-2-(3-((E)-2-pyridin-2-yl- vinyl)-1H-indazol-6-ylsulfanyl)-benzamide) and PubChem ID: 208908 were considered as seed 1 and seed 2, respectively (Fig. 3). Molecular docking results guided the selection of effectively established compounds for seed compounds, contributing to the generation of shape-based models^29,30^.

**Figure 4:**
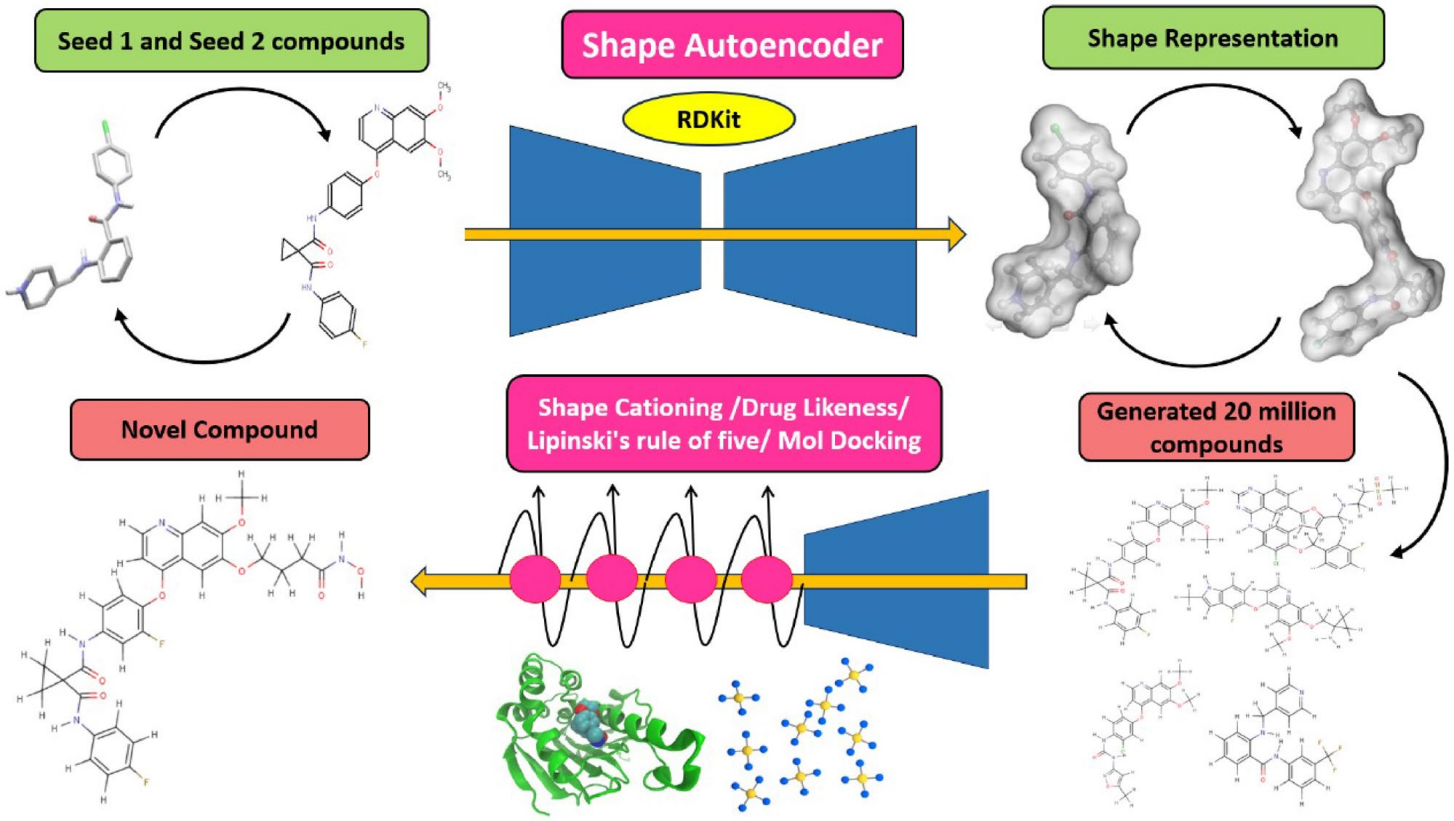
Workflow used to generate shape- based features for de-novo designing of novel compound.

In Python 3.10, the RDKit^98^ Machine Learning module was employed to create shape-based 3D conformers for individual compounds derived from the CNN-based shape variation autoencoder. Molecular mechanics force fields (MMFF94s) were then applied to the models using Python 3.10. To compute the Van der Waals radius of each compound, the HTMD miniconda module was utilized, by applying the relevant formulae^98^:

$${\text{n}}\left( {\text{r}} \right) \, = { 1 }{-}{\text{ exp}}\left[ { - \, \left( {{\text{r}}_{{{\text{vdw}}}} /{\text{ r}}} \right)^{{{12}}} } \right]$$

where **r**_**vdw**_, represent the particular Van der Waals radius of each atom of the compounds. The total binary cross entropies present in the Conditional Variational Autoencoder (CVAE) in the Autoencoder was calculated using the Kullback–Leibler divergence algorithm^98^. Canonical SMILES of each compound were used as input for training compounds^99,100^.

$$\mathcal{L}_{{{\text{VAE}}}} = - |\frac{1}{N}\mathop \sum \limits_{j = 1}^{{24^{3} \times 5}} y_{{\text{j}}} {\text{log }}\left( {{\text{p}}_{{\text{j}}} } \right) \, + \mathcal{L}_{{{\text{KL}}}}$$

where pi ∈ P^243×5^andy_j_ ∈ Y^243×5^ indicated voxel arrays and the model-generated ground-truth respectively, and £_KL_ for sample j was defined as^101,102^:

$$\mathcal{L}^{i}_{KL} = - \frac{1}{2}\mathop \sum \limits_{j = 1}^{j} [1 - \mu_{j}^{2} - \sigma_{j}^{2} + {\text{log}}(\sigma_{j}^{2} )]$$

where σ and μ were the outputs for the variational autoencoder (VAE) encoder and J represented the dimensionality of the latent vector. The captioning network minimized multiclass log lossas illustrated below^103,104^:

$$\mathcal{L}_{{{\text{caption}}}} = - \frac{1}{N}\mathop \sum \limits_{i = 1}^{N} \mathop \sum \limits_{j = 1}^{M} y_{{{\text{ij}}}} {\text{log }}\left( {p_{{{\text{ij}}}} } \right)$$

where "N" denoted the number of different samples in a batch and "M" represented the length of the protein sequence of the VEGFR gene. The training was carried out in batches using 410 trained samples. Variational Autoencoder (VAE) and recurrent neural networks (RNN) sampling were utilized for constructing fathomable de-novo shape-based structures concerning seed 2 chemicals (Fig. 4)^104–108^.To ensure the convergence of the captioning network, the shape-based Variational Autoencoder (VAE) and shape captioning structures underwent extensive training. This process yielded 20 million, 15 million, and 8 million compounds targeted towards VEGFR1, VEGFR2, and VEGFR3, respectively. The learning rate of the captioning network was halved after every 25,000 iterations. Subsequently, the top 10 chemically tailored compounds, containing valuable information, were selected for the simulation investigation through molecular docking and molecular dynamics [Supplementary Data 1]. The names and PubChem IDs of these top 10 compounds were determined by conducting a structure-based similarity search against the PubChem database.

### Generation of novel compounds

To formulate novel compounds, the generated chemical models underwent a comprehensive analysis using two sets of trained compound libraries: Recurrent Neural Networks and autoencoders reparameterizations^102,109–112^. From the pools of 20 million, 15 million, and 8 million shape-based generated compounds targeting VEGFR1, VEGFR2, and VEGFR3, respectively, the top 10 compounds were selected for the construction of novel compounds. These chosen 10 compounds underwent further assessment for drug-likeness and physicochemical characterization. Subsequently, leveraging the tunable homology within the seed compounds, 10 de-novo compounds were filtered and subjected for further molecular docking studies. The high-affinity compound derived from molecular docking of each target(VEGFR1, VEGFR2 and VEGFR3) were then further utilized for Molecular Dynamics Simulation (MDS)^113–116^.

### Molecular dynamics simulation

To analyse the stability and conformational dynamics of atoms in the compound, a molecular dynamics simulation was conducted on three isoforms of VEGFR in conjunction with the best-established compound and the top machine learning-based compound. The simulation duration was set to 100 ns using the Desmond module in Schrodinger, which operated based on the Newtonian dynamic equation^113–116^. Initially, the TIP3P model and OPLS5 force field were employed, and the system was neutralized with NaCl ions before being placed in a cubic simple point charge (SPC) water box. A two-step energy minimization at 50,000 ps was conducted until completion^117–119^. The simulation ran for 100 ns at an ambient temperature of 1.013 bar with a temperature set at 310 K^109,120^. To ensure high-quality results, the simulation was performed three times, generating around 1000 frames per simulation. The findings provided insights into thermodynamic aspects, including root mean square fluctuations (RMSF) and root mean square deviation (RMSD), unveiling structural and functional characteristics of the protein–ligand complex^109,120^.

### Pharmacophore studies

Amino acid residues participating in crucial hydrogen bond interactions were identified through the Molegro Virtual Docker. Various forms of chemical bond interactions, including hydrogen bonds, Vander Waals interactions, ionic bonds, covalent bonds, and water interactions within different pairs of receptor-inhibitor complexes, were comprehensively analyzed using the Discovery Studio 3.5 Visualize program^121–125^.

### ADMET studies and Boiled-egg plot

The AdmetSAR server was utilized for predicting the chemical profiles of small molecules with anticipated drug-like properties, encompassing aspects of absorption, digestion, metabolism, excretion, and toxicity^126^. These predictions offer insights into the behavior and efficacy of these molecules during clinical trials. ADMET studies play a crucial role in forecasting a drug’s potential success based on pharmacokinetic features, including bioactivity and toxicity^127–129^. For each VEGFR isomer, R Studio was employed to create a graphical interface for data visualization, facilitating a comparison between machine learning-built compounds and the best-established compounds. The Boiled Egg plot, a statistical visualization generated using the SwissADME web tool, was employed to assess the blood–brain barrier and gastrointestinal permeability^130^. This plot considered factors such as molecular weight, total polar surface area, MLogP value, gastrointestinal absorption, and blood–brain barrier characteristics^130^. The Boiled Egg plot was divided into three regions: yellow or yolk (indicating a higher probability of Blood–Brain Barrier penetration), white (indicating a good chance of intestinal absorption), and grey (indicating a low probability of intestinal absorption, characterizing a compound as non-absorptive and non-penetrative). After docking with each VEGFR isoform, based on a lower re-rank score for generating the Boiled Egg plot, two best-established compounds and two best machine learning-based compounds were identified. Each drug was separately evaluated for gastrointestinal absorption and blood–brain barrier characteristics^131–137^.

### Drug—drug comparison

The machine learning-based compound with the lowest re-rank score was sourced from the PubChem compound database. The protein structure was cleaned by reconfiguring all constraints, cavities, and ligands, resulting in a pristine protein structure saved in SDF format^138–140^. The best-established inhibitor was also cleaned and imported in .sdf format for a drug-drug comparison study. The comparison between the best-established molecule and the best machine learning-based compound with VEGFR isomers considered criteria such as relative hydrogen bond interactions, steric energy, and a lower re-rank score^141–144^.

## Results and discussion

### Protein and ligand preparation

**Figure 5:**
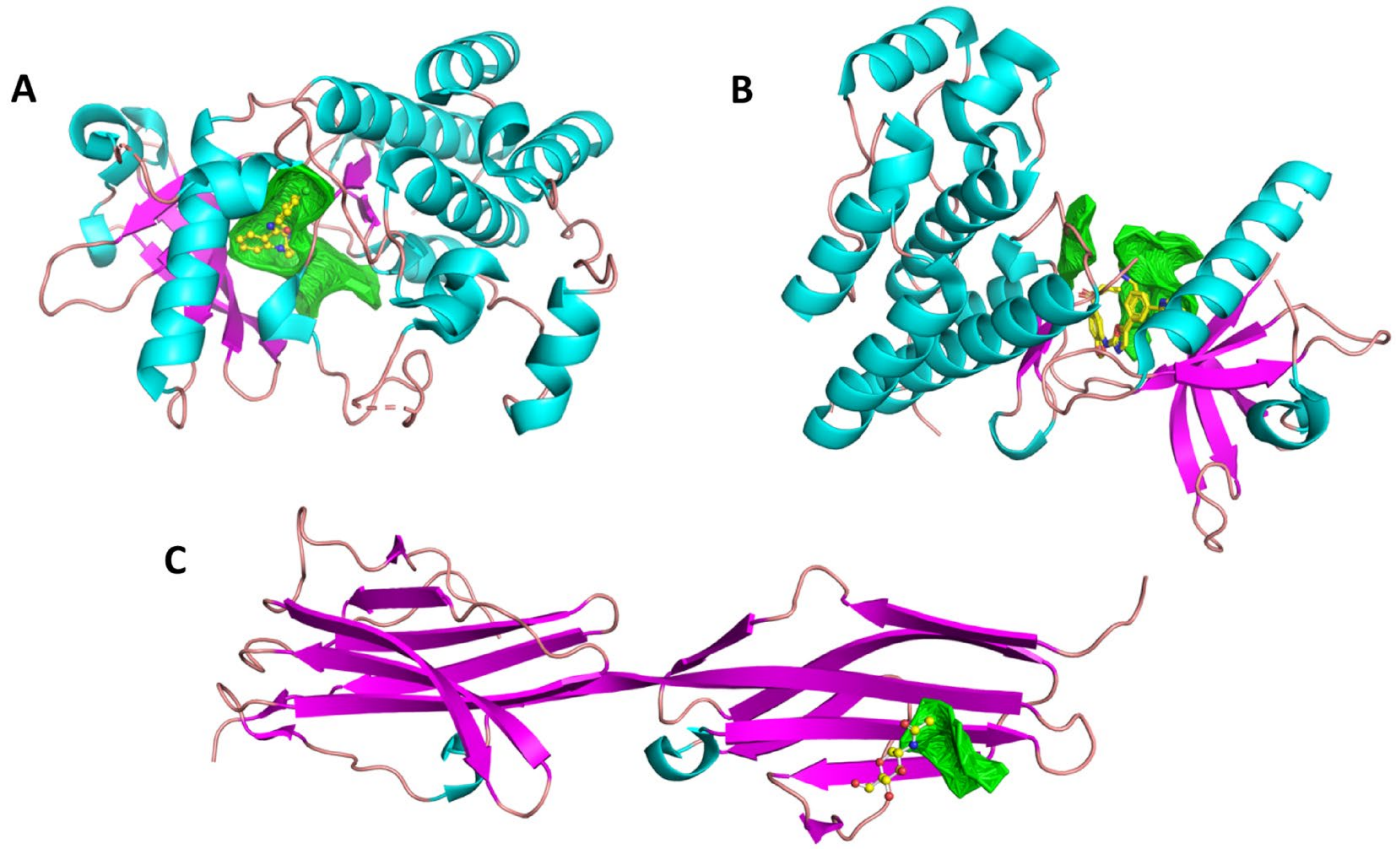
Representation of Protein 3D structure of VEGFR’s obtained from PDB—(**A**). VEGFR-1 (PDBID: 3HNG); (**B**). VEGFR-2 (PDBID: 1Y6A); (**C**). VEGFR-1 (PDBID: 4BSK); in which, each color represents as helix (Cyan), sheet (pink), loop (dark tints), and co-crystalized compound as yellow with elements color, as well as binding pocket in green.

VEGFR-1 (PDB ID: 3HNG) is a 15.2 kDa dual-chained heterotetrametric structure with 2299 atoms, 2374 bonds, 17 helices, and 7 beta-strands (Fig. 5A). VEGFR-2 (PDB ID: 1Y6A) is an 86.57 kDa double-chained protein, with about 2099 atoms, 2217 bonds, fourteen helices, and 7 beta strands (Fig. 5B). VEGFR-3 (PDB ID: 4BSJ) is a 13.59 kDapentachained protein, featuring 213 groups, 1673 atoms, 1751 chemical interactions, 2 helices, and 19 beta strands (Fig. 5C). For the molecular docking study, the first cavity of all receptors was selected, with VEGFR-1’s having a volume of 87.55, VEGFR-2’s with a volume of 22.016, and VEGFR-3’s with a volume of 183.8. The radius for all three receptors was maintained at 25 Å.

### Molecular docking studies

The Molegro Virtual Software’s Docking Wizard loaded all inhibitors into the created target cavity for molecular docking. The top 10 compounds with the lowest re-rank and MolDock Score, along with their molecular weights, were extracted and presented in tabular format for all three VEGFR isoforms. Notably, Compound Cabozantinib (22) with PubChem ID: 25102847 against the VEGFR-1 receptor (Table 2), Compound TNP-470(23–24) with PubChem ID: 36997 against the VEGFR-2 receptor (Table 3), and Lapatinib (25) with PubChem ID: 208908 against the VEGFR-3 receptor (Table 4) were selected for the construction of Machine Learning Models.

**Table 2:** Molecular docking: established inhibitors against VEGFR-1.

PubChem ID	Name	MolDock score (KJ/mol)	Re-rank score (KJ/mol)	MW (g/mol)
[00] 25102847	Cabozantinib	− 155.779	− 112.901	501.506
[01] 25102847	Cabozantinib	− 141.584	− 107.22	501.506
[04] 208908	Lapatinib	− 133.312	− 103.862	581.058
[01] 10297043	AEE-788	− 139.967	− 102.39	440.583
[01] 208908	Lapatinib	− 136.122	− 101.227	581.058
[01] 11234052	Brivanib	− 128.031	− 100.245	370.378
[00] 6450551	Axitinib	− 122.085	− 99.9181	386.47
[02] 9933475	Cediranib	− 130.775	− 98.8826	450.505
[04] 10297043	AEE-788	− 128.374	− 98.5575	440.583
[03] 25017411	Anlotinib	− 126.322	− 98.0086	407.437
[00] 442126	Decursin	− 122.708	− 97.7696	328.359

**Table 3:** Molecular docking: established inhibitors against VEGFR-2.

PubChem ID	Name	MolDock score (KJ/mol)	Re-rank score (KJ/mol)	MW (g/mol)
[00] 369976	Tnp-470	− 116.686	− 88.4552	401.882
[00] 208908	Lapatinib	− 131.941	− 88.4185	581.058
[01] 369976	Tnp-470	− 111.987	− 87.8381	401.882
[02] 369976	Tnp-470	− 114.702	− 87.0651	401.882
[01] 25017411	Anlotinib	− 114.843	− 85.8629	407.437
[00] 25102847	Cabozantinib	− 115.648	− 85.6008	501.506
[01] 208908	Lapatinib	− 137.694	− 85.1272	581.058
[03] 25102847	Cabozantinib	− 106.648	− 84.6492	501.506
[04] 25017411	Anlotinib	− 119.836	− 83.8888	407.437
[01] 11234052	Brivanib	− 116.095	− 83.869	370.378
[01] 25102847	Cabozantinib	− 111.291	− 81.7752	501.506

**Table 4:** Molecular docking: Established inhibitors against VEGFR-3.

PubChem ID	Name	MolDock score (KJ/mol)	Re-rank score (KJ/mol)	MW (g/mol)
[02] 208908	Lapatinib	− 158.144	− 118.195	581.058
[00] 9911830	Tivozanib	− 144.231	− 113.369	454.863
[03] 208908	Lapatinib	− 152.213	− 109.955	581.058
[01] 25102847	Cabozantinib	− 133.435	− 108.037	501.506
[01] 17536	Oxypeucedanin hydrate	− 134.001	− 106.623	304.295
[00] 6398883	AAL-993	− 129.671	− 106.112	371.356
[01] 10297043	AEE-788	− 124.179	− 105.301	440.583
[04] 208908	Lapatinib	− 148.129	− 104.772	581.058
[04] 5329102	Sunitinib	− 136.513	− 103.711	398.474
[02] 6398883	AAL-993	− 121.182	− 101.047	371.356
[02] 25017411	Anlotinib	− 129.917	− 100.759	407.437

### Molecular docking of top 10 compounds obtained from machine learning

**Figure 6:**
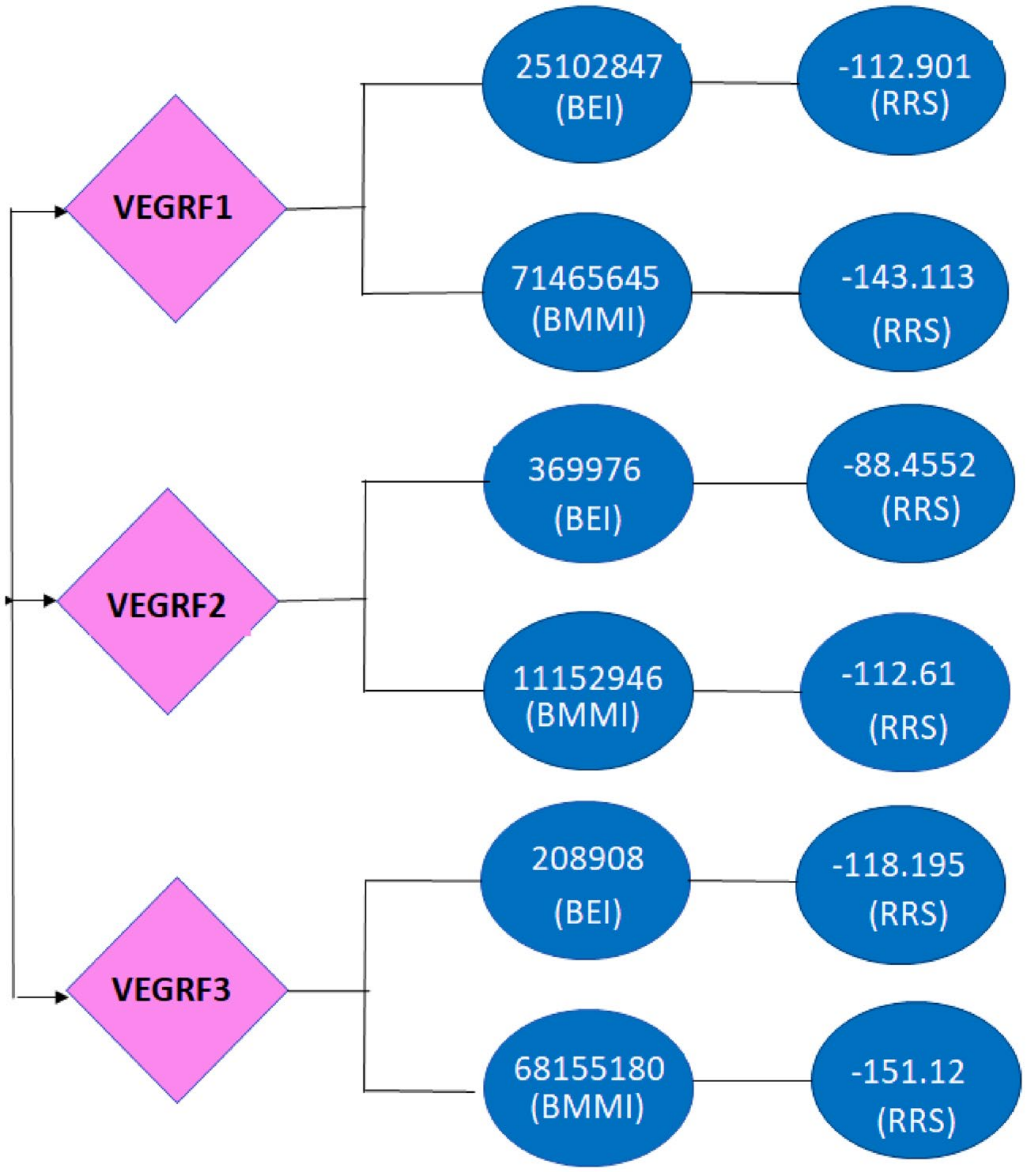
Re-rank Score Comparison between Established versus Machine Learning (ML) model compounds. BEI = Best established inhibitor; BMLI = Best ML model inhibitor; RRS = Re-rank score.

The top 10 compounds were chosen for Molecular Docking from a pool of 43 million compounds generated by Machine Learning models, following filtration based on drug-likeness and physicochemical characteristics. With the lowest re-rank scores, compounds 71465645 (Table 5), 11152946 (Table 6), and 1115294 (Table 7) emerged as the top candidates. Compound 71465645 (26) exhibits 4 Hydrogen Bond Donors (HBD), 10 Hydrogen Bond Acceptors (HBA), and a Topological Polar Surface Area of 148.2 in a 606.6 Da compound. Compound 11152946 (27), a 371.9 Da compound, features an HBD: HBA count of 1:5 and a Topological Polar Surface Area of 80.52. Compound 68155180 (28) possesses a Topological Polar Surface Area of 127.2, a molecular weight of 596.1 Da, along with three HBD and ten HBA. The re-rank scores for these three compounds are lower than the best-established inhibitors for the targeted VEGFR isoforms, suggesting their potential as superior drug candidates (Fig. 6). To validate this hypothesis, a drug-drug comparison analysis was conducted.

**Table 5:** VEGFR-1: Molecular Docking of Top 10 compounds obtained from Machine Learning.

Compound ID	Ligand	MolDock score (KJ/mol)	Re-rank score (KJ/mol)	MW (g/mol)
[00] 71465645	71465645	− 172.118	− 143.113	606.573
[00] 42642645	42642645	− 168.825	− 128.832	632.700
[00] 71576419	71576419	− 164.778	− 126.609	588.600
[00] 11594543	11594543	− 162.423	− 125.364	296.320
[00] 56604907	56604907	− 156.805	− 125.303	528.500
[00] 11317348	11317348	− 161.427	− 122.725	469.400
[02] 86269669	86269669	− 154.597	− 122.698	474.400
[01] 86269462	86269462	− 156.966	− 122.625	597.600
[02] 78325042	78325042	− 154.085	− 122.197	517.500
[01] 57810164	57810164	− 155.67	− 121.197	408.400
[01] 46189868	46189868	− 149.907	− 120.952	828.600

**Table 6:** VEGFR-2: Molecular Docking of Top 10 compounds obtained from Machine Learning.

Compound ID	Ligand	MolDock score (KJ/mol)	Re-rank score (KJ/mol)	MW
[01] 11152946	11152946	− 115.968	− 112.61	371.856
[00] 125367	125367	− 117.208	− 101.185	325.400
[02] 9930932	9930932	− 113.044	− 101.011	401.900
[00] 5326425	5326425	− 117.093	− 100.301	403.900
[00] 60791	60791	− 117.015	− 99.101	401.900
[04] 45482966	45482966	− 110.481	− 99.100	381.500
[00] 45482954	45482954	− 116.648	− 98.562	410.500
[02] 44385040	44385040	− 111.353	− 96.231	387.900
[01] 44343813	44343813	− 115.426	− 96.117	401.900
[00] 14942882	14942882	− 113.483	− 95.917	401.900
[01] 163806427	163806427	− 110.475	− 93.564	483.800
[00] 163508288	163508288	− 104.696	− 81.739	508.400

**Table 7:** VEGFR-3: Molecular Docking of Top 10 compounds obtained from Machine Learning.

Name	Ligand	MolDockScore(KJ/mol)	Re-rank Score(KJ/mol)	MW
[00] 68155180	68155180	− 198.138	− 151.12	596.100
[00] 9941095	9941095	− 181.263	− 148.112	925.500
[00] 91798457	91798457	− 279.923	− 146.013	1868.900
[02] 11679357	11679357	− 278.288	− 143.102	753.300
[00] 208909	208909	− 241.055	− 142.001	915.400
[00] 16219404	16219404	− 189.778	− 141.500	671.000
[01] 139061731	139061731	− 99.451	− 140.911	925.500
[00] 11181296	11181296	− 112.116	− 138.186	473.900
[01] 5329480	5329480	− 218.191	− 136.109	598.100
[00] 118753054	118753054	− 171.121	− 132.876	597.100

### Molecular dynamics simulation

**Figure 7:**
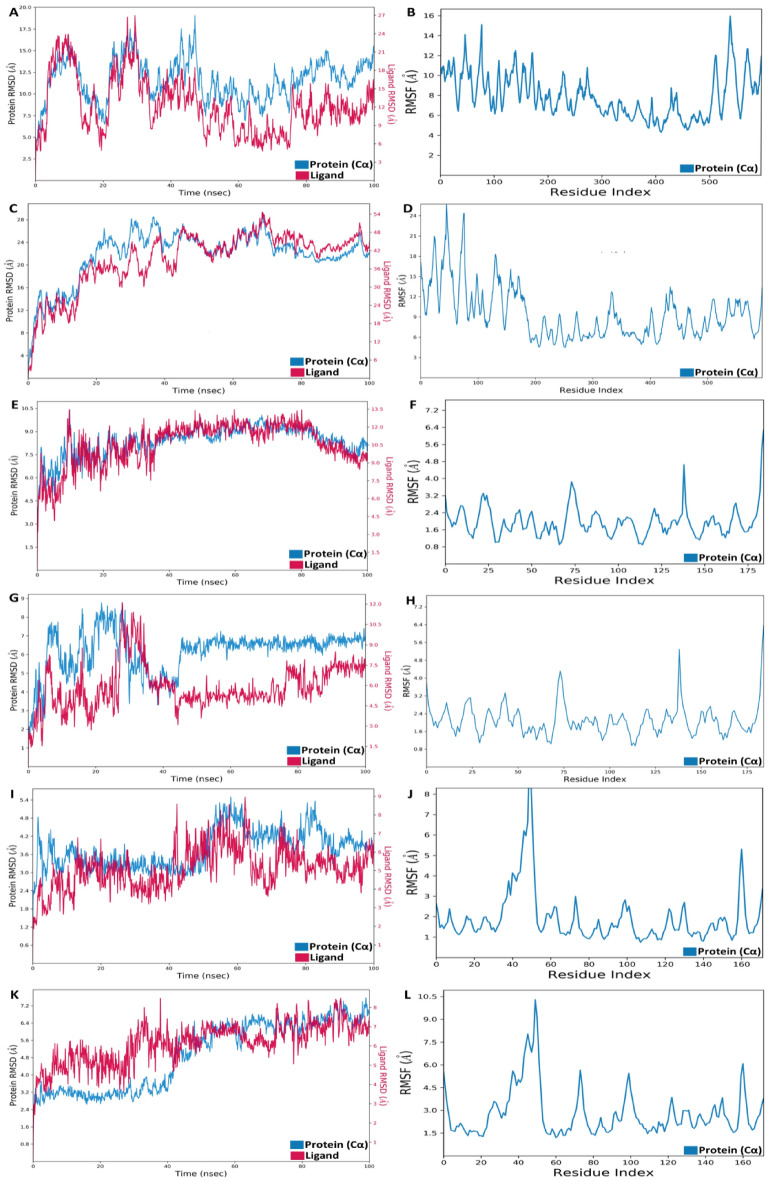
Molecular dynamics Root Mean Square Deviation (RMSD) and Root Mean Square Fluctuation (RMSF) analyses of VEGFR-1, VEGFR-2, and VEGFR-3 complexes with both the best-established compounds and Machine Learning-based generated compounds. The VEGFR-1 complexes are represented with PubChem ID: 25102847 for the best-established compound (**A** & **B**) and PubChem ID: 71465645 for the best ML Model compound (**C** & **D**). Similarly, the VEGFR-2 complexes are illustrated with PubChem ID: 369976 for the best-established compound (**E** & **F**) and PubChem ID: 11152946 for the best ML Model compound (**G** & **H**). Lastly, the VEGFR-3 complexes are depicted with PubChem ID: 208908 for the best-established compound (**I** & **J**) and PubChem ID: 68155180 for the best ML Model compound (**K** & **L**).

Following the docking of the protein–ligand complex, a dynamic simulation was conducted over 100 ns, assessing the complex’s thermodynamic stability through the examination of Root Mean Square Fluctuations (RMSF) and Root Mean Square Deviation (RMSD) values. The RMSD data offered insights into the intermolecular distances between the protein and ligand molecules, providing a measure of the overall stability of the complex throughout the simulation. Meanwhile, RMSF values illustrated the fluctuations of individual protein residues during the entire 100 ns simulation, shedding light on the dynamic behavior of the complex at the residue level. In the VEGFR-1:25102847 complex, as depicted in Fig. 7A & B, the Root Mean Square Deviation (RMSD) value for the protein exhibited a range of 6.5–17 Å, with an average RMSD of 13 Å in the last 50 ns of the simulation in the presence of the ligand. Meanwhile, the flexibility of residues within the protein showed a fluctuation range of 4.2–15.6 Å. Despite occasional conformational changes, the RMSD value for the VEGFR-1:71465645 complex was higher, albeit with less fluctuation. The most substantial fluctuation observed was 28 Å for the protein and 54 Å for the ligand, likely attributed to highly flexible residues within the range of 5–26 Å, as illustrated in Fig. 7C & D.In contrast, the RMSD values in the VEGFR-2:369976 complex remained relatively stable in the presence of the ligand, fluctuating within the range of 4.5–10 Å with an average of 9 Å for the ligand, while the protein exhibited fluctuations within the range of 7.5–12 Å with an average of 12 Å over the 100-ns simulation. Notably, the residual flexibility in this complex displayed the lowest trend, ranging from 0.9 to 5.9 Å, as illustrated in Fig. 7E & F. Furthermore, the RMSD values of both the protein and the ligand in the VEGFR-2:11152946 complex demonstrated high fluctuations during the initial 45 ns, ranging from 2 to 8.8 Å for the protein and 2–12 Å for the ligand. However, in the last 55 ns, the average RMSD value for the protein stabilized at 6.5 Å, possibly attributed to less flexible residues within the range of 0.8 to 5.6 Å, as depicted in Fig. 7G & H. Furthermore, the protein RMSD value for the VEGFR-3:208908 complexes exhibited fluctuations in the range of 2.4–7.2 Å, averaging 3.6 Å for the initial 50 s and 4.8 Å for the subsequent fluctuating period of 60 ns. These variations could be attributed to occasional peak fluctuations in the residues, as illustrated within the range of 1.5–9.0 Å, as shown in Fig. 7I & J. In contrast, the VEGFR-3:68155180 complex demonstrated RMSD stability within 3.3 Å for the first 40 ns, followed by an increase, indicating flexibility in amino-acid residues that escalated up to 10 Å, as depicted in Fig. 7K & L.

#### Simulated complex interaction profile

The interaction between the protein and ligand was scrutinized through a histogram and heatmap, illustrating the interaction fraction and types of interactions, including hydrogen bonding, hydrophobic bonds, ionic bonds, and water bridges. In the VEGFR-1:25102847 complex, numerous residues engage in hydrophobic interactions, with one demonstrating ionic bonding. Notably, residues such as TRY 105 and VAL 67 exhibit robust interactions with the ligand, forming hydrophobic bonds with the ligand’s benzene ring, amine, and keto group [Supplementary Data II Fig. I]. These interactions are attributed to the hydrophobic nature of the compound. In contrast, the VEGFR-1:71465645 complex showcases a prevalence of hydrogen bonding over hydrophobic bonding. Nevertheless, residues such as TRY 105 and VAL 67 continue to play a significant role, forming strong hydrogen bonds with the ligand and emphasizing the robust interaction between the ligand and the protein molecule [Supplementary Data II Fig. II].

In the MD simulation analysis of the best-established molecule, (PubChem CID: 369976) with VEGFR-2, hydrogen bond interactions emerged as the predominant chemical interactions. SER 153 and ILE 154, involved in hydrogen bonding and water bridge formation, were the key contributors, donating their side chains to the ligand’s keto-oxygen. Additionally, LEU 157 made substantial hydrophobic interactions. Specifically, SER 153 formed two bonds, comprising one hydrogen bond and another with a water molecule contact, with interaction occupancies of 54 percent and 45 percent, respectively. ILE 154 interfaced with one of SER 153’s interacting oxygen with 46 percent occupancy. On average, five amino acid residues engaged with the ligand, illustrating the multifaceted nature of the interactions [Supplementary Data II Fig. III]. Conversely, the intermolecular interaction between the VEGFR-2 receptor complexed with its best ML Model compound (PubChem CID: 11152946) was characterized as primarily hydrophobic, accompanied by a substantial number of hydrogen bonding events facilitated by water bridges. Notably, TYR 214 and ILE 212 formed a robust water bridge, while interacting hydrophobically with TRP 179 and LEU 157. Hydrogen bonding interactions involved CYS 150 and LEU 151. The interaction occupancy of ILE 212 with the water molecule was notable at 36 percent. On average, the residue made six interactions with the lead compound, highlighting the intricate interplay of hydrophobic and hydrogen bonding interactions in this complex [Supplementary Data II Fig. IV].

In the MD simulations of VEGFR-3 with the best-established compound (PubChem CID: 208908), TYR 109 emerged as a key regulator of protein–ligand interaction, prominently involved in hydrophobic contacts and water bridge formation. Other amino acid residues with notable interaction frequencies included ALA 64, contributing to hydrogen bonds, water bridges, and hydrophobic interactions; ILE 49 and LEU 96, involved in hydrophobic interactions; LEU 97, engaged in hydrogen bonds; and PRO 91, facilitating hydrogen bonds and water bridges in the latter half of the simulation period. On average, an amino acid residue made approximately 5 contacts with the ligand, as illustrated in Supplementary Data II Fig. V. In comparison, the best ML Model compound for VEGFR-3:68155180 exhibited a 1.4-fold increase in interaction frequency with TYR 109 compared to the best-established compound (208908). Additionally, the ML Model compound demonstrated heightened interactions with TYR 109, TRP 61, ASN 104, TRP 59, LEU 96, LEU 47, and VAL 130. TYR 109, through side chain donation, exhibited a 43 percent interaction occupancy with the ligand’s molecule, while ASN 104 and TRP 59 demonstrated 40 percent and 63 percent interaction occupancy, respectively, primarily through the creation of robust hydrophobic interactions with the ligand’s benzene ring. Moreover, the average number of amino acid residues coming into contact with the ML Model compound increased to eight [as shown in Supplementary Data II Fig. VI].

#### Examination of ligand properties during simulation

The characteristics of ligands in association with VEGFR compounds, including both the best-established and ML model compounds, were examined through a comprehensive analysis of Molecular Surface Area (MolSA), equivalent to the Van der Waals surface area; Solvent Accessible Surface Area (SASA), representing the surface area accessible to water molecules; and Polar Surface Area (PSA), denoting the surface area accessible for polar bond interactions. Additionally, parameters such as Root Mean Square Deviation (RMSD) and Radius of Gyration (rGyr) were evaluated to gauge the atomic moment of the compounds.

The RMSD values for the VEGFR-1: 25102847 complexes exhibited a range of 1–3 Å until 30 ns, gradually stabilizing around an average value of approximately 2.7 Å. In contrast, the rGyr graph indicated minimal variation, maintaining an average value of about 6.2 Å, ranging from 5.7 to 6.6 Å. Over the initial 30 ns, the molecule’s surface area displayed fluctuations, subsequently stabilizing around 464 Å^2^. The average surface area exposed to solvent settled at 210 Å^2^, while the polar surface area fluctuated between 100 to 120 Å^2^, with a mean of 110 Å^2^ [Supplementary Data III Fig. A]. For the VEGFR-1:71465645 complex, the RMSD value was comparatively lower, averaging 1.4 Å, while the rGyr graph indicated a mean value of 6.5 Å. The molecular surface area ranged from 540 to 550 Å^2^, with a mean value of 530 Å^2^, providing increased interaction contact compared to the previous complex structure. Despite a polar surface area of about 225 Å^2^, the solvent surface area exhibited variability, ranging from 200 to 400 Å^2^ [Supplementary Data III Fig. B].

In contrast to the best-established complex VEGFR-2:369976, the ligand properties associated with the VEGFR-2 receptor exhibited a notable decrease in the best ML Model compound complex VEGFR-2:11152946 [Supplementary Data III Fig. C and Fig. D]. The VEGFR2:369976 complex displayed an average RMSD of 1.8 Å, which was higher than the 0.6 Å observed in the VEGFR-2:11152946 complex. For the VEGFR-2:369976 complex, the values for rGyr, MolSA, SASA, and PSA ranged from 4 to 4.75 Å, 360–384 Å^2^, and 180–360 Å^2^, respectively. Conversely, in the VEGFR-2:11152946 complex, these values were within the ranges of 3.6–4.4 Å, 315–360 Å^2^, and 100–130 Å^2^, respectively. Notably, there was a discernible fluctuation trend in all parameters in the VEGFR-2:11152946 complex, spanning from 30 ns to roughly 42 ns, attributed to protein–ligand interactions [Supplementary Data II Fig. IV].

Whereas, in the VEGFR-3: 208908 complexes, the RMSD value of the ligand ranged from 1.5 to 3 Å, with a mean of 2.4 Å. The moment of inertia was concentrated in the range of 5–7.2 Å, with a mean of 6.13 Å. The Molecular Surface Area (MolSA) was around 520 Å^2^, and the surface interface for solvent averaged between 300 and 400 Å^2^, while the polar surface area was approximately 127 Å^2^ [Supplementary Data III Fig. E]. Conversely, in the VEGFR3: 68155180 complexes, a larger RMSD value was observed, with the rGyr value ranging from 5.6 to 8 Å, stabilizing in the second half of the simulation time. The molecular surface area was approximately 530 Å^2^ larger than the previous complex, yet the accessible surface area showed reduced variability, averaging between 90 and 250 Å^2^. The polar surface area exhibited inconsistency across the simulation period, with an average of around 170–180 Å^2^ [Supplementary Data III Fig. F]. These dynamic assessments of ligand behavior in complex with VEGFR-3 provide valuable insights into the structural stability and interaction profiles of these complexes.

### Drug—drug comparative studies

The drug-drug comparison involves assessing the efficiency of an ML Model compound in comparison to an established compound for a specific inhibitor. This evaluation is based on the disparity in values across various parameters detailed in [Supplementary Table II]. Individual re-rank scores are assigned to key parameters such as Total Energy, Protein–Ligand Interactions, Steric (by PLP), and Torsional Strain, measured in kJ/mol. These scores play a crucial role in determining the stability of the interaction. For VEGFR-1, the ML Model compound consistently outperforms the best-established compound, as reflected in lower re-rank scores across all parameters. Notably, the Protein–Ligand interactions and Steric (by PLP) parameters exhibit more negative values for the best-established inhibitor of VEGFR-2, indicating a potentially superior interaction. In the case of VEGFR-3, the ML Model inhibitor demonstrates a higher Torsional Strain compared to the best-established compound. However, the Total Energy re-rank score is consistently more negative for the ML Model inhibitors across all three VEGFR receptors, suggesting enhanced efficiency. Overall, the ML Model compounds exhibit favorable re-rank scores, particularly in total Energy, indicating their potential as more efficient inhibitors across the VEGFR receptors.

### Pharmacophore interactive studies

**Figure 8:**
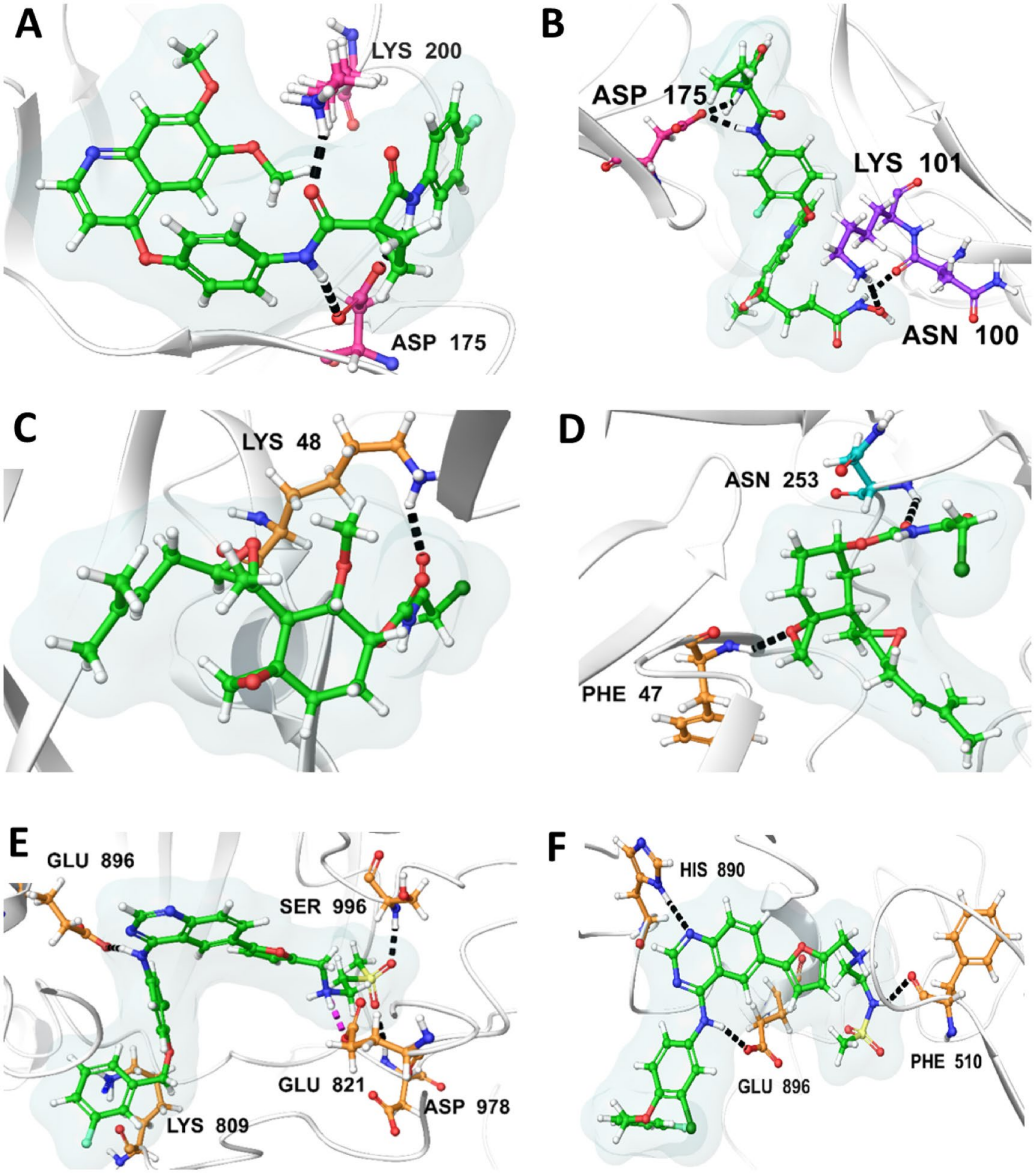
Illustrating the 3D interaction profiles of the most effective established and machine-learning (ML) model compounds against VEGFRs: (**A**) The most effective established compound PubChem ID: 25102847, shows H-bond interactions with VEGFR-1 [Pink—Y Chain Residues, Green—Ligand], (**B**) The most effective Machine Learning model compound PubChem ID: 71465645, shows H-bond interactions with VEGFR-1 [Pink—Y Chain & Purple—V Chain Residues, Green—Ligand]; (**C**) The most effective established compound PubChem ID: 369976, shows H-bond interactions with VEGFR-2 [Golden—A Chain Residues, Green—Ligand], (**D**) The most effective machine learning model compound PubChem ID: 11152946, shows H-bond interactions with VEGFR-2 [Golden—A Chain & Cyan—V Chain Residues, Green—Ligand]; (**E**) The most effective established compound PubChem CID: 208908, shows H-bond interactions with VEGFR-3 [Golden—A Chain Residues, Green—Ligand], (**F**) The most effective machine learning model compound PubChem CID: 68155180 shows H-bond interactions with VEGFR-3[Golden—A Chain Residues, Green—Ligand].

Hydrogen bond interactions play a crucial role in contributing to the stability of protein–ligand complexes. Examining the interactions in VEGFR-1, the best-established compound (ID: 25102847) forms two hydrogen bonds with Lys200 and Asp175 residues, as illustrated in (Fig. 8A). In contrast, the best ML model compound (ID: 71465645) establishes three hydrogen bonds with Asn100, Lys101, and Asp175, as depicted in (Fig. 8B). Moving to VEGFR-2, the best-established compound (ID: 369976) engages in a single hydrogen bond interaction with Lys48, as shown in (Fig. 8C). Conversely, the ML model compound (ID: 11152946) establishes two hydrogen bonds with Phe47 and Asn253, as illustrated in (Fig. 8D). In the case of VEGFR-3, the established inhibitor (ID: 208908) forms five hydrogen bonds with residues Lys809, Glu821, Glu896, Asp978, and Ser996, as seen in (Fig. 8E). On the other hand, the best ML model compound (ID: 68155180) engages in three hydrogen bond interactions with residues Phe510, His890, and Glu896, as depicted in (Fig. 8F).

Supplementary Data IV represents the surface image active site cleft of bound VEGFR Receptor with different inhibitors which are used to understand the chemical interactions happening between two protein entities in a complex. Compound ID 25102847 established 7 electrostatic interactions and 12 Van der Wall interactions [Supplementary Data IV Fig. I:A] while 71465645 established 11 electrostatic interactions and 13 Van der Wall [Supplementary Data IV Fig. I:B]. 369976 established 9 electrostatic interactions and 10 Van der Wall interactions [Supplementary Data IV Fig. I:C] while 11152946 established 7 electrostatic interactions and 11 Van der Wall [Supplementary Data IV Fig. I:D]. 208908 established 13 electrostatic interactions and 11 Van der Wall interactions [Supplementary Data IV Fig. I:E] while 68155180 established 11 electrostatic interactions and 15 van der Wall [Supplementary Data IV Fig. I:F].

### ADMET studies

**Figure 9:**
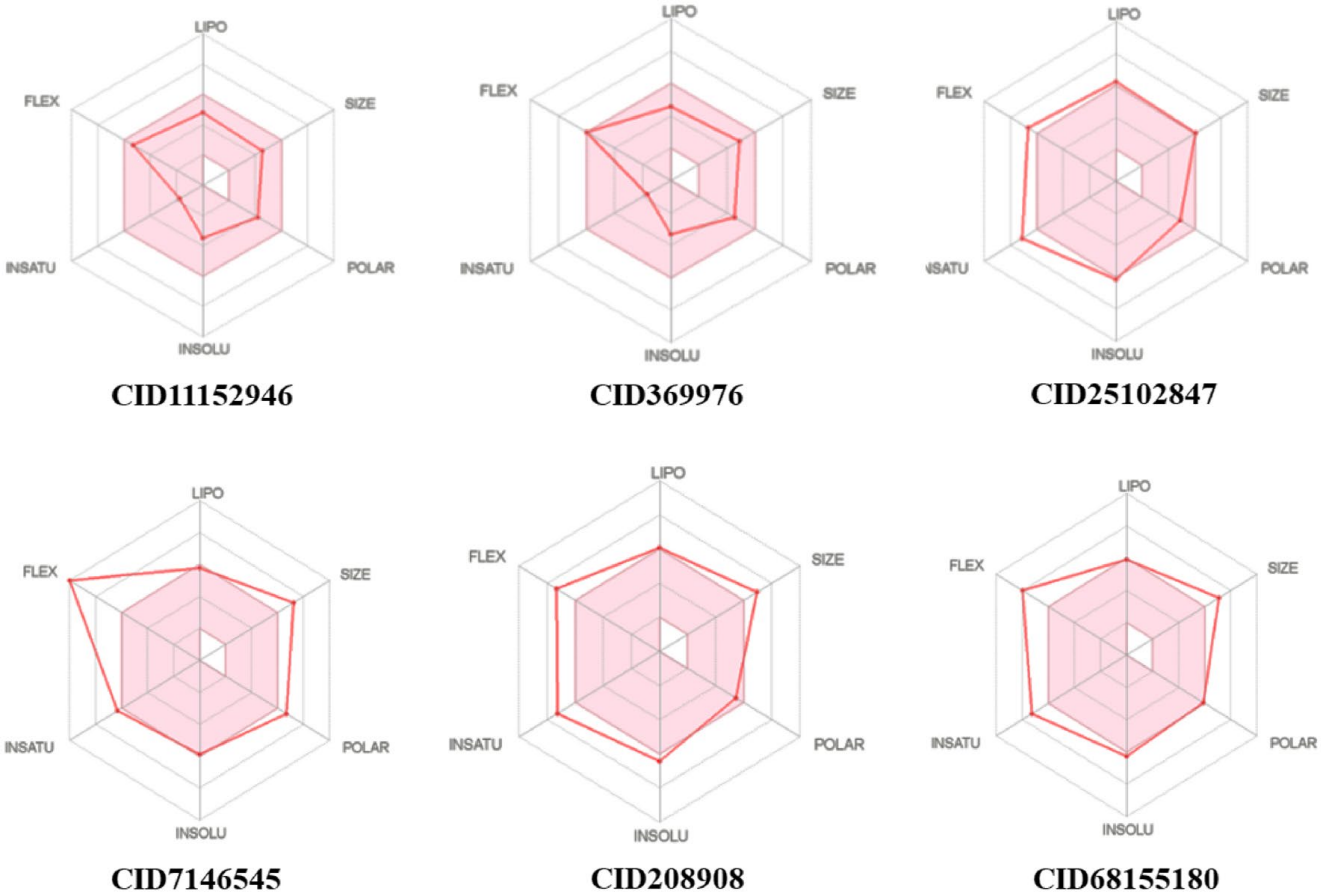
Bioavailability radar related to physicochemical properties of two of each best compound from established and machine learning compounddocked result.

ADMET analysis played a pivotal role in assessing the viability of the best machine learning model compound for its potential in blocking VEGFR receptors. This analysis, integral in drug development, serves to predict a medicine’s clinical success, thereby minimizing the likelihood of drug failure. Predictions encompassing carcinogenicity, toxicity, blood–brain barrier penetration, and human intestinal barrier permeation were crucial in evaluating the drug’s suitability for clinical trials, particularly for screening inhibitors of VEGFR 1, VEGFR2, and VEGFR 3, as detailed in Supplementary Table III. The bioavailability radar, generated by SwissADME software, provided a comprehensive overview of the best-established compounds and machine learning compounds inhibiting the target receptor proteins VEGFR 1, VEGFR2, and VEGFR 3 (Fig. 9). The pink region in the radar represents the optimal range of values. Notably, all compounds demonstrated the ability to penetrate the blood–brain barrier (BBB-) but not the small intestine (HIA +). Furthermore, negative results in carcinogenic and hazardous tests suggest their potential utility as VEGFR inhibitors. These findings collectively underscore the promising characteristics of the machine learning model compound in its journey towards clinical application.

**Figure 10:**
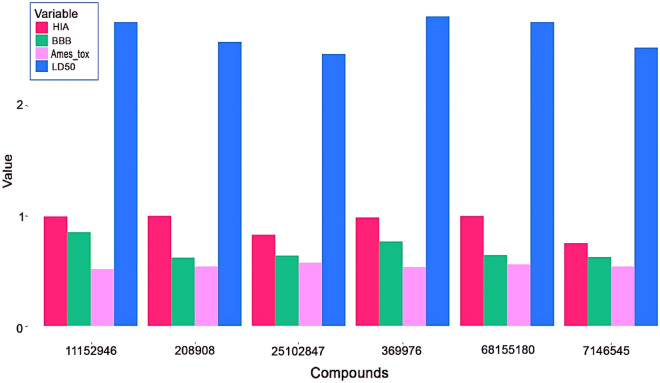
Comparative analysis of HIA, BBB, AMES toxicity, LD50 values of established compounds against machine learning compounds.

Figure 10 represents the outcomes of drug-likeness properties, physicochemical characteristics, and pharmacokinetics for both the top established compounds and machine learning model compounds. To facilitate the interpretation of parameters such as Human Intestinal Absorption (HIA), Blood–Brain Barrier (BBB) permeability, Ames’s toxicity, and Rat LD50, a barplot was generated using the ggplot2^145,146^ package in the R programming language^147^. This visual representation offers a concise and comparative overview of these critical properties for the established and machine learning model compounds, aiding in the assessment of their overall suitability and safety profiles for further consideration in drug development.

### Boiled EGG Plot analysis

**Figure 11:**
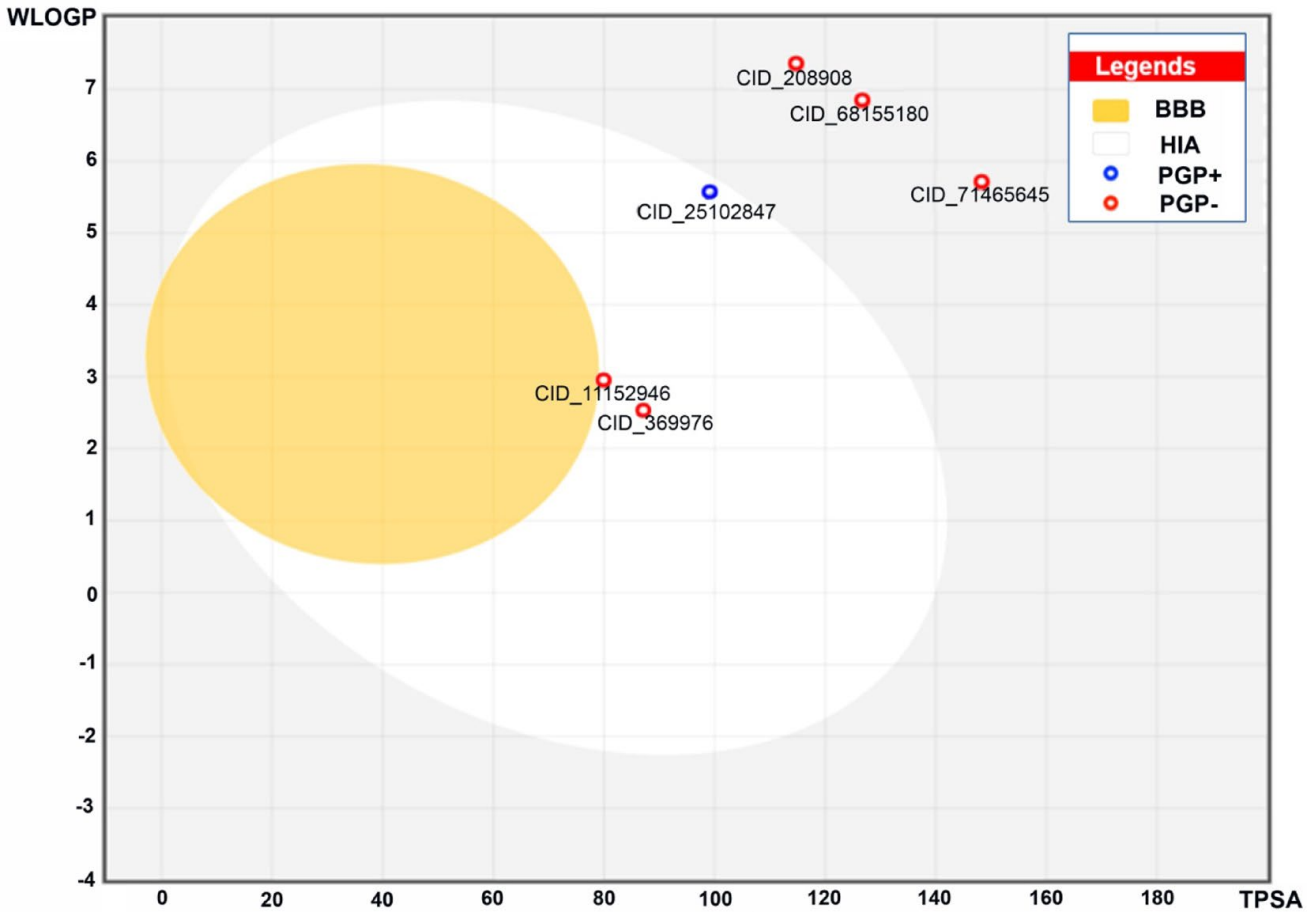
Boiled Egg Plot of six most effective machine learning compounds and established compounds.

The boiled egg plot is employed to assess the efficacy and capability of small compounds in traversing the gastrointestinal and blood–brain barriers, with these properties determined by the molecules’ polarity and lipophilicity, as outlined in Table 8. Physicochemical spaces are categorized into three regions: yellow, white, and grey. Molecules residing in the yellow area have the highest likelihood of crossing the blood–brain barrier, while the white region, encompassing compounds with PubChem IDs 11152946, 25102847, and 369976, signifies compounds capable of passing through the gastrointestinal barrier. Notably, compounds 208908, 68155180, and 71465645 are situated in the grey area, indicating diminished absorption across both the gastrointestinal and blood–brain barriers. Specifically, the VEGFR-2 receptor machine learning compound 11152946 exhibits a reduced Blood–Brain Barrier (BBB) crossing index and enhanced gastrointestinal perseverance. The grey region also implies reduced penetration of the gastrointestinal and blood–brain barriers for machine learning model compounds VEGFR-1:71465645 and VEGFR-3:68155180. VEGFR-1 and VEGFR-2 Bioavailability Equivalent Indices (BEI) fall within the white region, whereas VEGFR-3 BEI is positioned in the grey region (Fig. 11).

**Table 8:** Best established docked compounds and bestML compounds used for Boiled Egg plot.

PubChem ID	MW (g/mol)	TPSA(Å^2^)	MLOGP	GI absorption	BBB permeant
11152946	371.9	80.5	1.75	High	No
369976	401.9	89.7	1.18	High	No
25102847	501.5	98.8	2.68	High	No
71465645	606.6	148.11	2.46	Low	No
208908	581.1	115	3.44	Low	No
68155180	596.1	126.76	2.67	Low	No

## Discussion

The rising red queen race of AI and DL algorithms have emerged as chief parameters for combatting myriad neoplasms, especially in the drug designing, and discovery field. These AI models aid in random screening of various compounds, and predict bioactivities of known and unknown biological compounds. In the current investigation, a thorough literary survey was conducted in which 26 potent established inhibitors, such as Brivanib, Sunitinib, Pazopanib, etc. were gleaned against all the selected VEGFR (1–3) genes. These established inhibitors were then subjected to Molecular Docking studies, in which the best-established inhibitors depending upon re-rank score were gleaned; VEGFR-1—Cabozantinib (PubChem ID: 25102847), VEGFR-2—Tnp-470(PubChem ID: 369976), VEGFR-3—Lapatinib (PubChem ID: 208908). The data from the top hits of the Molecular Docking studies were then fed as training datasets and Deep Learning algorithms were employed to generate millions of chemically tailored compounds exhibiting profound chemical and novel characteristics.

The top selected novel compounds against VEGFR-1—PubChem ID: 71465645, VEGFR-2—PubChem ID: 11152946, and VEGFR-3 –PubChem ID: 68155180 were confirmed via Molecular Docking and Deep Learning algorithms. These novel compounds were further subjected to comparative analysis with the established inhibitors, testing their drug-likeness, physiochemical characterization, and Molecular Dynamic Simulation studies, which revealed that the Deep Learning generated novel compounds, exhibited augmented affinity scores, and better physiochemical characteristics than the best-established inhibitors. Moreover, pharmacophore and ADMET analysis also indoctrinated the lower toxicity, and higher vitality levels of the new drugs, suggesting their suitability as a prime therapeutic agent for treating cervical cancer.

Despite the numerous advantages of these DL and AI models, there are countable limitations that hinder the ability of these models to govern the absolute predictions, such as sensitivity to noisy data, over-fitting the training data sets, and limited training data availability which ultimately leads to challenges in its predictions and validations, etc. But with the advent of technology, numerous Machine Learning (ML), DeepLearning (DL), Artificial Intelligence (AI) models, and algorithms are being devised daily, to circumscribe these limitations and restrictions.

## Conclusion

Cervical cancer, often associated with human papillomavirus infection, disrupts the tyrosine-kinase receptor pathway and gene signaling cascade, impacting VEGFR-1, VEGFR-2, and VEGFR-3 receptor proteins. In our study, we identified 26 established inhibitors for VEGFR proteins through an extensive literature search. Subsequently, molecular docking of these inhibitors led to the discovery of a machine learning-derived compound with PubChem IDs: 71465645 (VEGFR-1), 11152946 (VEGFR-2), and 68155180 (VEGFR-3) exhibiting inhibitory effects on respective VEGF receptors. ADMET analysis and a boiled egg-plot were employed to assess drug similarity features, revealing substantial bioavailability for the machine learning compound with PubChem ID: 11152946 compared to PubChem IDs: 71465645 and 68155180. A 100 ns molecular dynamics simulation confirmed the conformational stability of the compound-receptor complex, demonstrating that hydrophobic interactions and hydrogen bonding predominantly couple the machine learning-based compound with the protein receptor. Notably, compounds with PubChem IDs: 71465645, 11152946 and 68155180 exhibited significant inhibitory effects on VEGFR-1, VEGFR-2, and VEGFR-3, respectively. These findings underscore the potential of these compounds for further exploration and validation through in-vitro research, particularly for understanding their pharmacodynamic characteristics and clinical pharmacokinetics in cervical cancer treatment.

### Supplementary Information

Supplementary Data 1.

Supplementary Data 2.

Supplementary Data 3.

Supplementary Data 4.

Supplementary Data 5.

Supplementary Table 1.

Supplementary Table 2.

Supplementary Table 3.

## Data Availability

The chemical structures and inhibitors data that support the findings of this study are available in Figshare with the identifier 10.6084/m9.figshare.24078207.v1.

## Code availability

The code used for running this study, model fitting, and plotting is available on a GitHub repository at https://github.com/AnurajNayarisseri/Vegfr_Cervical_Cancer.
