# Supplementary material for: Potential inhibitors of VEGFR1, VEGFR2, and VEGFR3 developed through Deep Learning for the treatment of Cervical Cancer
Source: Sci Rep. 2024 Jun 10;14:13251. doi: 10.1038/s41598-024-63762-w (PMC11164920; doi:10.1038/s41598-024-63762-w)
Supplement: Supplementary file 1 — Supplementary Data 1. [file 41598_2024_63762_MOESM1_ESM.docx]

**Supplementary Data I**

**The top 10 chemically tailored VEGFR1, VEGFR2 and VEGFR3 compounds obtained from Deep Learning –**

I. The top 11 chemically tailored VEGFR1 compounds obtained from Deep Learning –

| 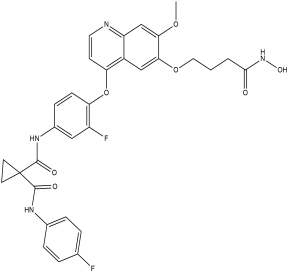 | 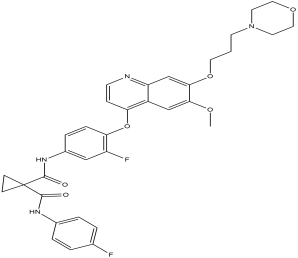 | 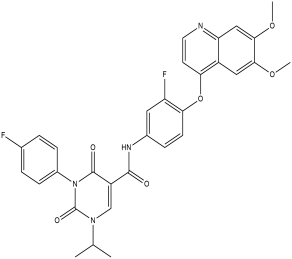 | 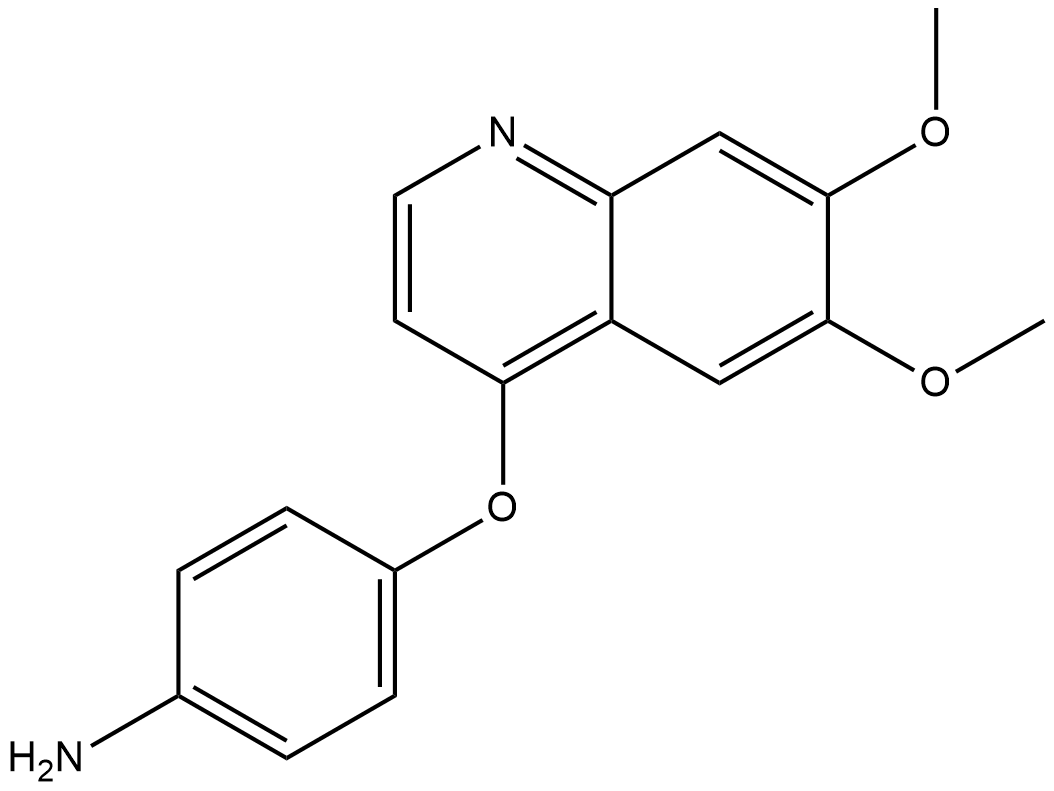 |
| --- | --- | --- | --- |
| **PubChem CID:71465645** | **PubChem CID: 42642645** | **PubChemCID:71576419** | **PubChem CID:11594543** |
| 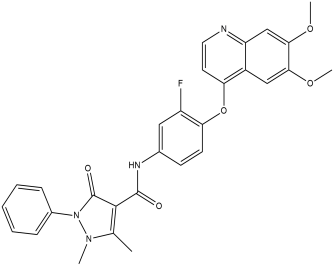 | **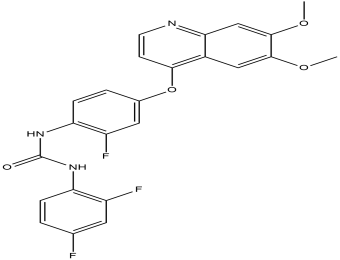** | **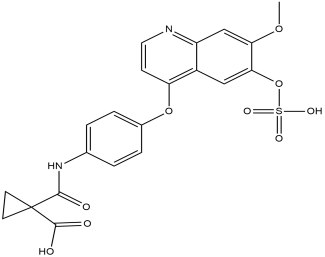** | **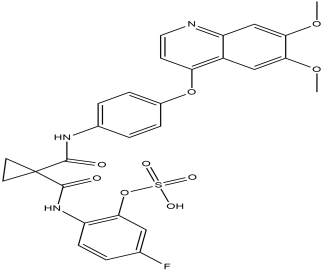** |
| **PubChem CID:56604907** | **PubChem CID: 11317348** | **PubChem CID:6269669** | **PubChem CID: 86269462** |
| 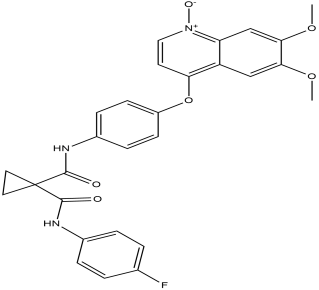 | **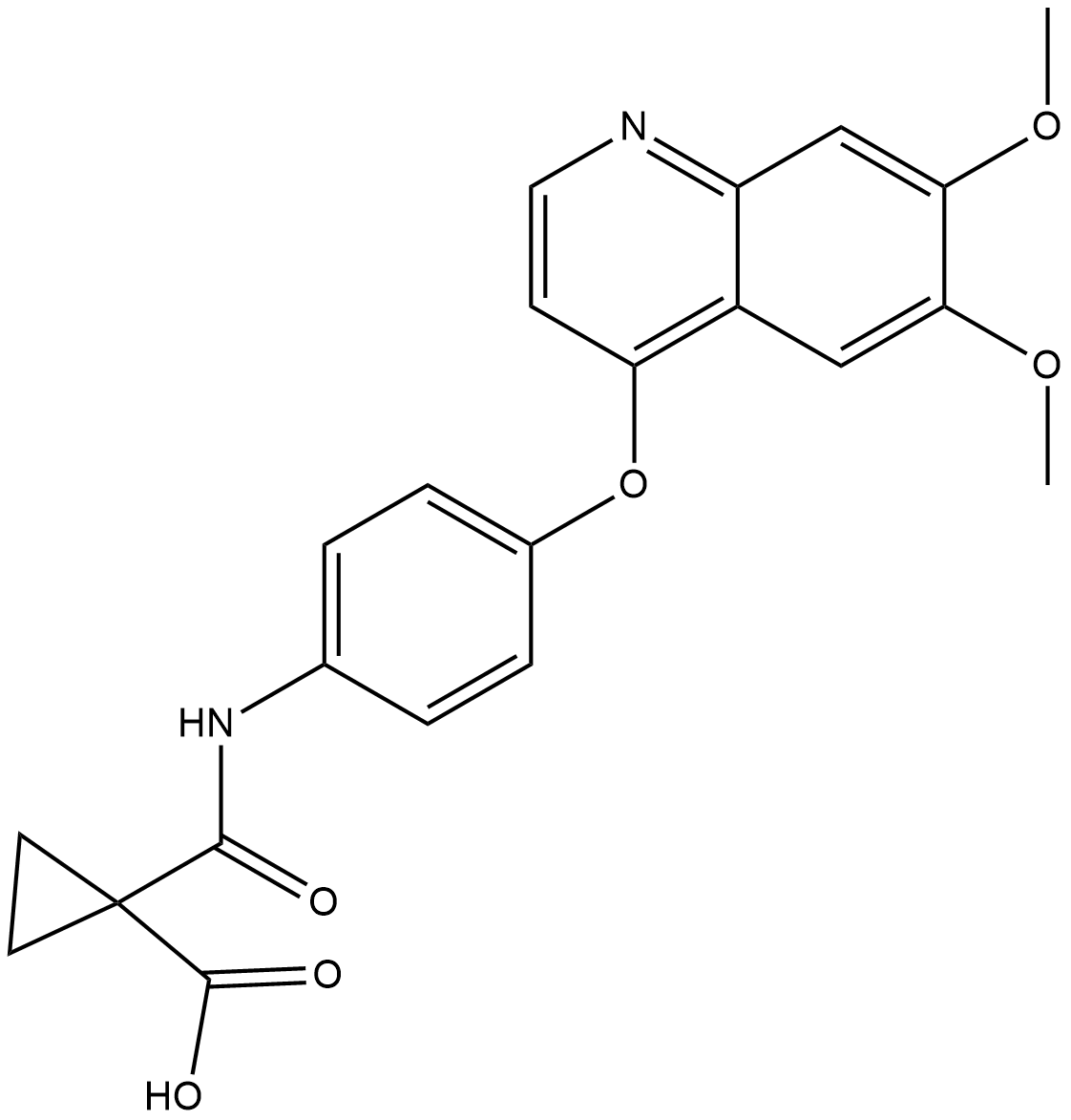** | **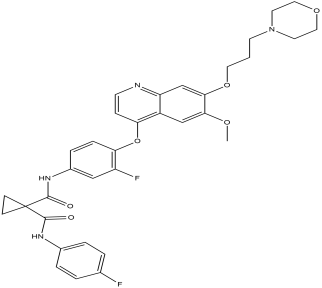** |  |
| **PubChem CID:78325042** | **PubChem CID: 57810164** | **PubChem CID: 6189868** |  |

II. The top 12 chemically tailored VEGFR2 compounds obtained from Deep Learning –

| 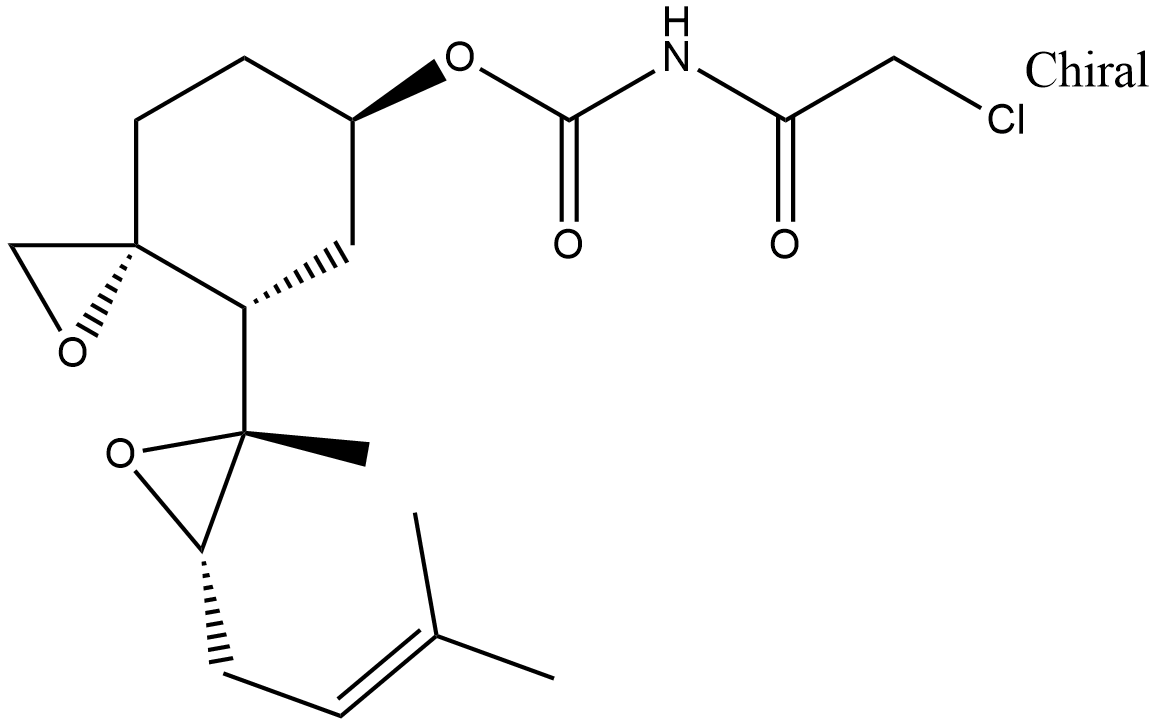 | 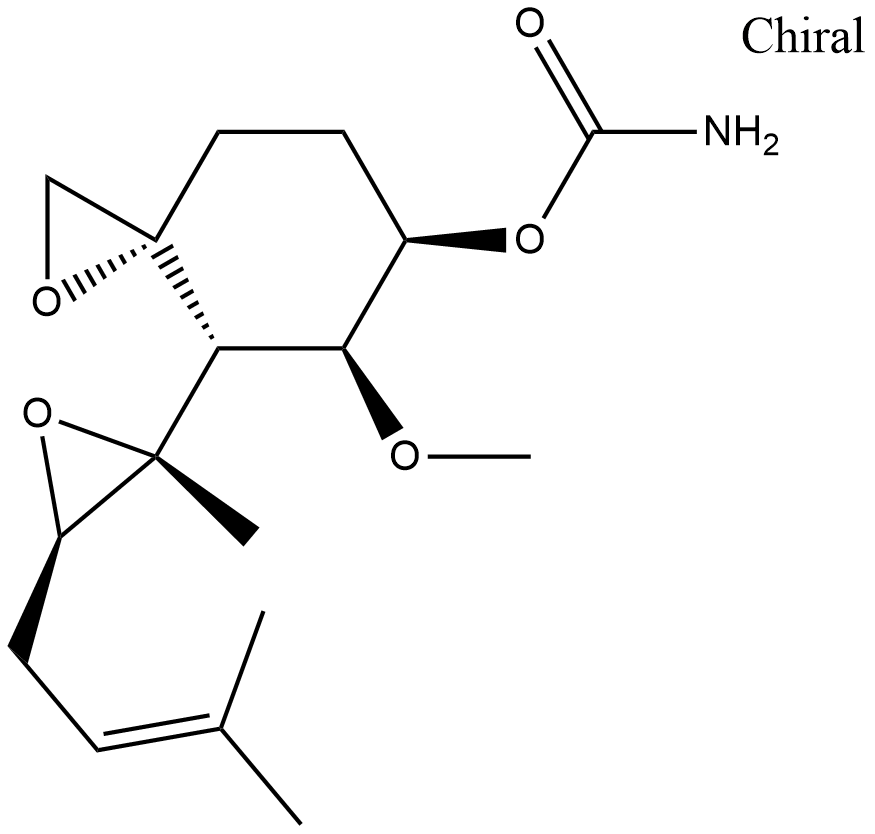 | 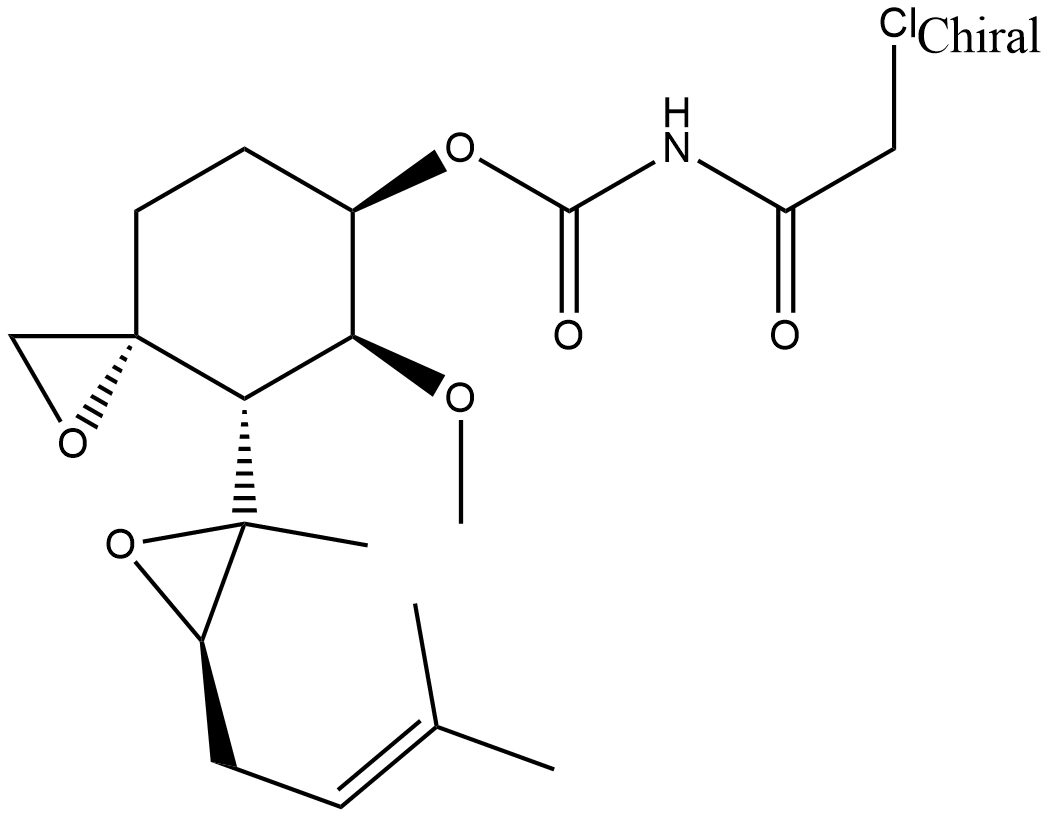 | 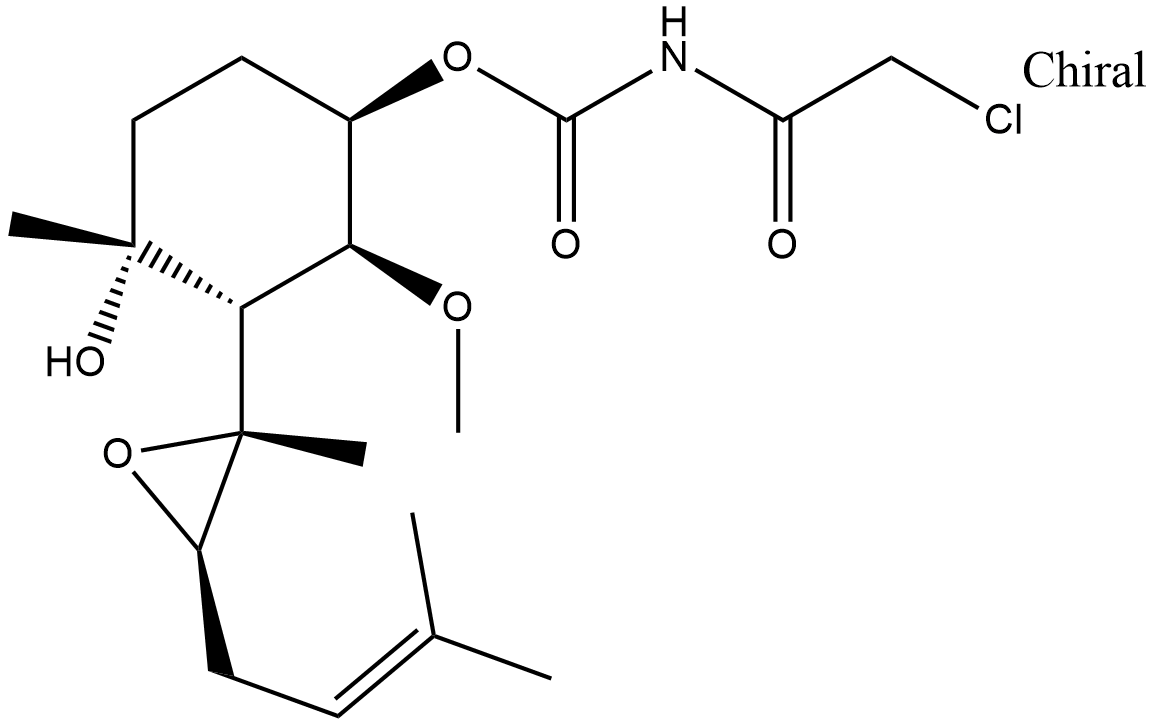 |
| --- | --- | --- | --- |
| **PubChem CID:11152946** | **PubChem CID: 125367** | **PubChem CID: 9930932** | **PubChem CID: 5326425** |
| 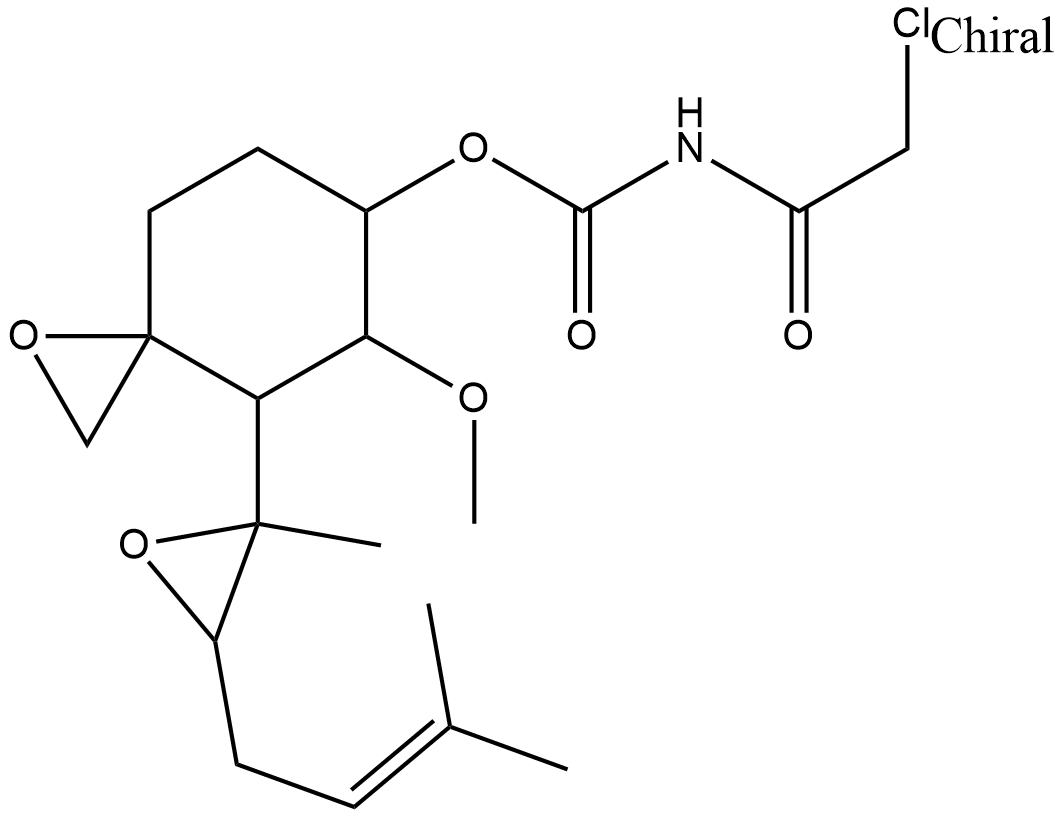 | **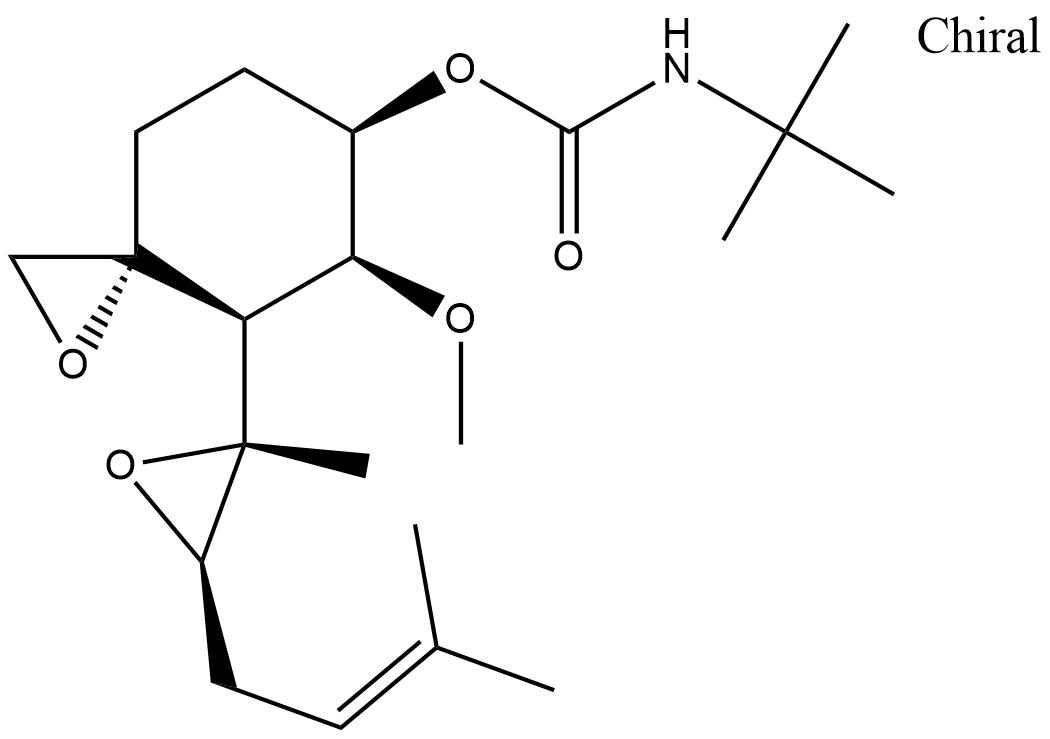** | **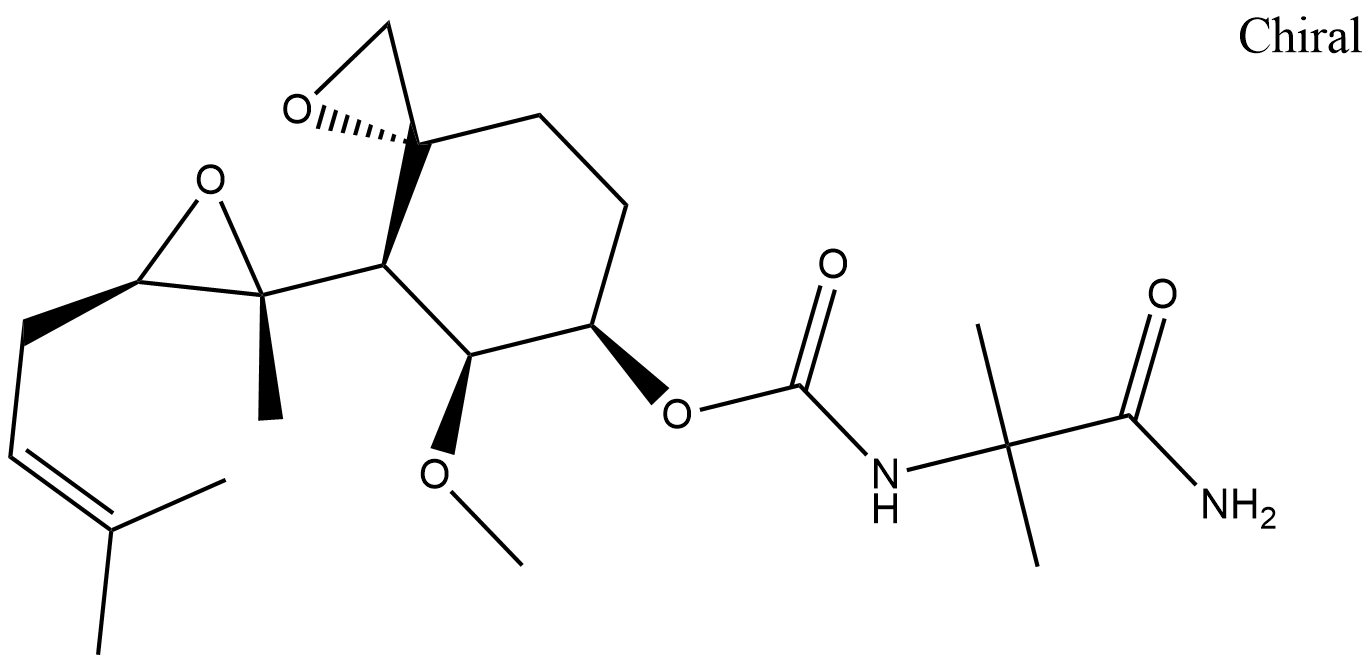** | **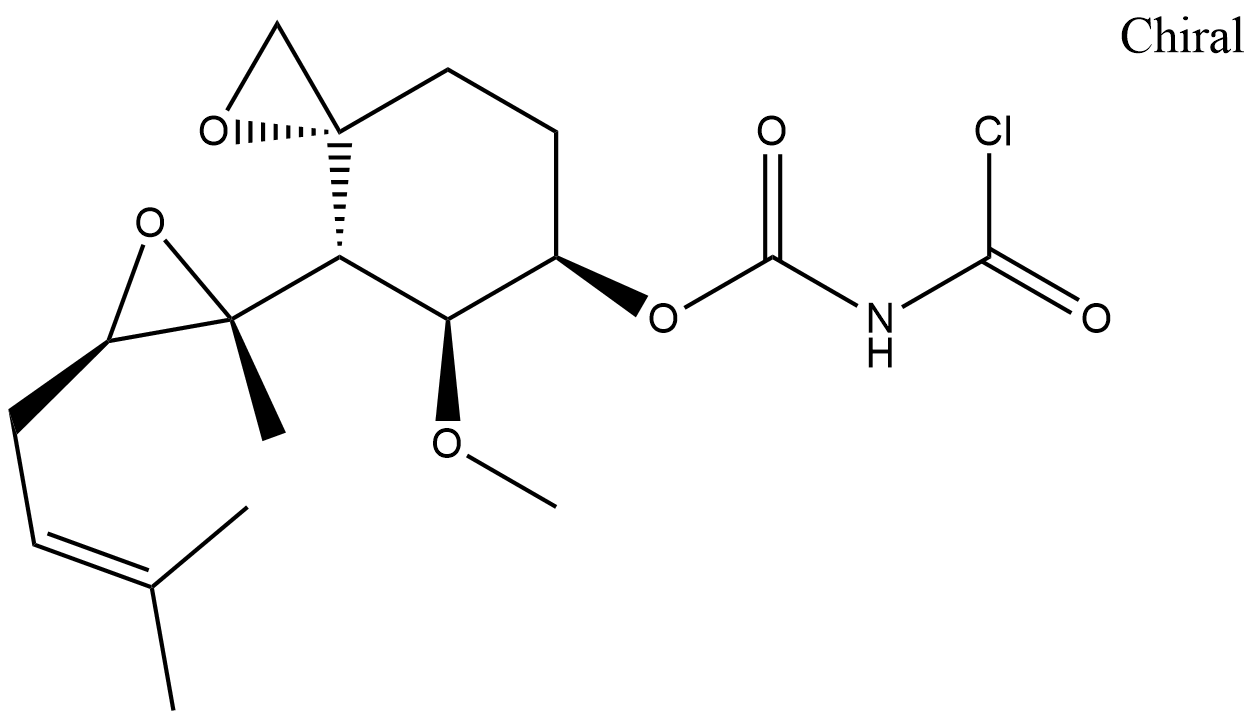** |
| **PubChem CID: 60791** | **PubChem CID: 45482966** | **PubChem CID: 45482954** | **PubChem CID: 44385040** |
| 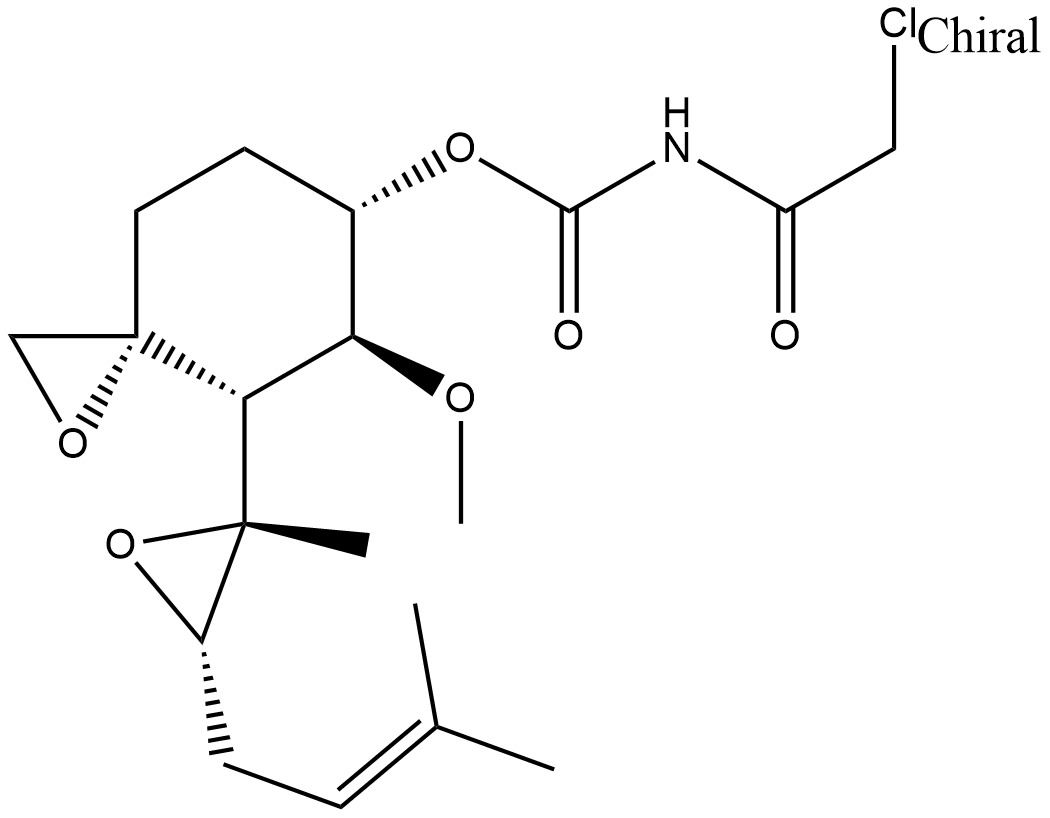 | **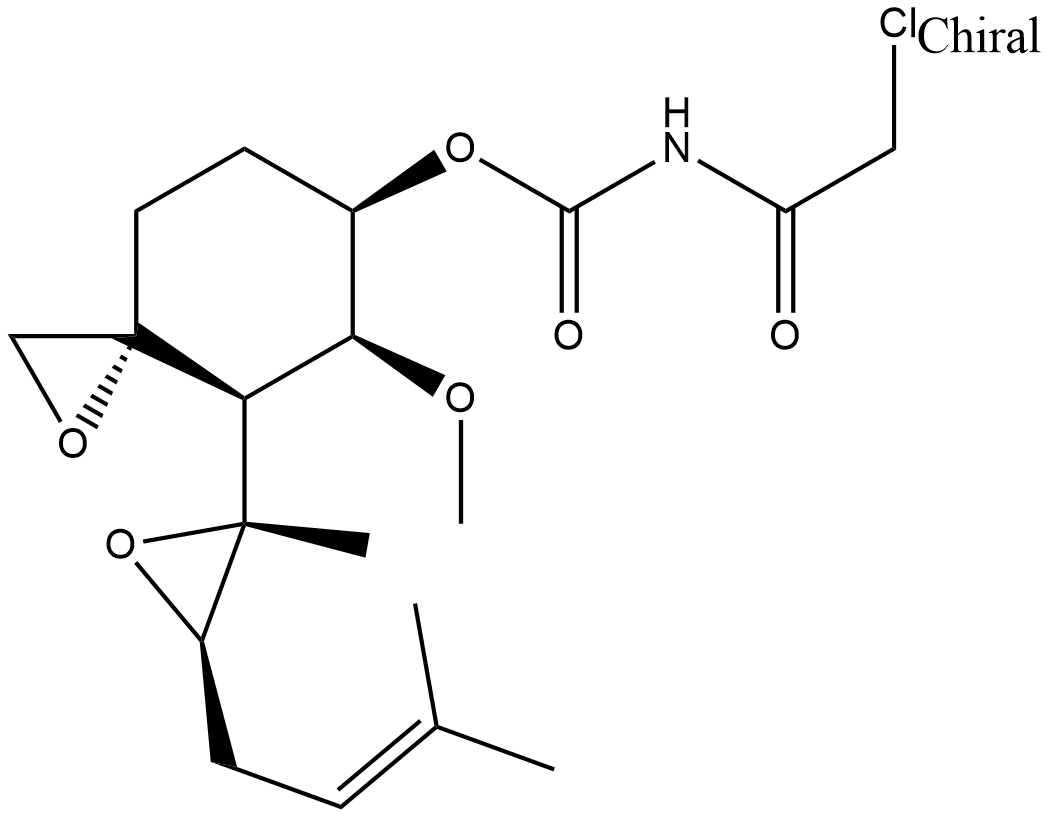** | **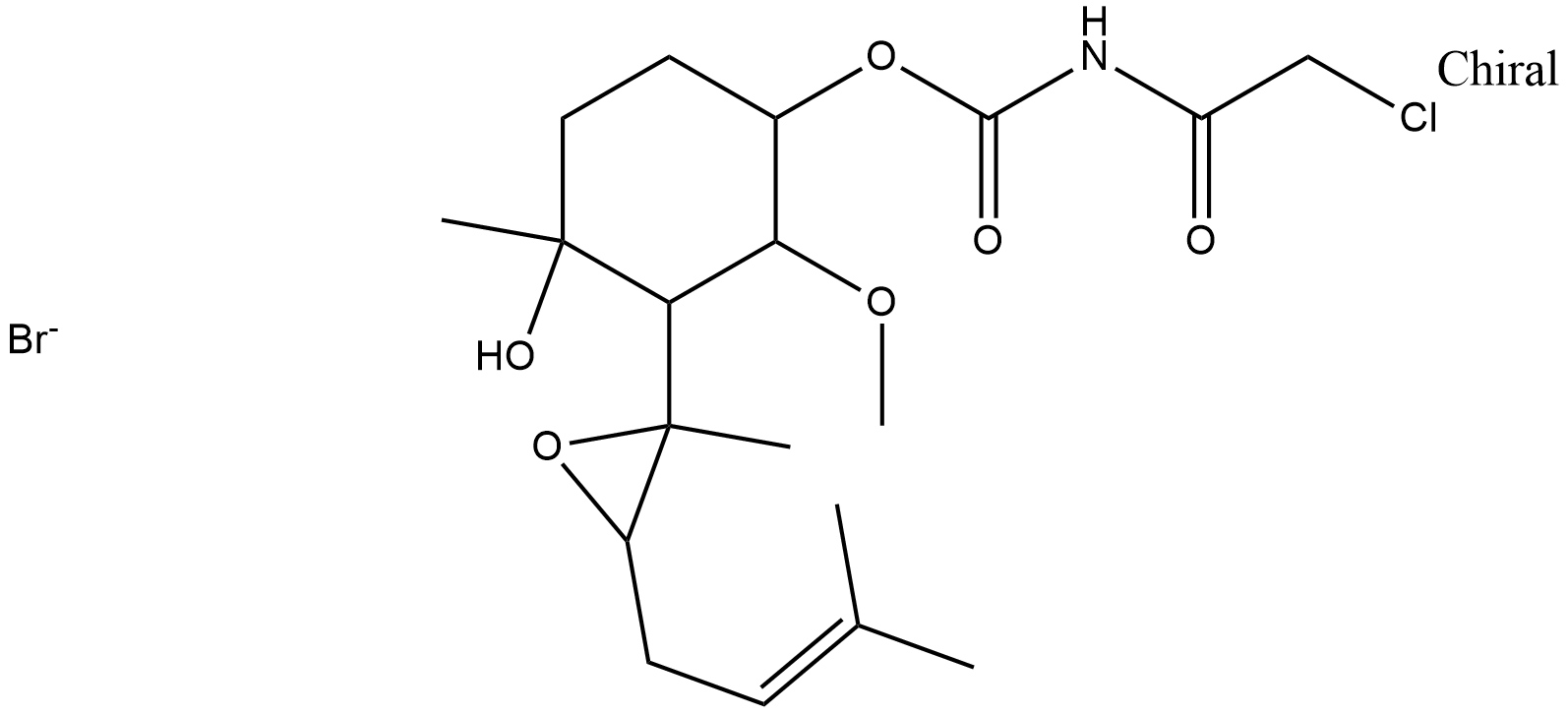** | **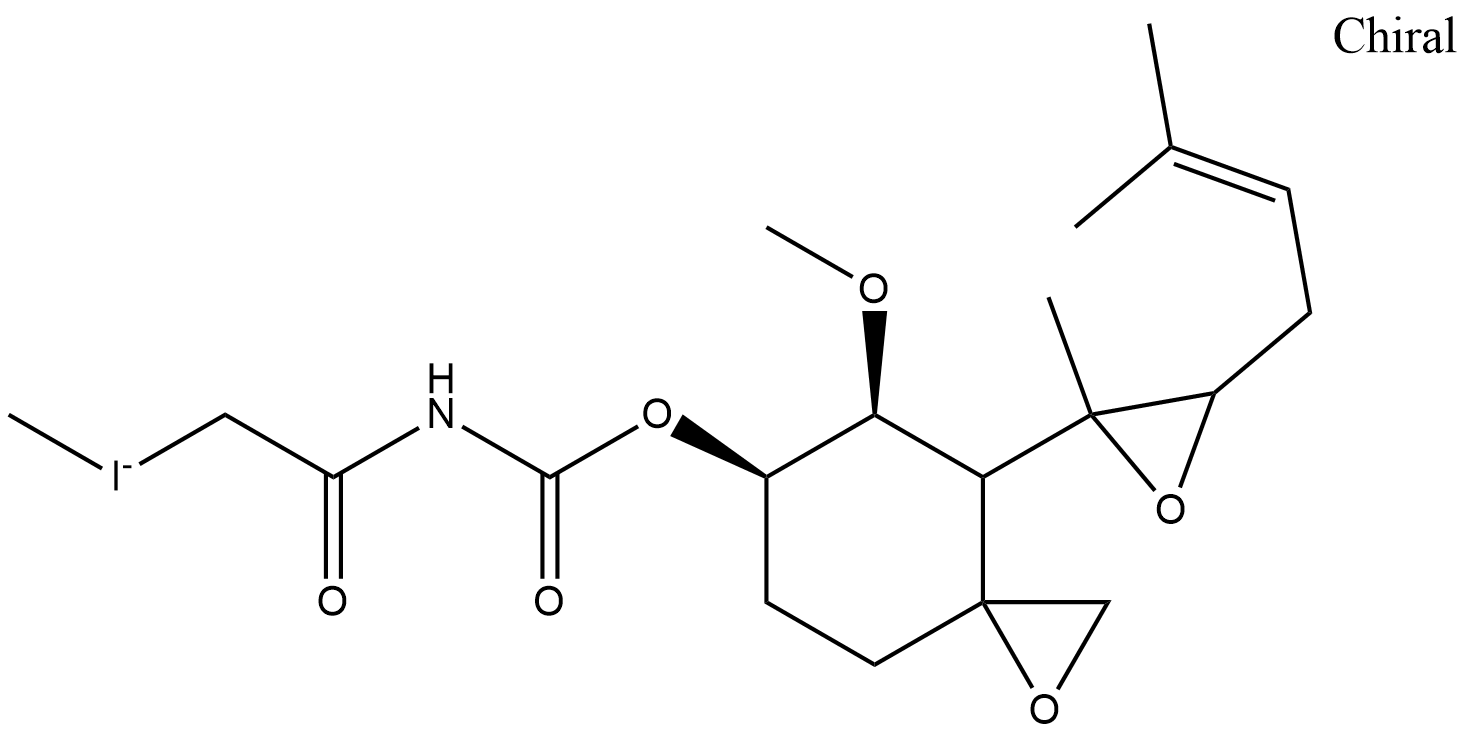** |
| **PubChem CID:44343813** | **PubChem CID: 14942882** | **PubChem CID: 163806427** | **PubChem CID: 163508288** |

III. The top 10 chemically tailored VEGFR3 compounds obtained from Deep Learning –

| 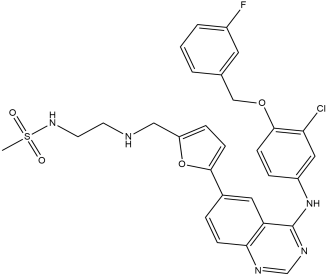 | 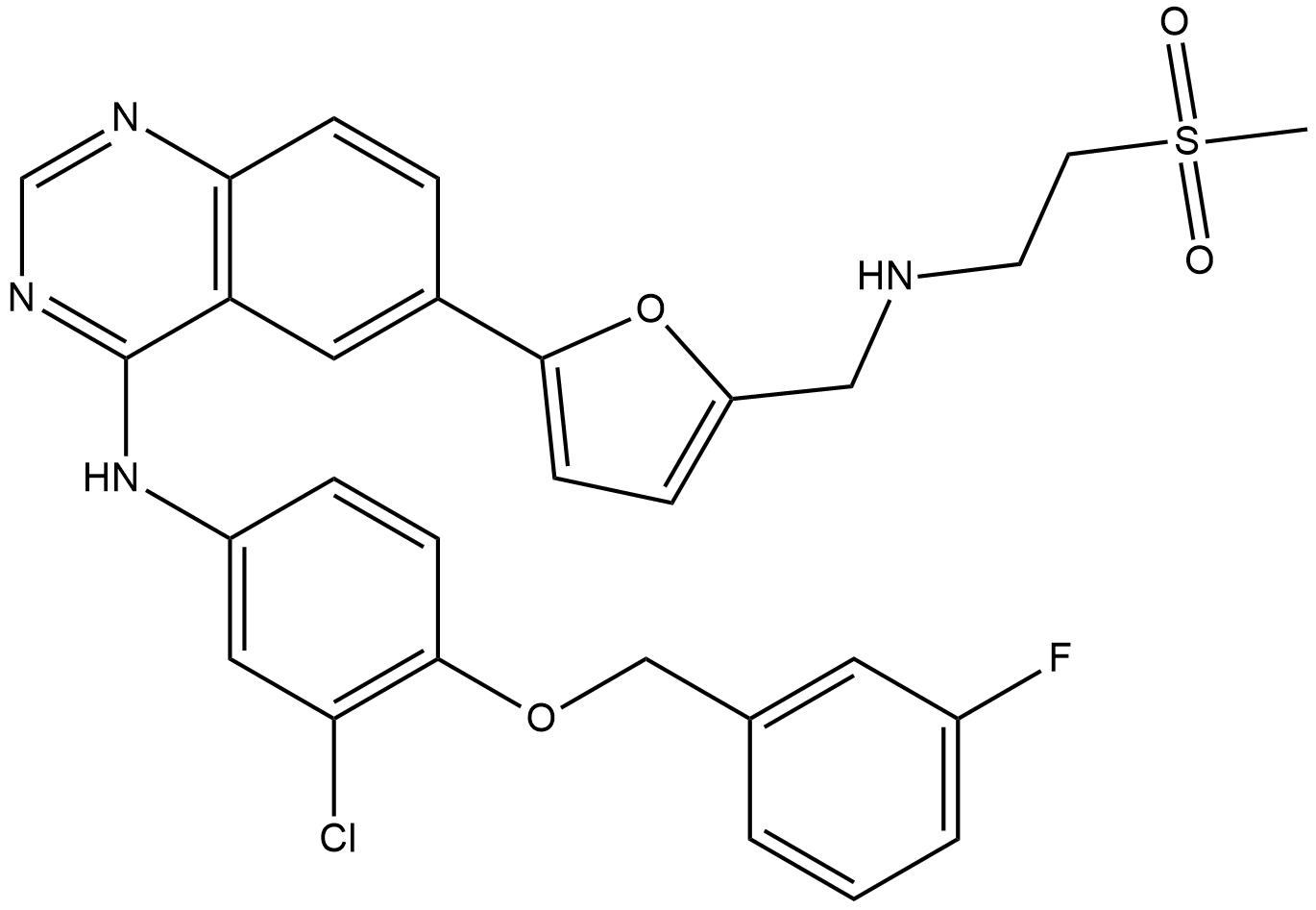 | 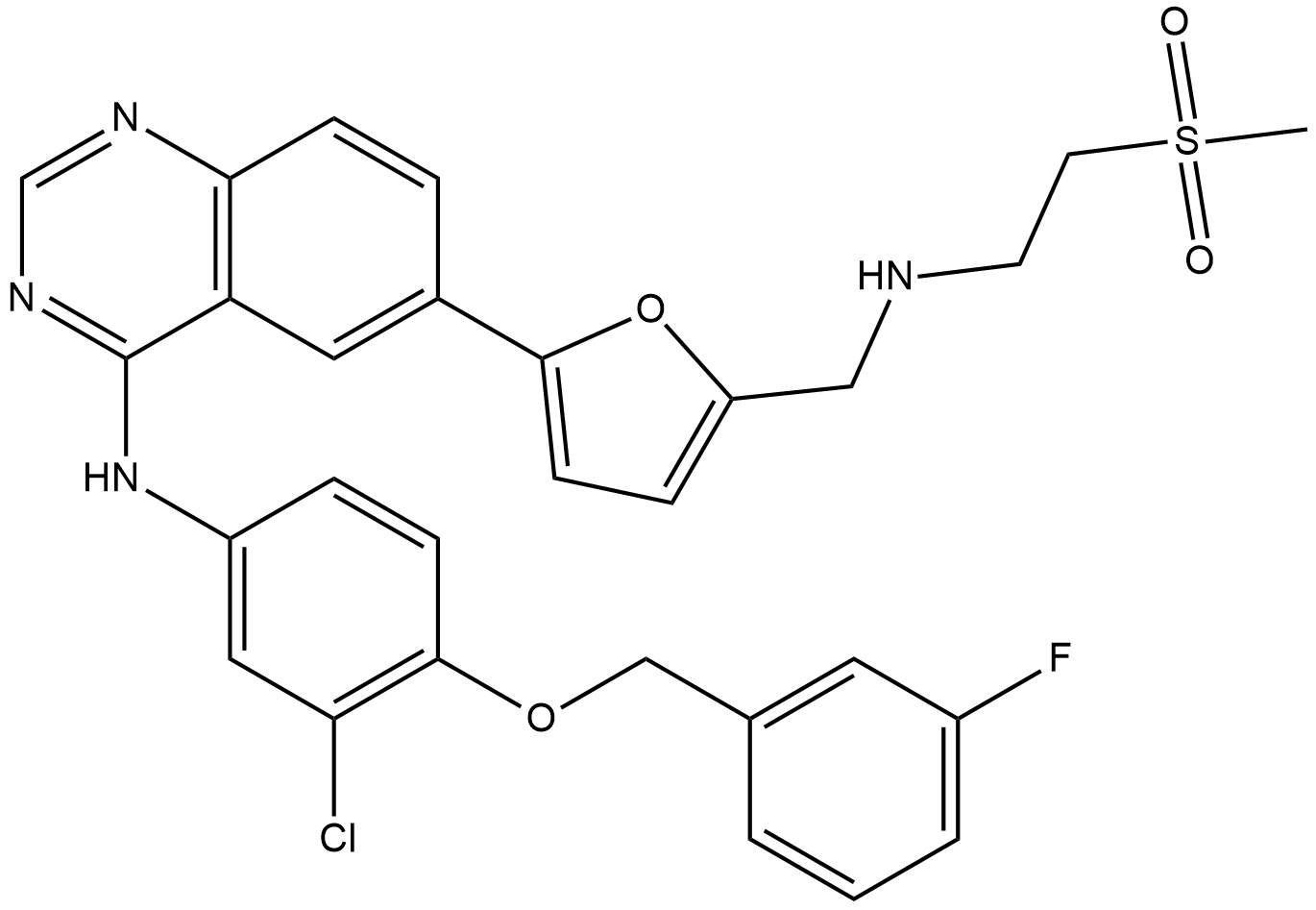 | 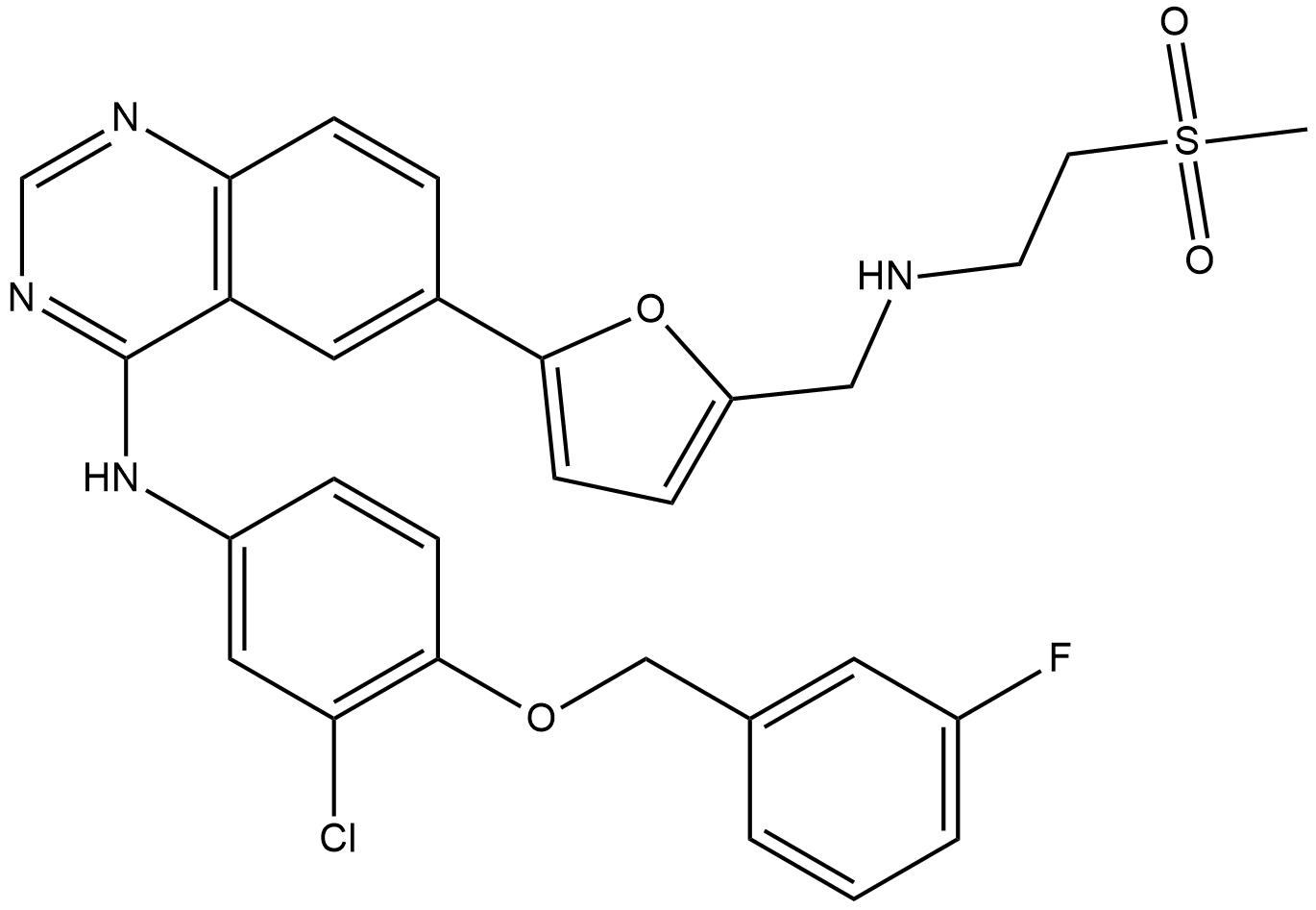 |
| --- | --- | --- | --- |
| **PubChem CID: 68155180** | **PubChem CID: 9941095** | **PubChem CID: 91798457** | **PubChem CID: 11679357** |
| **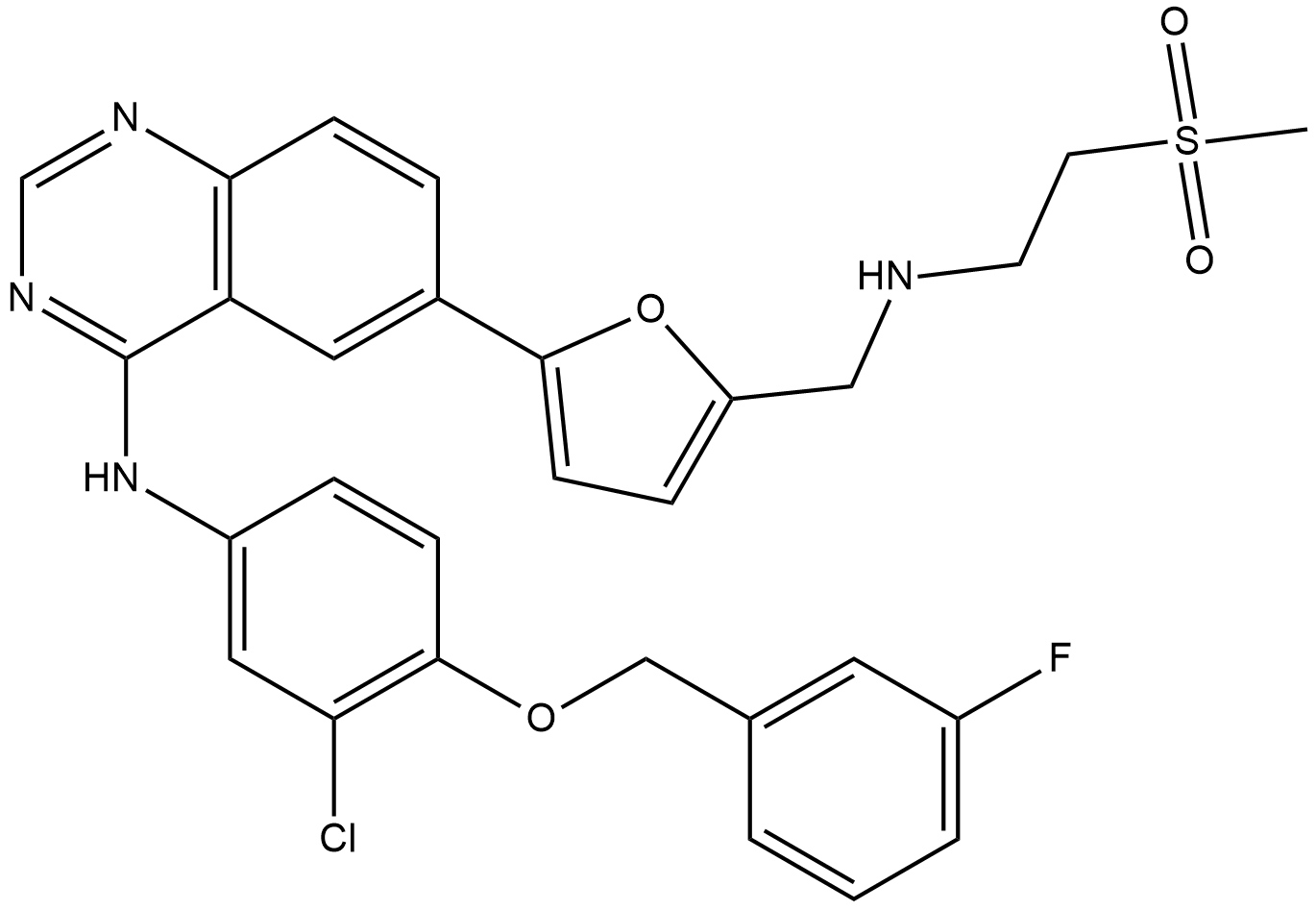** | **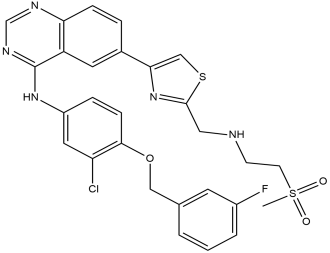** | **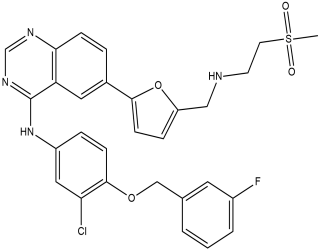** | **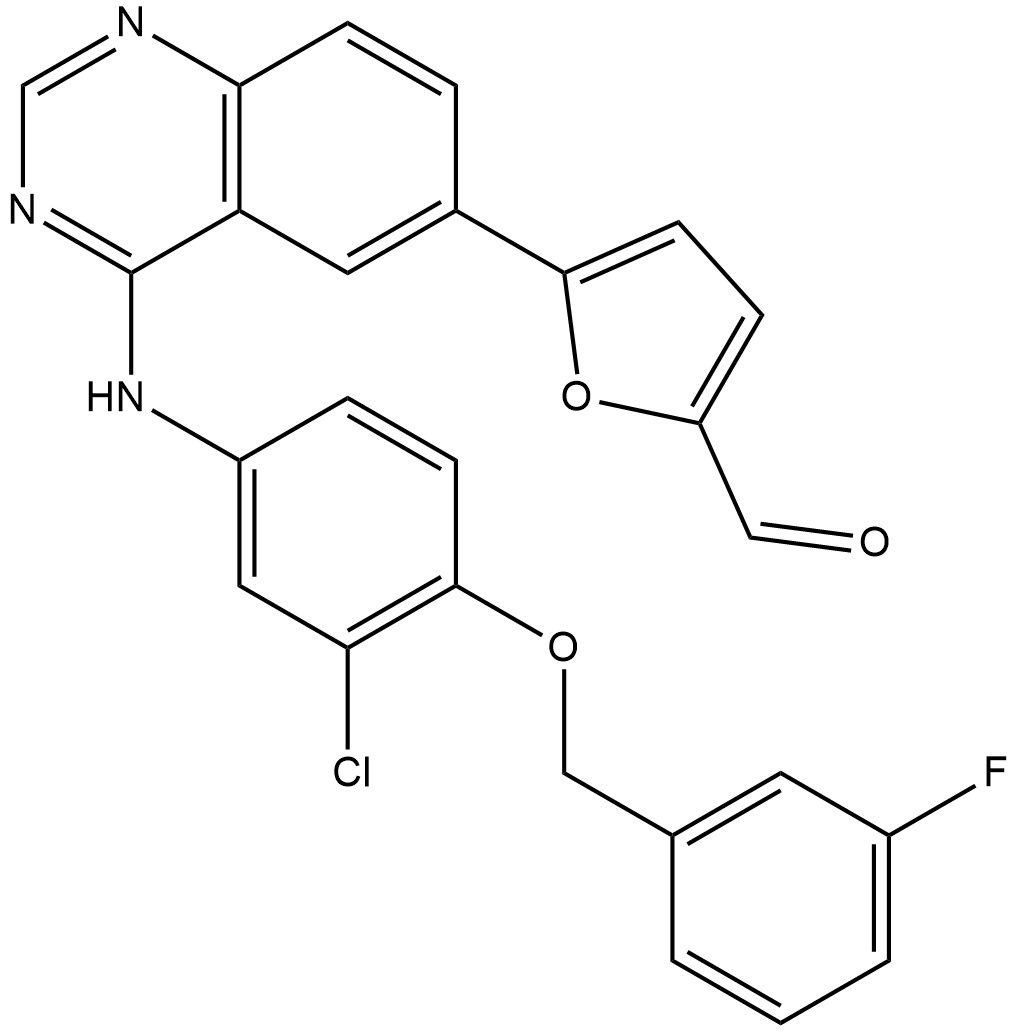** |
| **PubChem CID: 208909** | **PubChem CID: 16219404** | **PubChem CID: 139061731** | **PubChem CID: 11181296** |
| **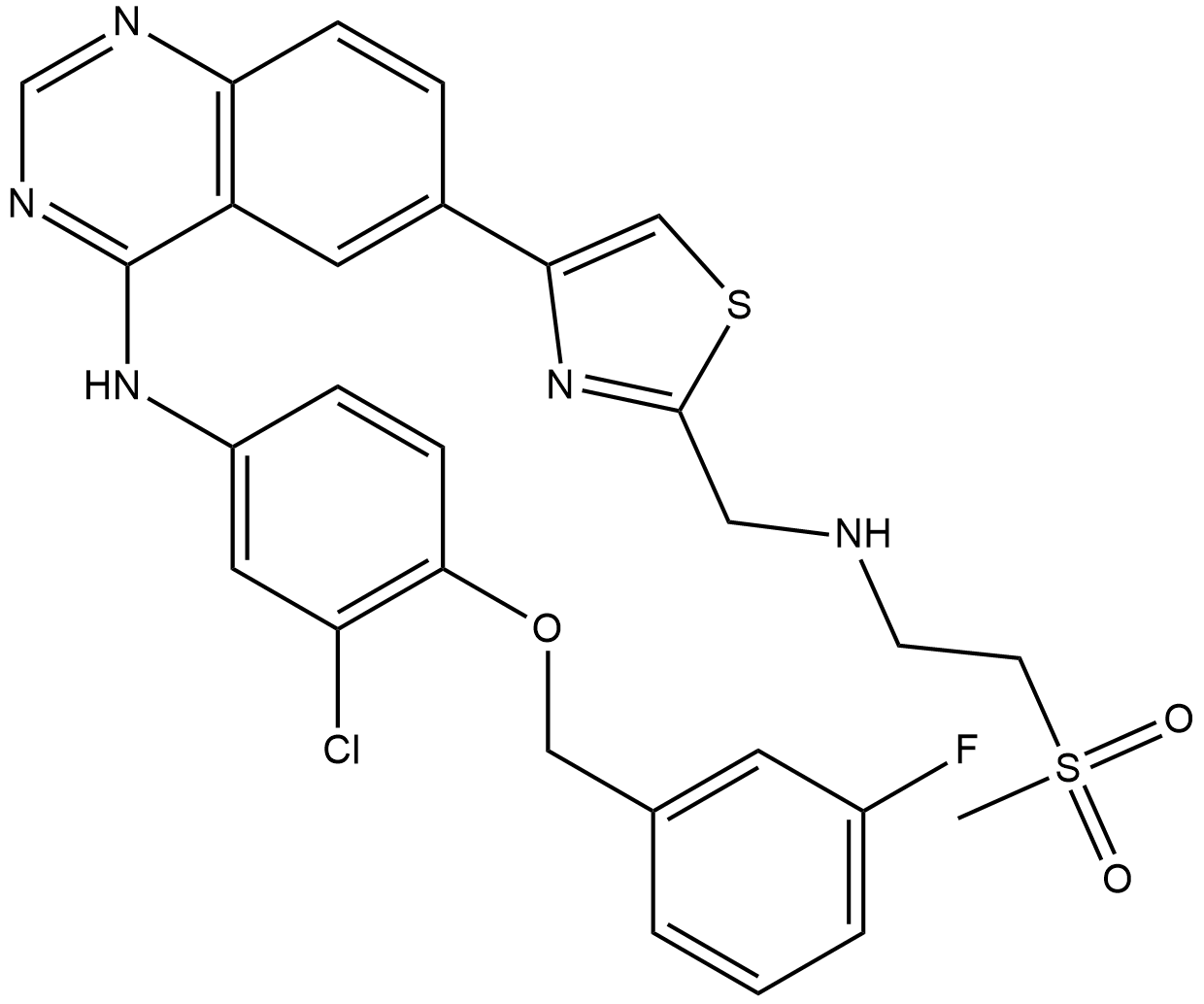** | **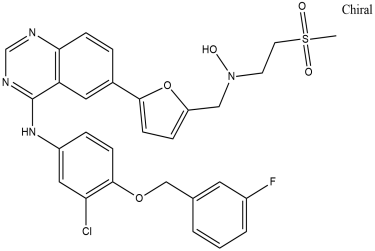** |  |  |
| **PubChem CID: 5329480** | **PubChem CID: 118753054** |  |  |
