# Supplementary material for: Potential inhibitors of VEGFR1, VEGFR2, and VEGFR3 developed through Deep Learning for the treatment of Cervical Cancer
Source: Sci Rep. 2024 Jun 10;14:13251. doi: 10.1038/s41598-024-63762-w (PMC11164920; doi:10.1038/s41598-024-63762-w)
Supplement: Supplementary file 2 — Supplementary Data 2. [file 41598_2024_63762_MOESM2_ESM.docx]

**Supplementary Data II**

**Interaction diagrams of VEGFR-1, VEGFR-II, VEGFR-III complex with best established compound and ML generated compound observed during the molecular dynamic’s simulation –**


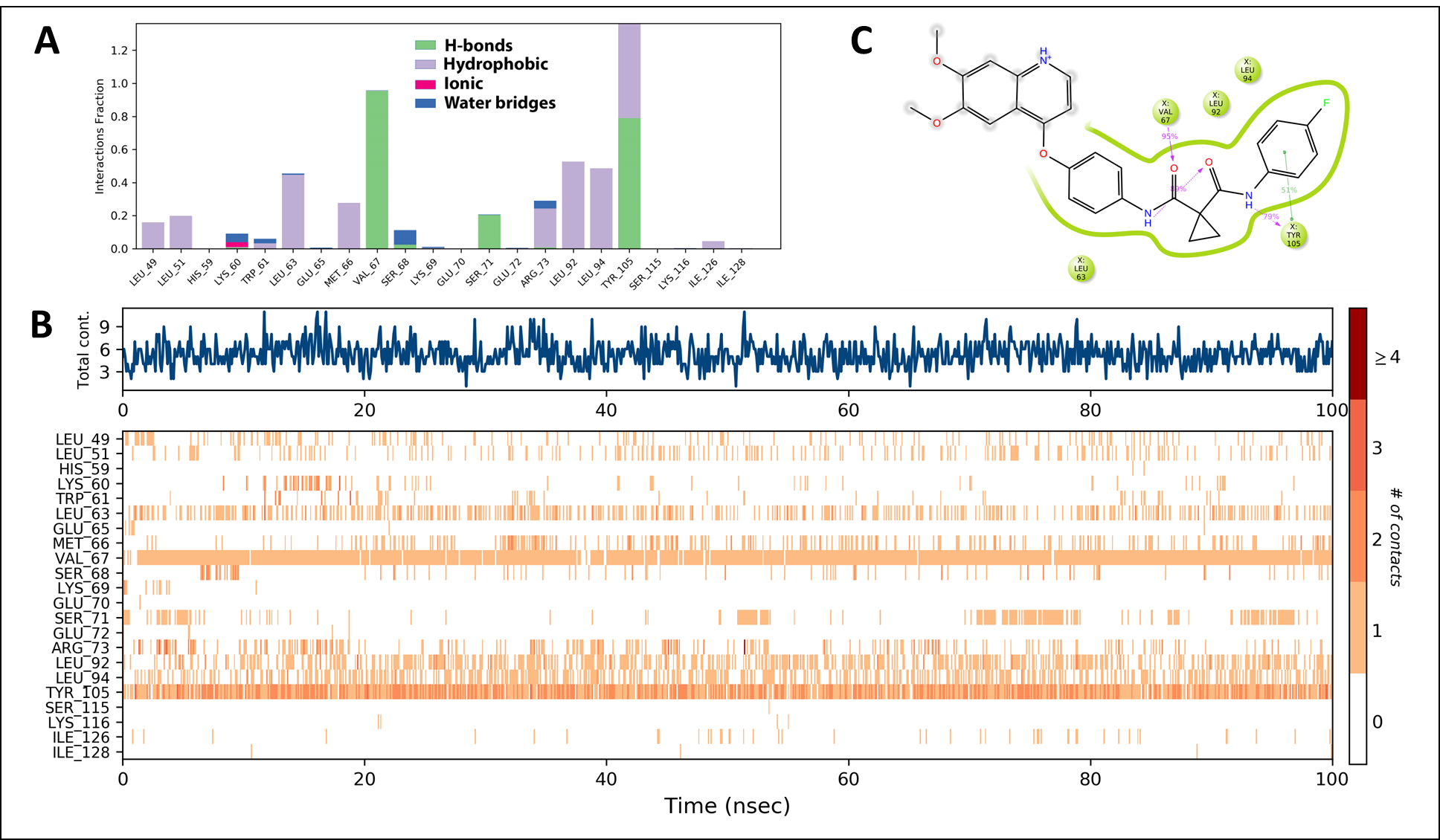


**Figure I**. Interaction diagram of VEGFR-1 complex with best established compound PubChem ID: 25102847 observed during the molecular dynamic’s simulation. (A) The protein-ligand interaction diagram. (B) The residues that interact with the ligand in each trajectory frame. (C) Schematic diagram of ligand interaction with the amino acid residues of protein during MD simulation.


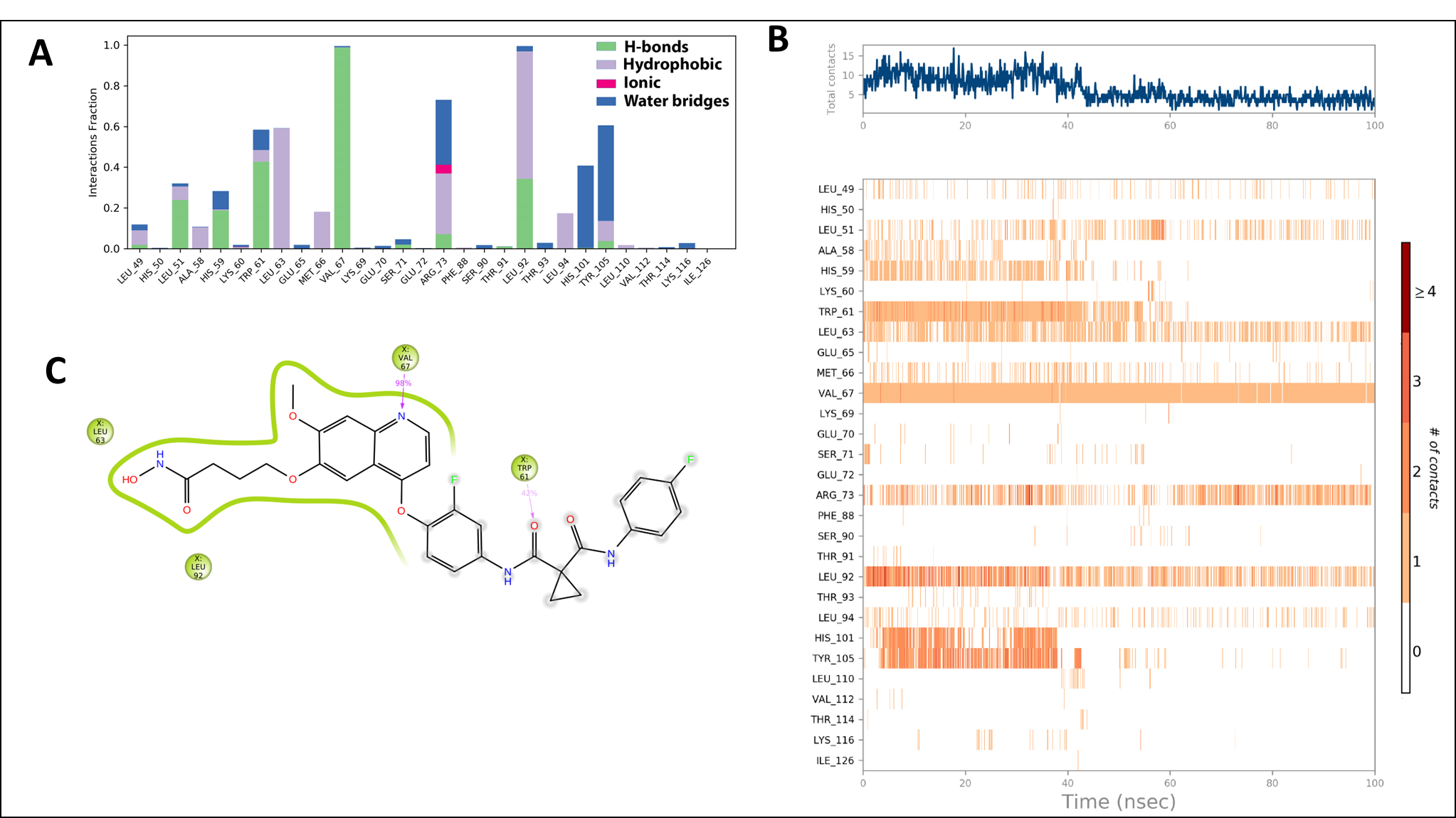


**Figure II.** Interaction diagram of VEGFR-1 complex with best ML Model compound PubChem ID: 71465645 observed during the molecular dynamic’s simulation. (A) The protein-ligand interaction diagram. (B) The residues that interact with the ligand in each trajectory frame. (C) Schematic diagram of ligand interaction with the amino acid residues of protein during MD Simulation.


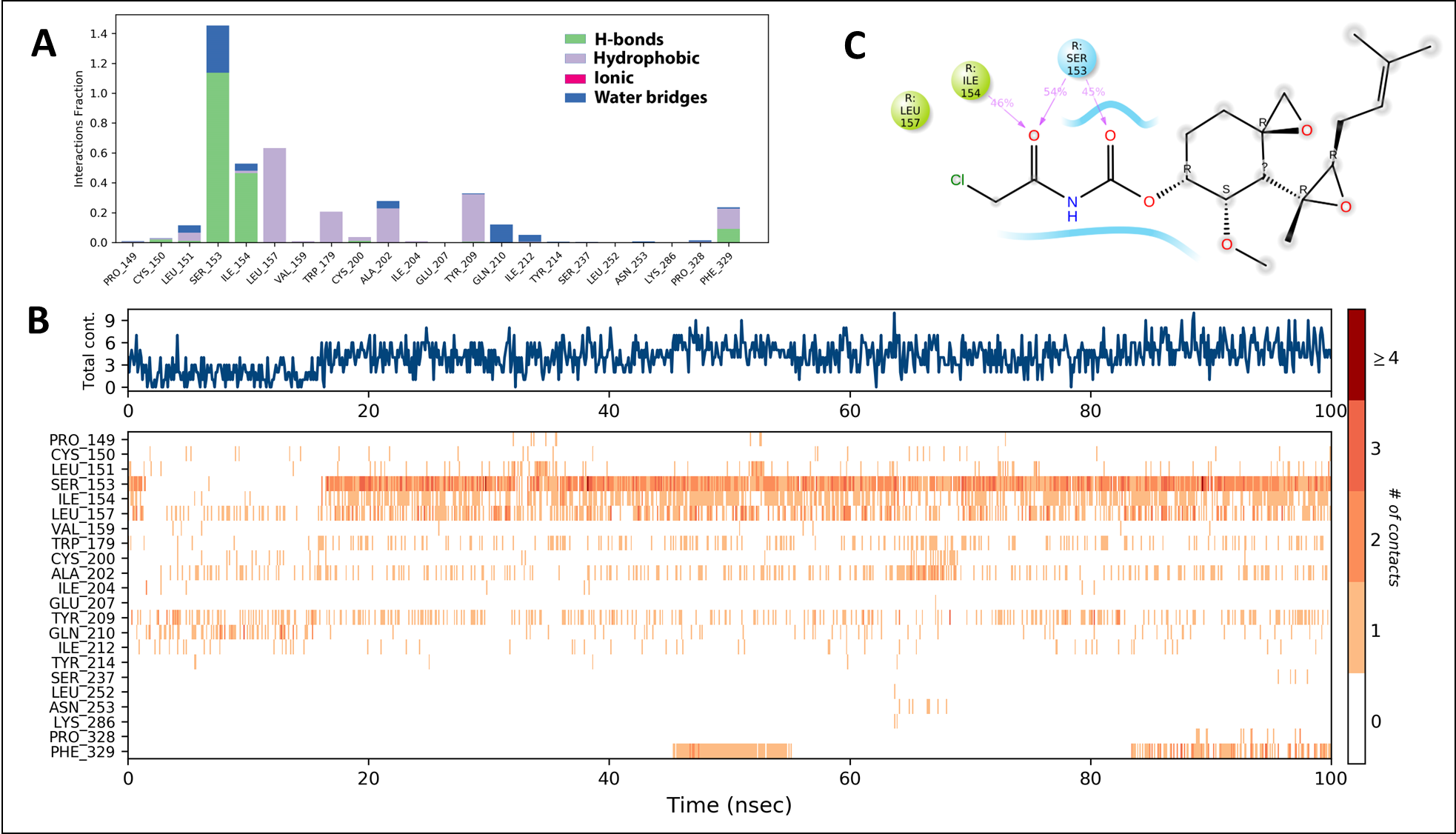


**Figure III.** Interaction diagram of VEGFR-2 complex with best established compound PubChem ID: 369976 observed during the molecular dynamic simulation. (A) The protein-ligand interaction diagram. (B) The residues that interact with the ligand in each trajectory frame. (C) Schematic diagram of ligand interaction with the amino acid residues of protein during MD simulation.


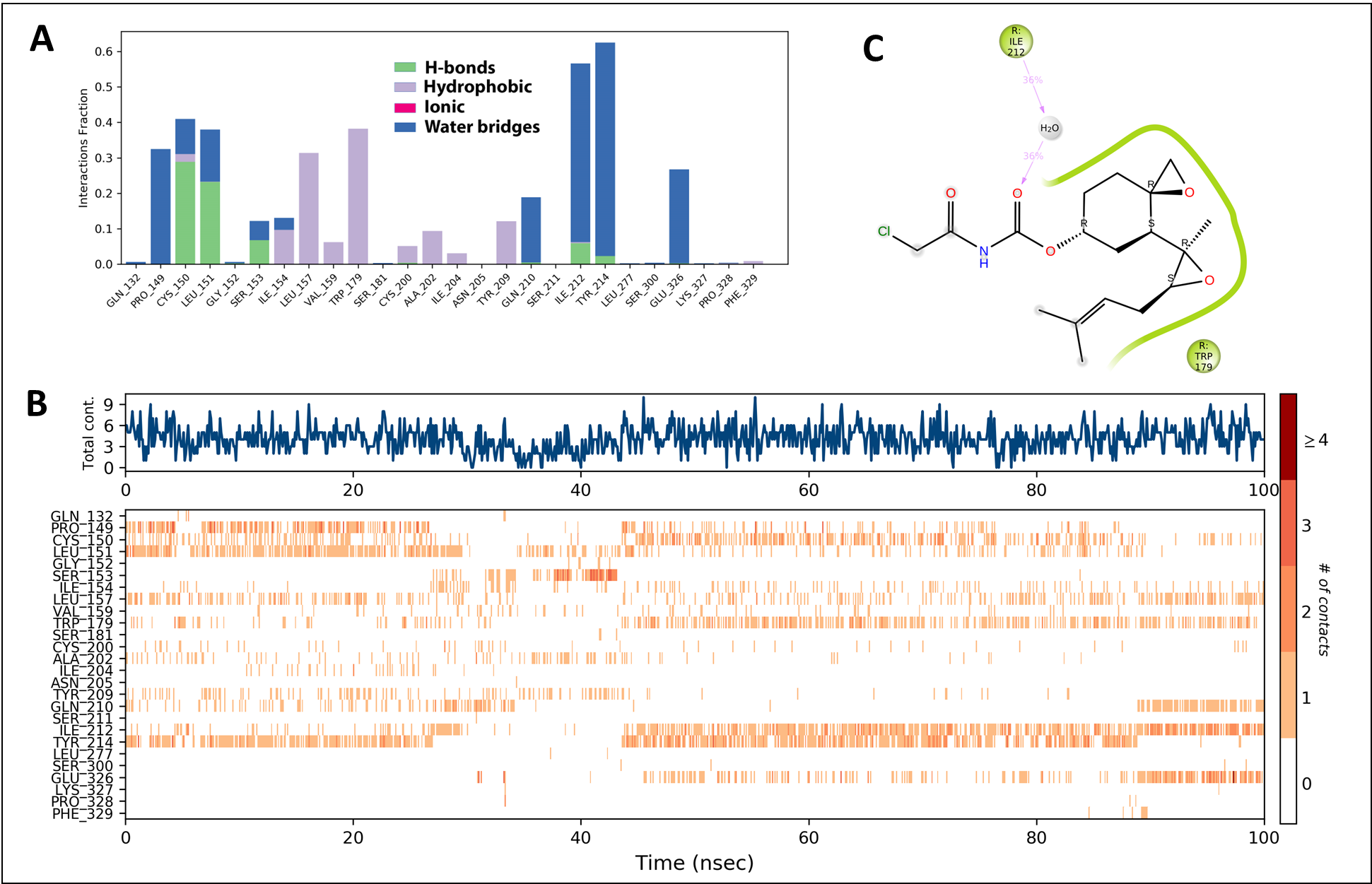


**Figure IV**. Interaction diagram of VEGFR-2 complex with best ML Model compound PubChem ID: 11152946 observed during the molecular dynamic’s simulation. (A) The protein-ligand interaction diagram. (B) The residues that interact with the ligand in each trajectory frame. (C) Schematic diagram of ligand interaction with the amino acid residues of protein during MD simulation.


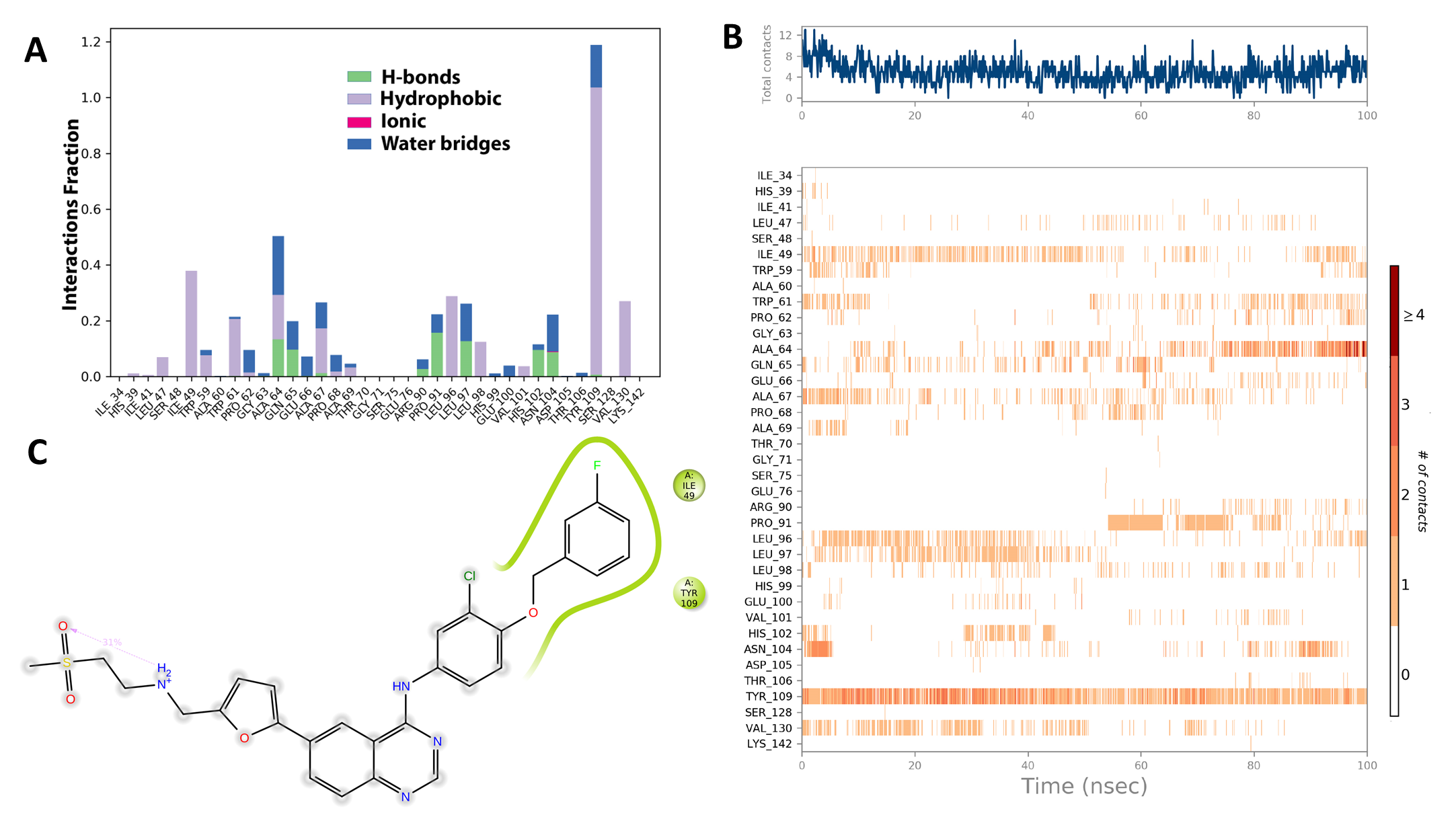


**Figure V**. Interaction diagram of VEGFR-3 complex with best established compound PubChem ID: 208908 observed during the molecular dynamic’s simulation. (A) The protein-ligand interaction diagram. (B) The residues that interact with the ligand in each trajectory frame. (C) Schematic diagram of ligand interaction with the amino acid residues of protein during MD simulation.


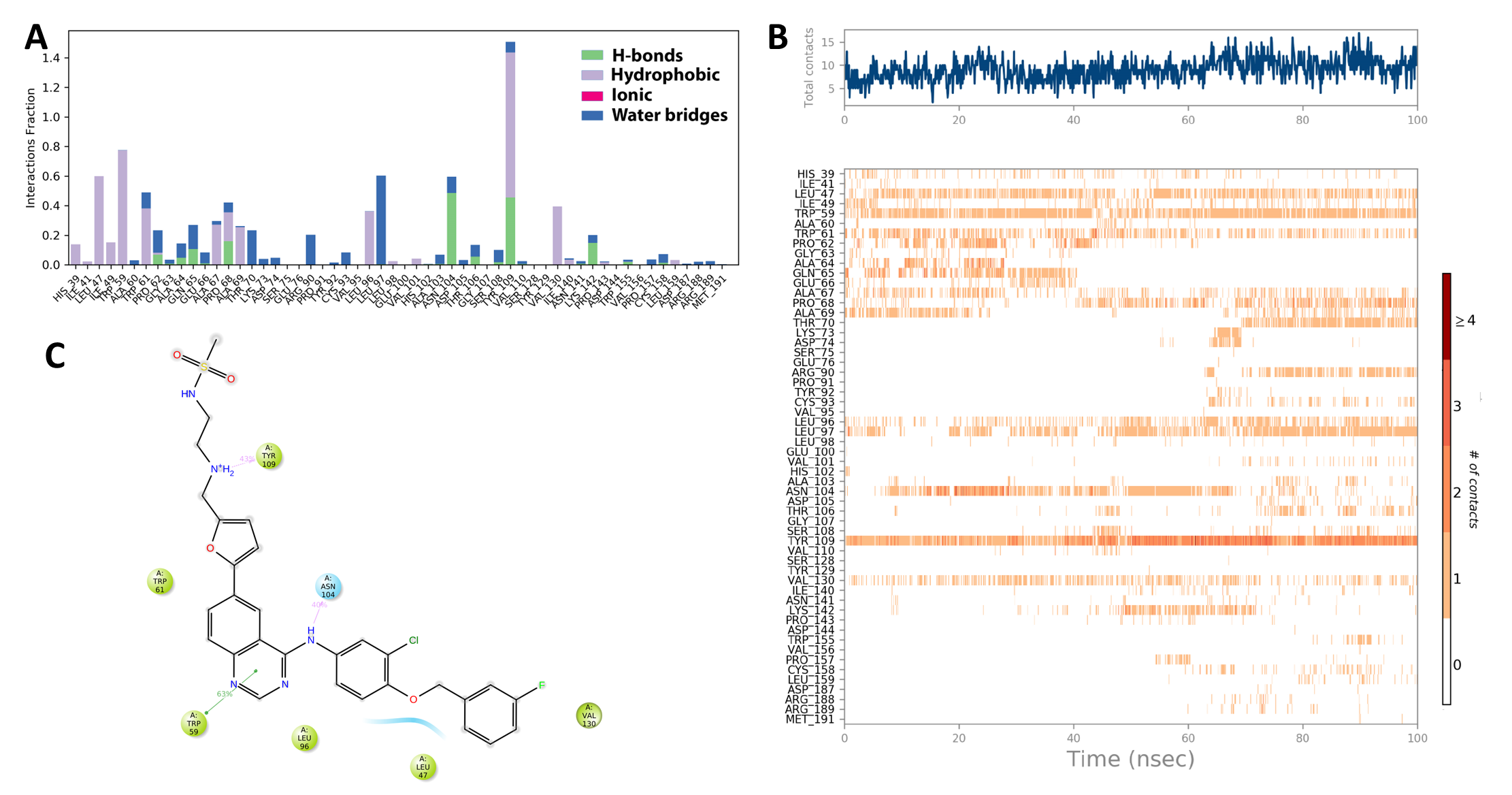


**Figure VI**. Interaction diagram of VEGFR-3 complex with best ML Model compound PubChem ID: 68155180 observed during the molecular dynamic simulation. (A) The protein-ligand interaction diagram. (B) The residues that interact with the ligand in each trajectory frame. (C) Schematic diagram of ligand interaction with the amino acid residues of protein during MD simulation.
