# Supplementary material for: Potential inhibitors of VEGFR1, VEGFR2, and VEGFR3 developed through Deep Learning for the treatment of Cervical Cancer
Source: Sci Rep. 2024 Jun 10;14:13251. doi: 10.1038/s41598-024-63762-w (PMC11164920; doi:10.1038/s41598-024-63762-w)
Supplement: Supplementary file 3 — Supplementary Data 3. [file 41598_2024_63762_MOESM3_ESM.docx]

**Supplementary Data III**

**Ligand property trajectory of the VEGFR-1, VEGFR-II and VEGFR-III complex with best established compound and Best ML generated compound during simulation period –**


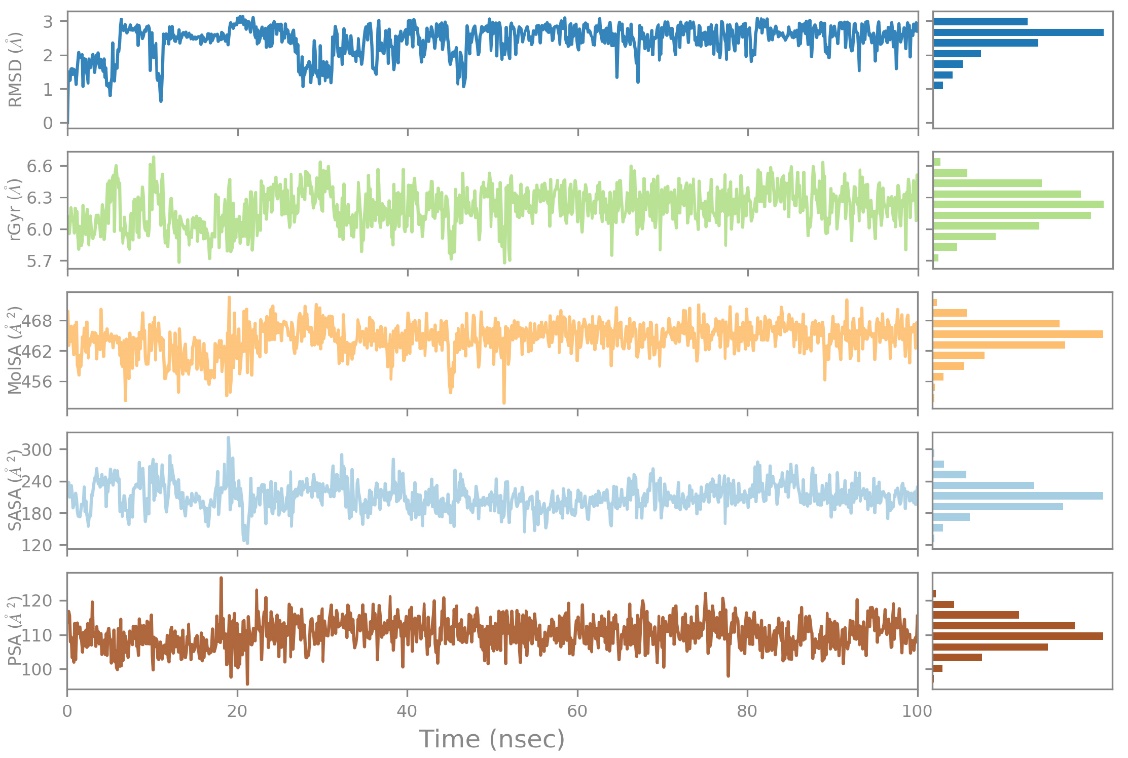


**Figure A.** The ligand property trajectory of the (VEGFR-1 complex with best established compound PubChem ID: 25102847) during the 100 ns simulation.


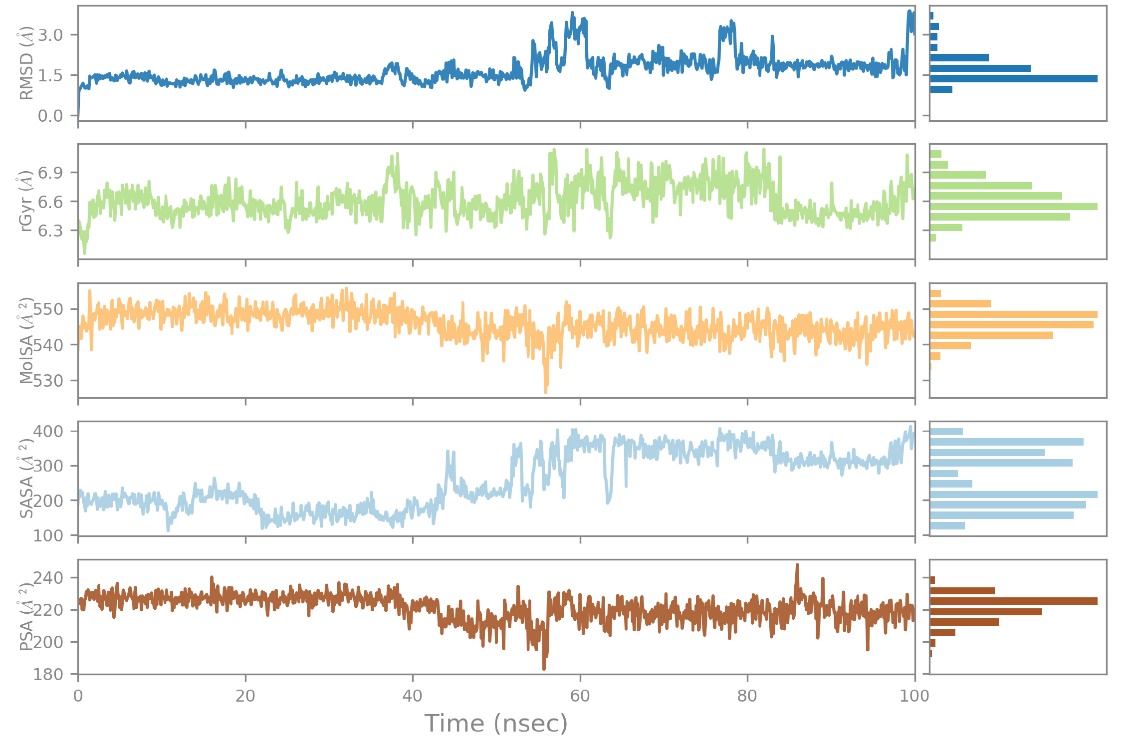


**Figure B.** The ligand property trajectory of the (VEGFR-1 complex with best ML Model compound PubChem ID:71465645) during the 100 ns simulation.


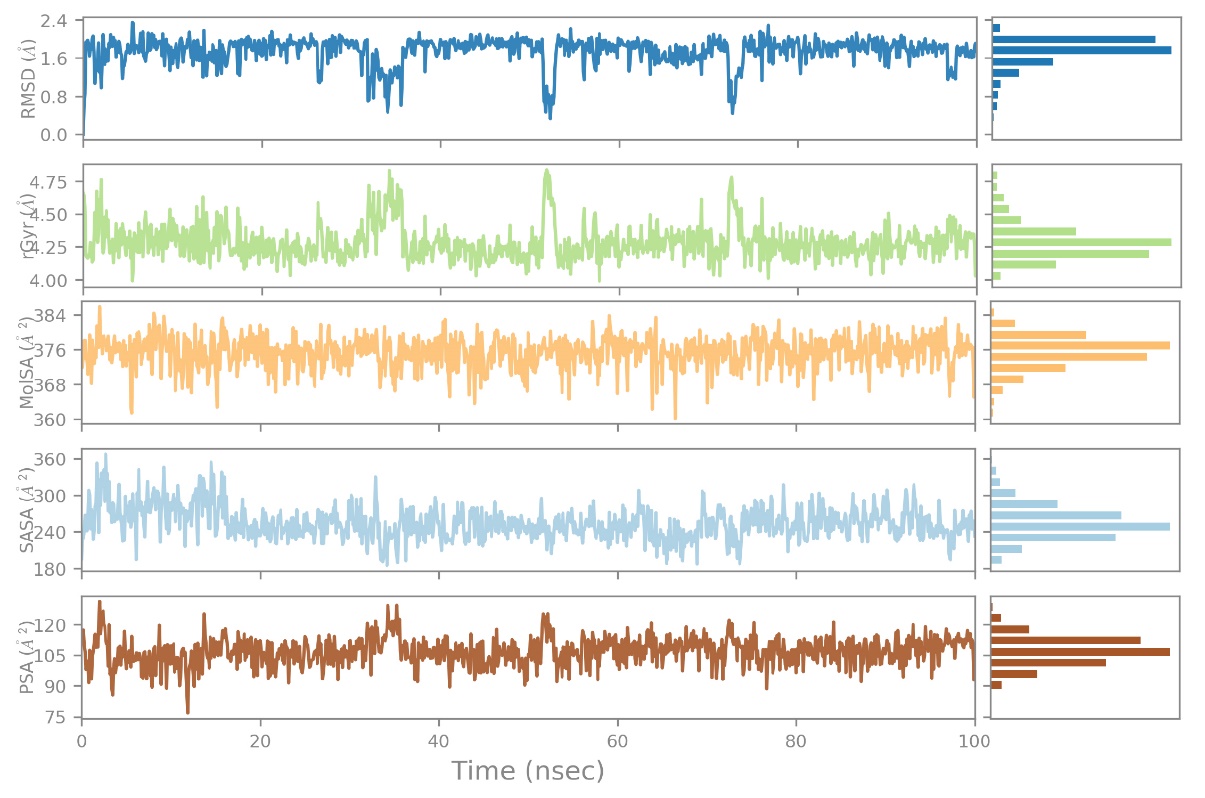


**Figure C.** The ligand property trajectory of the (VEGFR-2 complex with best established compound PubChem ID:369976) during the 100 ns simulation.


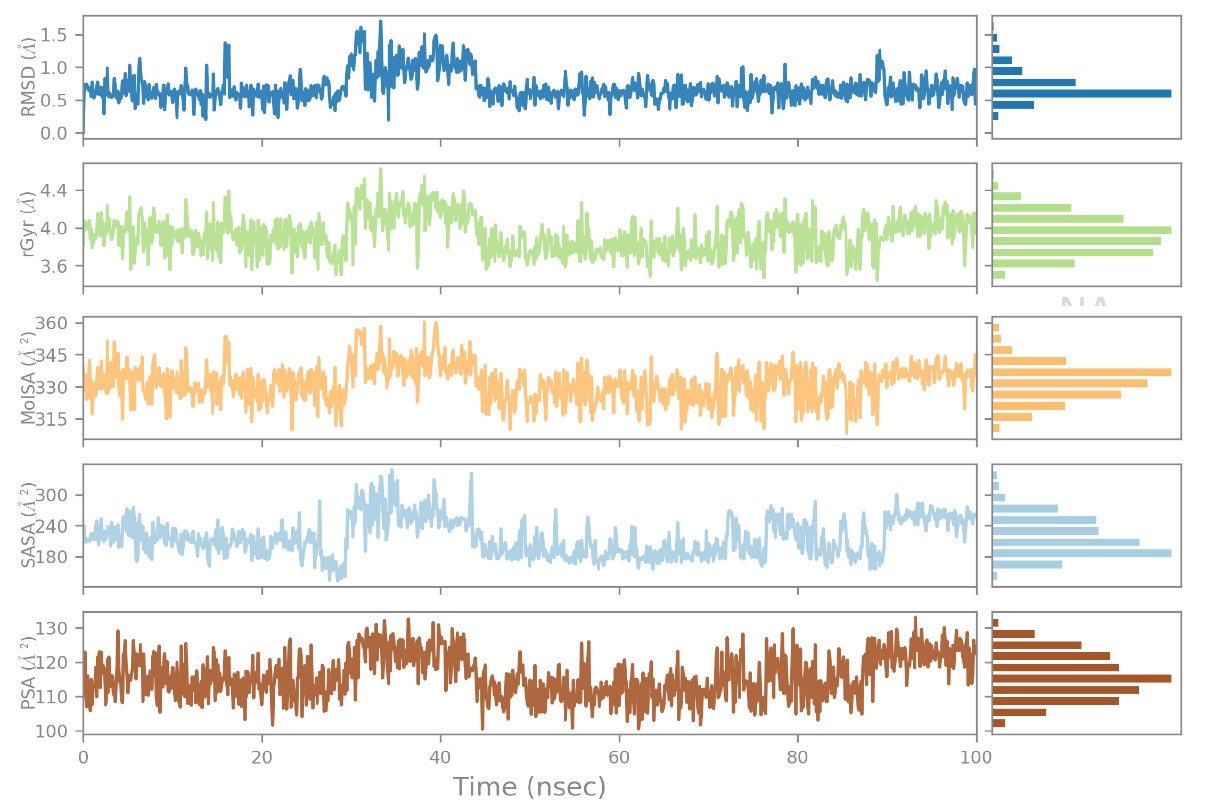


**Figure D.** The ligand property trajectory of the (VEGFR-2 complex with best ML Model compound PubChem ID:11152946) during the 100 ns simulation.


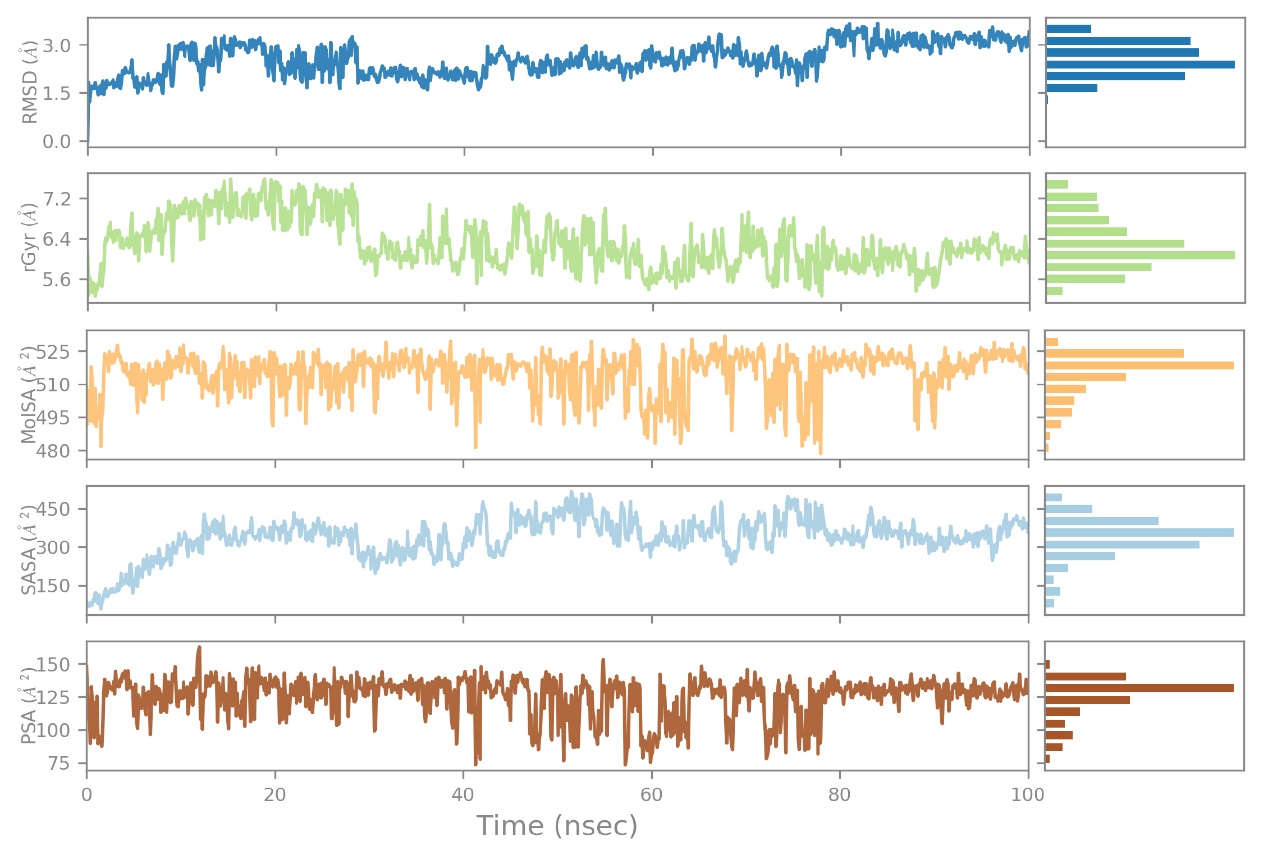


**Figure E.** The ligand property trajectory of the (VEGFR-3 complex with best established compound PubChem ID:208908) during the 100 ns simulation.


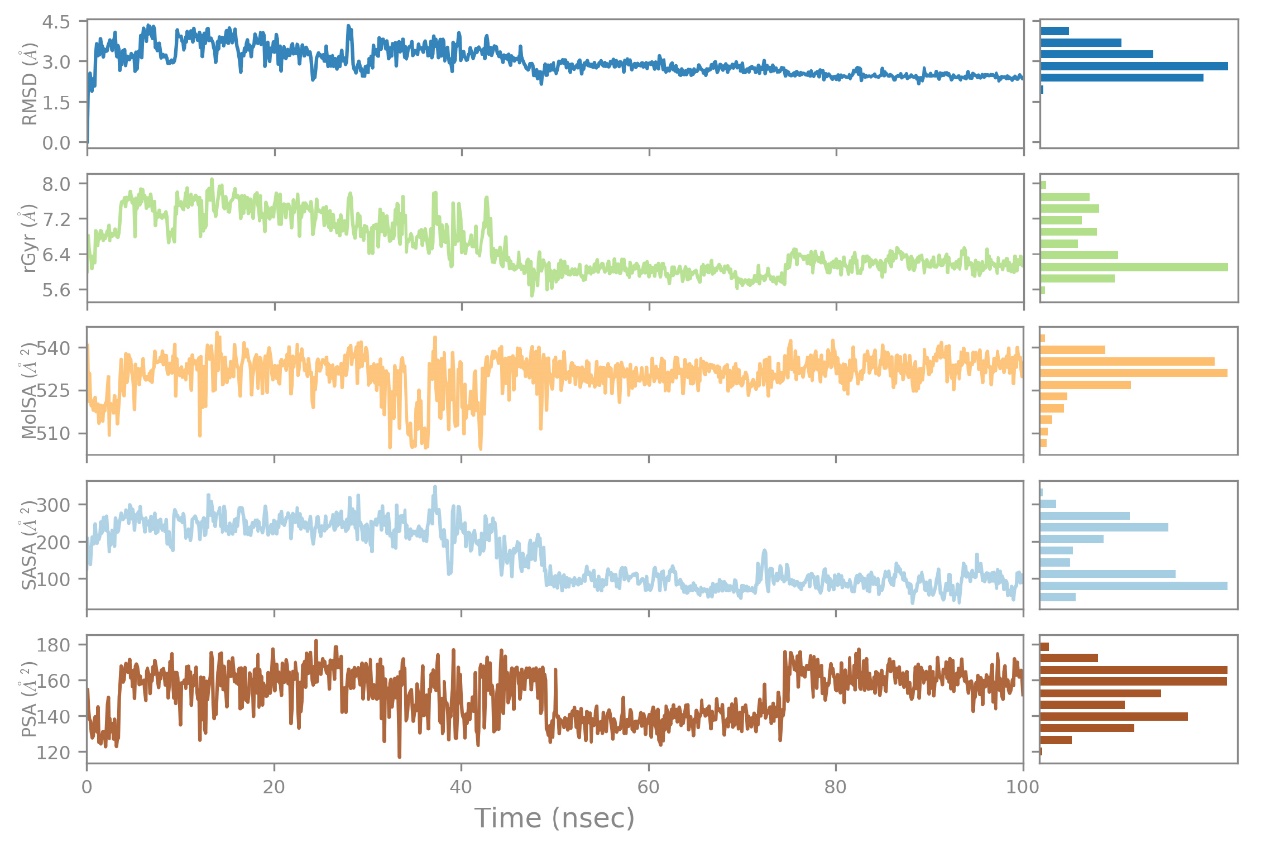


**Figure F.** The ligand property trajectory of the (VEGFR-3 complex with best ML Model compound PubChem ID: 68155180) during the 100 ns simulation.
