# Supplementary material for: Potential inhibitors of VEGFR1, VEGFR2, and VEGFR3 developed through Deep Learning for the treatment of Cervical Cancer
Source: Sci Rep. 2024 Jun 10;14:13251. doi: 10.1038/s41598-024-63762-w (PMC11164920; doi:10.1038/s41598-024-63762-w)
Supplement: Supplementary file 4 — Supplementary Data 4. [file 41598_2024_63762_MOESM4_ESM.docx]

**Supplementary Data IV**


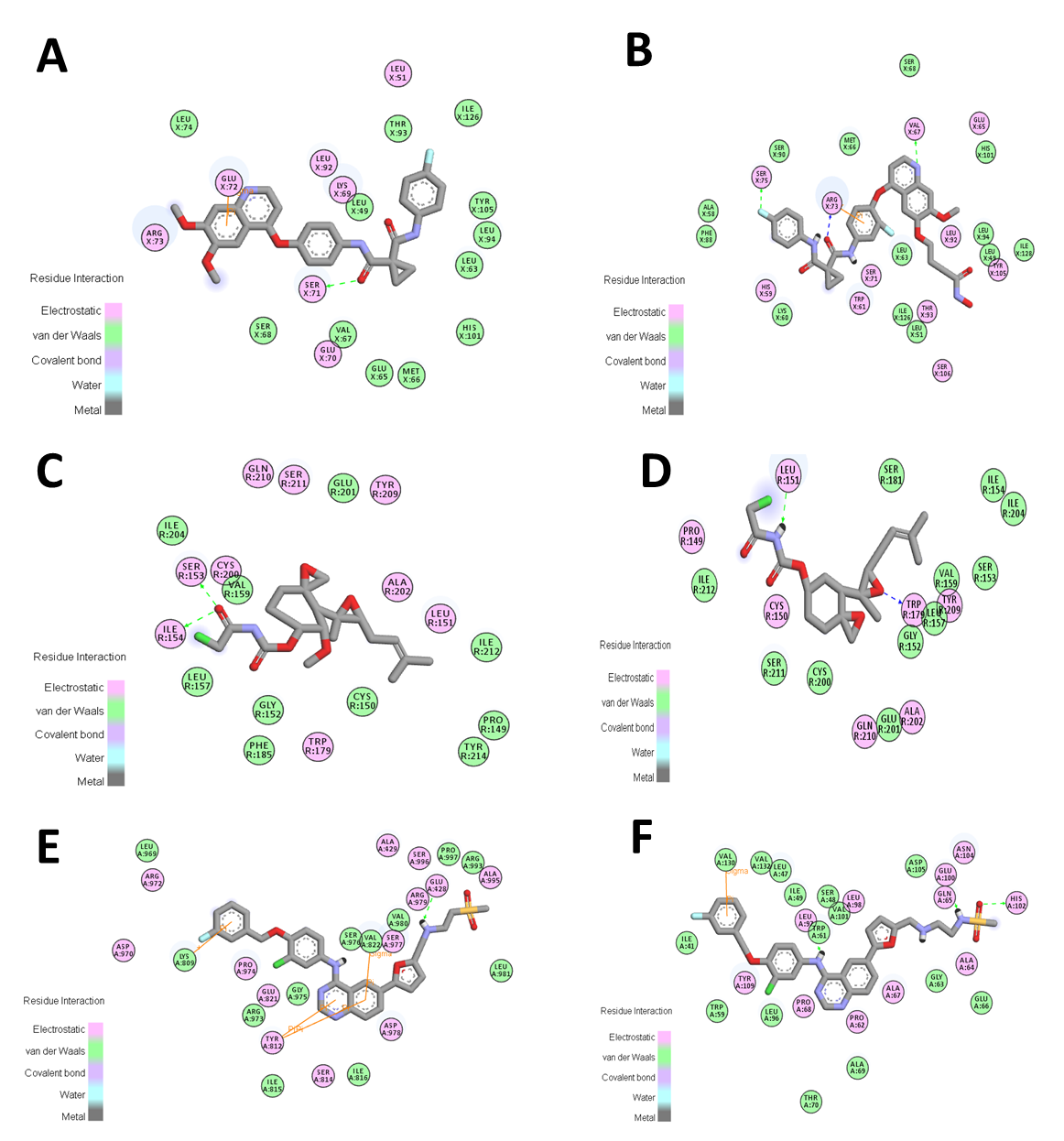


**Figure I.** 2D Interactions of the most effective established compound and ML generated compound towards VEGFR-I, VEGFR-II and VEGFR-III – A. The most effective established compound Cabozantinib shows Van der Waals interactions with VEGFR-1, B. The most effective machine learning model compound PubChem ID: 71465645 shows Van der Waals interactions with VEGFR-1; C. The most effective established compound PubChem IDL 369976 shows Van der Waals interactions with VEGFR-2, D. The most effective machine learning model compound PubChem ID: 11152946, shows Van der Waals interactions with VEGFR-2; E. The most effective established compound PubChem CID: 208908 shows Van der Waals interactions with VEGFR-3, F. The most effective machine learning model compound PubChem CID: 68155180 shows Van der Waals interactions with VEGFR-3.
