# Supplementary material for: Potential inhibitors of VEGFR1, VEGFR2, and VEGFR3 developed through Deep Learning for the treatment of Cervical Cancer
Source: Sci Rep. 2024 Jun 10;14:13251. doi: 10.1038/s41598-024-63762-w (PMC11164920; doi:10.1038/s41598-024-63762-w)
Supplement: Supplementary file 5 — Supplementary Data 5. [file 41598_2024_63762_MOESM5_ESM.docx]

**Supplementary Data V**

**Docking procedure Validation Analysis –**

In the initial phase, we subjected the top hits compounds identified from the docking studies to a rigorous evaluation using the enrichment calculator in Schrödinger. This assessment involved the implementation of an extra precision docking procedure in conjunction with a decoy set. The outcome of this analysis revealed a significant receiver operating characteristic curve (ROC) value of 0.94. In the context of docking protocols, an ROC value exceeding 0.7 is conventionally considered indicative of a robust and reliable performance. Therefore, our achieved ROC value of 0.94 underscores a high level of confidence in the precision and efficacy of our docking protocol. This finding reinforces the credibility of our results and underscores the meticulous nature of our approach in identifying and prioritizing compounds.


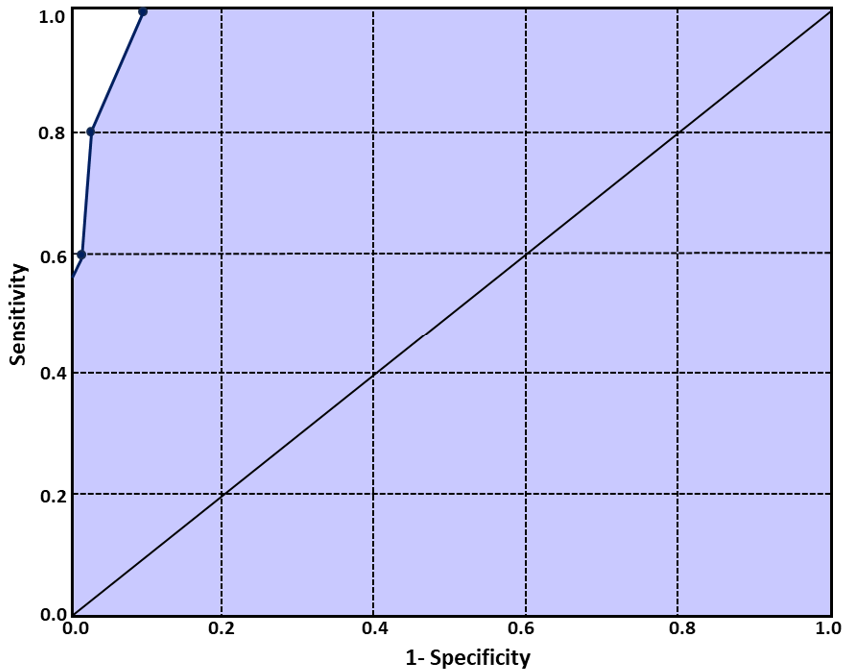


**Figure I.** Validation analysis of docking procedure by enrichment analysis.
