# Supplementary material for: Potential inhibitors of VEGFR1, VEGFR2, and VEGFR3 developed through Deep Learning for the treatment of Cervical Cancer
Source: Sci Rep. 2024 Jun 10;14:13251. doi: 10.1038/s41598-024-63762-w (PMC11164920; doi:10.1038/s41598-024-63762-w)
Supplement: Supplementary file 6 — Supplementary Table 1. [file 41598_2024_63762_MOESM6_ESM.docx]

**Supplementary Table 1**

**List of Established VEGFR inhibitors with their PubChem ID.**

(HBD: H-Bond Donor; HBA: H-Bond Acceptor; √: present; x: Absent)

| **S.No** | **Inhibitor** | **VEGFR1** | **VEGFR2** | **VEGFR 3** | **PubChemID** | **MW (g/mol)** | **HBD** | **HBA** | **Ref** |
| --- | --- | --- | --- | --- | --- | --- | --- | --- | --- |
| 1 | Brivanib | x | √ | x | 11234052 | 370.4 | 2 | 6 | [19] |
| 2 | Pazopanib | √ | √ | √ | 10113978 | 437.5 | 2 | 8 | [20] |
| 3 | lapatinib | x | √ | x | 208908 | 581.1 | 2 | 9 | [21] |
| 4 | Cediranib | √ | √ | √ | 9933475 | 450.5 | 1 | 7 | [22] |
| 5 | Apatinib | x | √ | x | 11315474 | 397.5 | 2 | 5 | [23] |
| 6 | Sunitinib | √ | √ | √ | 5329102 | 398.5 | 3 | 4 | [24] |
| 7 | Anlotinib | x | √ | √ | 25017411 | 407.4 | 2 | 6 | [32] |
|  | AEE788 | x | √ | x | 10297043 | 440.6 | 2 | 5 | [33] |
| 7 | Axitinib | √ | √ | √ | 6450551 | 386.5 | 2 | 4 | [34] |
| 8 | SU5416/Semaxanib | x | √ | x | 5329098 | 238.28 | 2 | 1 | [35] |
| 11 | tivozanib | x | √ | x | 9911830 | 454.9 | 2 | 7 | [36] |
| 12 | CGP 41251 | √ | √ | x | 9829523 | 570.6 | 1 | 4 | [37] |
| 13 | Decursin | x | √ | x | 442126 | 328.4 | 0 | 5 | [38-42] |
| 14 | Oleanolic Acid | √ | √ | √ | 10494 | 456.7 | 2 | 3 | [43-44] |
| 15 | Esculetin | x | √ | x | 5281416 | 178.14 | 2 | 4 | [45] |
| 16 | Decursinol Angelate | x | √ | x | 776123 | 328.4 | 0 | 5 | [46] [47] |
| 17 | Daphnetin | x | √ | x | 5280569 | 178.14 | 2 | 4 | [46] [47] |
| 18 | Aviprin | x | √ | x | 17536 | 304.29 | 2 | 6 | [48] |
| 19 | TNP-470 | x | √ | x | 369976 | 401.9 | 1 | 6 | [49-50] |
| 20 | Nintedanib | √ | √ | √ | 135423438 | 539.6 | 2 | 7 | [51][52] |
| 21 | AAL993 | x | √ | x | 6398883 | 371.4 | 2 | 6 | [53] |
| 22 | KRN633 | x | √ | x | 9549295 | 416.9 | 2 | 6 | [53] |
| 23 | ZD6474 | x | √ | x | 3081361 | 475.4 | 1 | 7 | [53] |
| 24 | 7-(4′-methylpentoxy)-2H-1-benzopyran-2-one | x | √ | x | 137332191 | 246.3 | 0 | 3 | [54] |
| 25 | Vatalanib/ PTK787 | √ | √ | √ | 151194 | 346.8 | 1 | 4 | [55] |
| 26 | Cabozantinib | x | √ | x | 25102847 | 501.5 | 2 | 7 | [56] |
