# Supplementary material for: Potential inhibitors of VEGFR1, VEGFR2, and VEGFR3 developed through Deep Learning for the treatment of Cervical Cancer
Source: Sci Rep. 2024 Jun 10;14:13251. doi: 10.1038/s41598-024-63762-w (PMC11164920; doi:10.1038/s41598-024-63762-w)
Supplement: Supplementary file 7 — Supplementary Table 2. [file 41598_2024_63762_MOESM7_ESM.docx]

**Supplementary Table 2**

**Drug-Drug Comparative study of best-established Vs ML Model compounds towards VEGFR-1, VEGFR-2 and VEGFR-3**

**Table A.**Drug-Drug Comparative study of best-established Vs ML Model compounds towards VEGFR-1.

|  | **Best Established compound**  **(PubChem ID:25102847)** | | **ML Model compound**  **(PubChem ID: 71465645)** | |
| --- | --- | --- | --- | --- |
| **Energy overview: Descriptors** | **MolDock Score** | **Re-rank Score** | **MolDock Score** | **Re-rank Score** |
| **Total Energy** | -155.779 | -112.901 | -169.808 | -143.113 |
| **External Ligand interactions** | -174.94 | -141.832 | -206.4 | -172.759 |
| **Protein - Ligand interactions** | -174.94 | -141.832 | -206.4 | -172.759 |
| **Steric (by PLP)** | -171.912 | -117.931 | -200.603 | -137.614 |
| **Steric (by LJ12-6)** | - | -21.502 | - | -30.554 |
| **Hydrogen bonds** | -3.028 | -2.398 | -5.797 | -4.591 |
| **Internal Ligand interactions** | 38.523 | 41.139 | 36.009 | 38.801 |
| **Torsional strain** | 16.436 | 15.417 | 12.233 | 11.474 |
| **Torsional strain (sp2-sp2)** | - | 4.417 | - | 3.174 |
| **Steric (by PLP)** | 24.588 | 4.229 | 28.638 | 4.926 |
| **Steric (by LJ12-6)** | - | 17.077 | - | 19.228 |

**Table B.**Drug-Drug Comparative study of best-established Vs ML Model compounds towards VEGFR-2.

|  | **Established best compound**  **(PubChem ID:369976)** | | **ML Model compound**  **(PubChem ID: 11152946)** | |
| --- | --- | --- | --- | --- |
| **Energy overview: Descriptors** | **MolDock Score** | **Re-rank Score** | **MolDock Score** | **Re-rank Score** |
| **Total Energy** | -119.137 | -90.397 | -115.968 | -112.61 |
| **External Ligand interactions** | -135.766 | -108.639 | -124.555 | -103.27 |
| **Protein - Ligand interactions** | -135.766 | -108.639 | -124.555 | -103.27 |
| **Steric (by PLP)** | -129.647 | -88.938 | -120.948 | -82.97 |
| **Steric (by LJ12-6)** | - | -14.855 | - | -17.443 |
| **Hydrogen bonds** | -6.119 | -4.846 | -3.607 | -2.856 |
| **Internal Ligand interactions** | 16.63 | 18.241 | 8.594 | 8.927 |
| **Torsional strain** | 8.465 | 7.94 | 4.63 | 4.343 |
| **Torsional strain (sp2-sp2)** | - | 0.156 | - | 0.115 |
| **Steric (by PLP)** | 8.165 | 1.404 | 3.964 | 0.682 |
| **Steric (by LJ12-6)** | - | 8.741 | - | 3.787 |

**Table C.**Drug-Drug Comparative study of best-established Vs ML Model compounds towards VEGFR-3.

|  | | **Established best compound**  **(PubChem ID:208908)** | | **ML Model compound**  **(PubChem ID: 68155180)** | |
| --- | --- | --- | --- | --- | --- |
| **Energy overview: Descriptors** | | **MolDock Score** | **Re-rank Score** | **MolDock Score** | **Re-rank Score** |
| **Total Energy** | | -155.94 | -112.91 | -198.138 | -151.12 |
| **External Ligand interactions** | | -174.959 | -139.25 | -221.592 | -188.301 |
| **Protein - Ligand interactions** | | -174.959 | -139.25 | -221.592 | -188.301 |
| **Steric (by PLP)** | | -167.928 | -115.199 | -213.878 | -146.72 |
| **Steric (by LJ12-6)** | **-** | | -18.482 | - | - |
| **Hydrogen bonds** | | -7.031 | -5.568 | -7.714 | -6.11 |
| **Internal Ligand interactions** | | 19.019 | 26.34 | 23.007 | 40.413 |
| **Torsional strain** | | 6.276 | 5.887 | 17.255 | 16.185 |
| **Torsional strain (sp2-sp2)** | **-** | | 1.031 | - | - |
| **Steric (by PLP)** | | 17.128 | 2.946 | 10.511 | 1.808 |
| **Steric (by LJ12-6)** | **-** | | 16.476 | - | - |
