# Supplementary material for: Potential inhibitors of VEGFR1, VEGFR2, and VEGFR3 developed through Deep Learning for the treatment of Cervical Cancer
Source: Sci Rep. 2024 Jun 10;14:13251. doi: 10.1038/s41598-024-63762-w (PMC11164920; doi:10.1038/s41598-024-63762-w)
Supplement: Supplementary file 8 — Supplementary Table 3. [file 41598_2024_63762_MOESM8_ESM.docx]

**Supplementary Table III**

ADMET profile analysis of the best compounds obtained from Machine Learning –

|  | **PubChem ID: 71465645** | **PubChem CID: 11152946** | **PubChem ID: 68155180** |
| --- | --- | --- | --- |
| **Ames mutagenesis** | - | - | - |
| **Acute Oral Toxicity (c)** | III | III | III |
| **Androgen receptor binding** | + | - | + |
| **Aromatase binding** | + | + | + |
| **Avian toxicity** | - | - | - |
| **Blood Brain Barrier** | + | + | + |
| **BRCP inhibitor** | - | - | + |
| **Biodegradation** | - | - | - |
| **BSEP inhibitor** | + | + | + |
| **Caco-2** | - | - | - |
| **Carcinogenicity (binary)** | - | - | - |
| **Carcinogenicity (trinary)** | Non-required | Non-required | Non-required |
| **crustacea aquatic toxicity** | - | + | + |
| **CYP1A2 inhibition** | - | - | + |
| **CYP2C19 inhibition** | - | - | + |
| **CYP2C9 inhibition** | - | - | - |
| **CYP2C9 substrate** | - | + | - |
| **CYP2D6 inhibition** | - | - | - |
| **CYP2D6 substrate** | - | - | - |
| **CYP3A4 inhibition** | + | - | + |
| **CYP3A4 substrate** | + | + | + |
| **CYP inhibitory promiscuity** | + | - | + |
| **Eye corrosion** | - | - | - |
| **Eye irritation** | - | - | - |
| **Estrogen receptor binding** | + | + | + |
| **Fish aquatic toxicity** | - | + | + |
| **Glucocorticoid receptor binding** | + | + | + |
| **Honeybee toxicity** | - | + | - |
| **Hepatotoxicity** | + | - | + |
| **Human either-a-go-go inhibition** | + | - | + |
| **Human Intestinal Absorption** | + | + | + |
| **Human oral bioavailability** | + | - | - |
| **MATE1 inhibitor** | - | - | - |
| **micronuclear** | + | + | + |
| **Acute Oral Toxicity** | 3.622593164 | 2.154577017 | 3.982810736 |
| **OATP1B1 inhibitor** | + | + | + |
| **OATP1B3 inhibitor** | + | + | + |
| **OATP2B1 inhibitor** | + | - | + |
| **OCT1 inhibitor** | - | - | - |
| **OCT2 inhibitor** | - | - | - |
| **P-glycoprotein inhibitor** | + | - | + |
| **P-glycoprotein substrate** | + | - | + |
| **PPAR gamma** | + | + | + |
| **Plasma protein binding** | 1.191219091 | 0.894399226 | 1.239817143 |
| **Subcellular localization** | Mitochondria | Mitochondria | Lysosomes |
| **Tetrahymena pyriformis** | 1.538282394 | 0.406438589 | 1.397502422 |
| **Thyroid receptor binding** | + | + | + |
| **UGT catalysed** | - | - | - |
| **Water solubility** | -3.417666268 | -3.809252204 | -3.623193587 |
